# Acceleration and Selectivity of 1,3-Dipolar Cycloaddition Reactions Included in a Polar [4 + 2] Octa-imine Bis-calix[4]pyrrole Cage

**DOI:** 10.1021/jacsau.4c01118

**Published:** 2025-01-23

**Authors:** Yifan Li, Chiara F. M. Mirabella, Gemma Aragay, Pablo Ballester

**Affiliations:** † Institute of Chemical Research of Catalonia (ICIQ), The Barcelona Institute of Science and Technology (BIST), Avgda. Països Catalans, 16, 43007 Tarragona, Spain; ‡ Departament de Química Analítica i Química Orgànica, Universitat Rovira i Virgili, c/Marcel·lí Domingo,1, 43007 Tarragona, Spain; § ICREA, Passeig Lluís Companys, 23, 08010 Barcelona, Spain

**Keywords:** molecular container, dynamic covalent cage, reactor vessel, click chemistry, calix[4]pyrrole

## Abstract

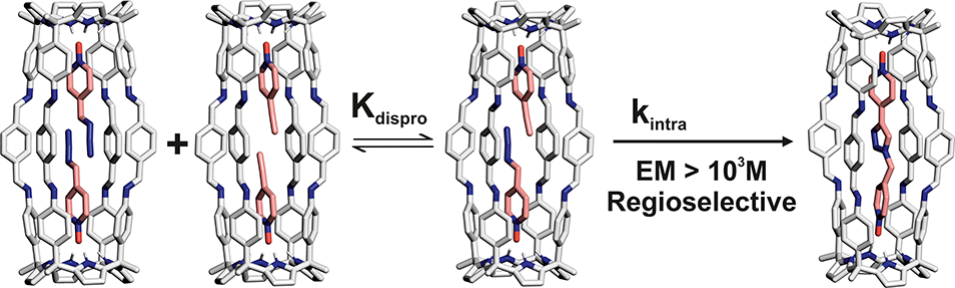

We describe the quantitative self-assembly (>90%) of a [4 + 2] octa-imine cage (**1**) in a CDCl_3_:CD_3_CN 9:1 solvent mixture containing 0.5% of acetic acid. Cage **1** is based on two identical aryl-extended calix[4]pyrrole units linked through eight dynamically reversible imine bonds. Cage **1** forms thermodynamically and kinetically highly stable inclusion complexes featuring 1:1 and 2:1 stoichiometry with suitable *para*-substituted pyridine-*N*-oxides. The ability of **1** for the pairwise inclusion of two different pyridine-*N*-oxides led us to investigate its properties as a reactor vessel. The coinclusion of 4-azido pyridine-*N*-oxide and 4-ethynyl pyridine-*N*-oxide did not produce a detectable acceleration of their 1,3-dipolar cycloaddition reaction. Conversely, the coinclusion in cage **1** of the same alkyne dipolarophile with 4-azido(alkyl) pyridine-*N*-oxides (alkyl= methyl, ethyl) produced significant reaction acceleration. We quantified the reactions’ acceleration with an effective molarity (EM) of ∼10^3^ M, corresponding to the more prominent reported value of a bimolecular 1,3-dipolar cycloaddition reaction in a molecular vessel by directly detecting the ternary Michaelis complex. The included reactions are quantitative and regioselective, yielding exclusively the 1,4-disubstituted triazole isomers. We propose that the selectivity of **1** in accelerating the included 1,3-dipolar cycloadditions is related to (a) the entropy gain provoked by the reaction’s inclusion, (b) the rigidity of the container, and (c) the spatial fixation of the polar knobs (pyridine-*N*-oxide) carrying the reacting groups in its two functionalized hemispheres. The two latter characteristics render the distance between the reacting groups (azido and ethynyl) almost fixed by design, thus allowing or not achieving the transition state’s geometry. We support our hypothesis with the help of DFT calculations of the inclusion complexes’ structures.

## Introduction

The confinement of molecules in nanometric spaces can change their physical and chemical properties. Enzymes bind substrates in confined environments, *a.k.a* active sites, to catalyze their chemical transformations under mild conditions.^1,2^ Moreover, the reacting groups of the bound substrates are placed in close proximity and adopt a suitable orientation to react. This results in an effective increase in the concentration of the reactants and endows intermolecular reactions with an intramolecular character, resulting in a significant rate increase. Page and Jencks quantified the entropic contribution to rate acceleration for bimolecular enzymatic reactions up to −35 e.u. (corresponding to a rate acceleration EM = e^ΔS/R^ = 4 × 10^7^ M) or more.^3^ It is also proposed that enzymes bind elusive transition states more tightly than either substrates or products, causing additional rate acceleration.^4−6^

Synthetic molecular containers stabilize highly reactive species and high-energy conformations by including substrates in their cavities. Likewise, the inclusion of reactions in molecular containers led to altered pathways, changes in selectivity, and noticeable rate increases when compared to those in bulk solution.^7−9^

In particular, the acceleration experienced by cycloaddition reactions upon inclusion in covalent, supramolecular, and biological synthetic molecular containers was used to assess their potential as synthetic enzymes.^10^ Several examples of Diels–Alder (DA) reactions accelerated inside the cavity of molecular containers were reported.^11−17^ The inclusion of reactants in molecular containers can also influence the diastereoselectivity of the DA reaction (i.e., “endo”/“exo” products ratio).^10^ In most of the reported examples of DA reactions inside molecular containers, either one or both reactants contained heteroatoms, and the maximum acceleration value measured as EM = *k*_intra_/*k*_bulk_ was ≤10^3^ M. Recently, Nau and co-workers estimated that performing the cyclopentadiene dimerization inside cucurbit[7]uril led to an acceleration factor of ca. 4 × 10^5^ M, which is close to the predicted maximum.^3,18^

Notably, the acceleration of Huisgen cycloadditions by inclusion in molecular containers has been less explored. In bulk, the Huisgen cycloaddition requires elevated temperatures. It often produces a mixture of two 1,2,3-triazole regioisomers (1,4 and 1,5).^19,20^ Copper^21^ or ruthenium-^22^ catalyzed alternatives are well-known to selectively obtain the 1,4 or the 1,5-disubstituted 1,2,3-triazole isomers, respectively.

In the nineties, Mock et al. described the acceleration of intermolecular cycloadditions between azide and alkyne ammonium derivatives included in cucurbit[6]uril.^23,24^ The reaction of *N*-*tert*-butylated substrates produced a product with [2]rotaxane topology, simplifying the analysis of the kinetic data. From the reported data, it was derived that the reaction’s acceleration factor expressed as EM could be estimated as ca. 1.9 × 10^4^ M.^25,26^ The lack of direct detection of the ternary complex (Michaelis) made the kinetic data analysis and the derivatization of the EM value not straightforward.

In 2002, Rebek and Jia disclosed that the cycloaddition between phenyl azide and phenyl acetylene was accelerated when included in a self-assembled resorcin[4]arene capsular dimer.^27^ Cacciapaglia and co-workers calculated the EM acceleration factor to be 120 M.^25^ In this case, the ternary complex was observed, simplifying the kinetic data analysis and the derivatization of an EM value.

It is worth noting that the Huisgen reactions proceeding in the interior of CB6 and the self-assembled capsular dimer were regioselective, yielding exclusively the 1,4-isomer of the triazole products. The results of theoretical calculations of the reactions taking place inside these containers assigned entropic effects and geometric strain between reactants as the most relevant factors for the observed reaction accelerations.^28,29^ The putative increase in pressure experienced by the included reactants might also accelerate the reactions owing to their known negative activation volumes.^18^

A few years ago, our group described the template-directed synthesis of octa-imine cages based on aryl-extended calix[4]pyrrole units.^30^ A unique feature of these reported cages was that their hemispheres were defined by two endohedral and converging polar binding sites. The 4,4′-bis-pyridine-*N*,*N*′-dioxide resulted in a perfect template for the quantitative assembly of the dynamically covalent cages but could not be removed afterward. More recently, we published the self-assembly of a related [1 + 1] tetra-imine calix[4]pyrrole cage in a 9:1 CDCl_3_:CD_3_CN solvent mixture.^31^ The dynamic tetra-imine covalent cage was assembled in a good yield. Using weakly bound CD_3_CN molecules as the template for the cage’s assembly allowed the study and characterization of its binding properties with pyridine-*N*-oxide guests.

**Scheme 1:**
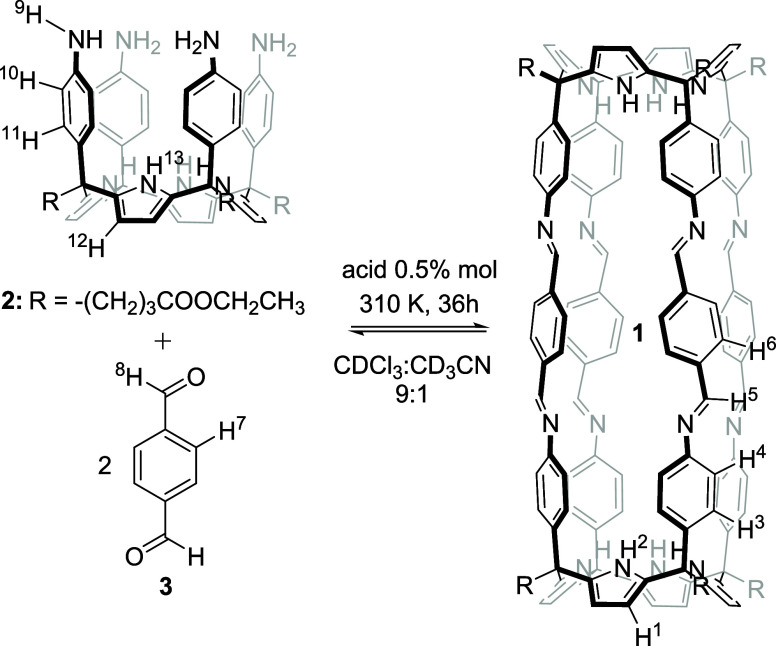
Equilibrium of the Self-Assembly Process of the [2 + 4] Octa-imine Cage **1** The proton assignment for cage **1** and its synthetic precursors, tetra-amine **2** and terephthalaldehyde **3**, is also shown.

**Scheme 2:**
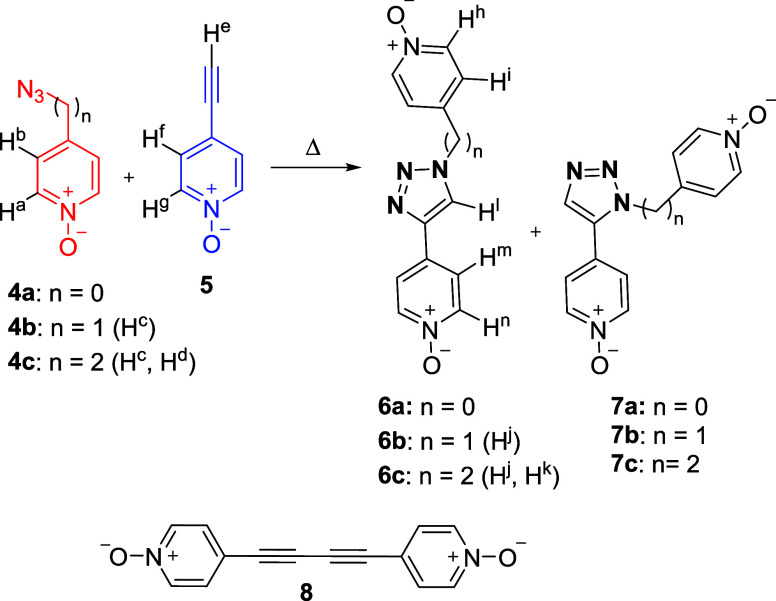
(Top) Scheme of the 1,3-Dipolar Cycloaddition Reaction between Azido (**4a**, **4b** or **4c**) and Ethynyl (**5**) Derivatives Producing the Two Regioisomeric 1,4- (**6**) and 1,5- (**7**) Disubstituted 1,2,3-Triazoles (The Corresponding Proton Assignment for **4a**–**c**, **5**, and **6a**–**c** Is Shown); (Bottom) Molecular Structure of 4,4-(Buta-1,3-diyne-1,4-diyl)bis-pyridine-*N*-Oxide **8** Used as a Template in the Self-Assembly of the Octa-imine Cage **1** Is Also Shown

Herein, we report the self-assembly of a much larger [4 + 2] octa-imine bis-calix[4]pyrrole cage **1** featuring two identical endohedral functionalized hemispheres (Scheme 1). We describe the binding properties of cage **1** with several pyridine-*N*-oxide guests functionalized at their *para-*position with acetylene and azido groups. We investigate the kinetics of the 1,3-dipolar cycloaddition reaction between azido- and alkyne-derived pyridine-*N*-oxides (Scheme 2) included in the cavity of **1** (_intra_) and compare them with those in bulk (_bulk_). Some reactions experienced acceleration factors, quantified as effective molarity, EM= *k*_intra_/*k*_bulk_, larger than 10^3^ M. The systems do not exhibit turnover but feature the largest acceleration value described for bimolecular reactions included in molecular vessels, for which the direct detection of the ternary complex (Michaelis) was possible. Our results demonstrate that substrate binding and confinement in a synthetic molecular container produced significant accelerations of bimolecular Huisgen cycloaddition reactions.

## Synthesis

### Pyridine-*N*-oxide Derivatives

We uneventfully prepared pyridine-*N*-oxide derivatives **4a**-**d** using slightly modified reported methodologies (Scheme 2, see SI for experimental details).^32−34^ We performed the Cu(I) catalyzed cycloaddition reactions of the *N-*oxides **4a, 4b**, and **4c**, with **5**, by standard literature procedures.^21^ Using preparative TLC, we isolated the corresponding 1,4- and 1,5- triazole isomers, **6** and **7**. We also synthesized the bis-*N*-oxide **8** by dimerization of **5** using Hay reaction conditions.^35^ The prepared *N*-oxides were used as guests in the binding studies and kinetic experiments of cage **1** (vide infra).

### Synthetic Precursors for the Self-Assembly of Cage **1**

Tetra-amine tetra-ester aryl-extended calix[4]pyrrole **2** (AE-C[4]P **2**) was prepared using a procedure reported by us.^36^ Terephthalaldehyde **3** was commercially purchased.

## Results and Discussion

### Self-Assembly of [2 + 4] Octa-imine Cage **1**

#### Octa-imine Cage **1**

**Figure 1:**
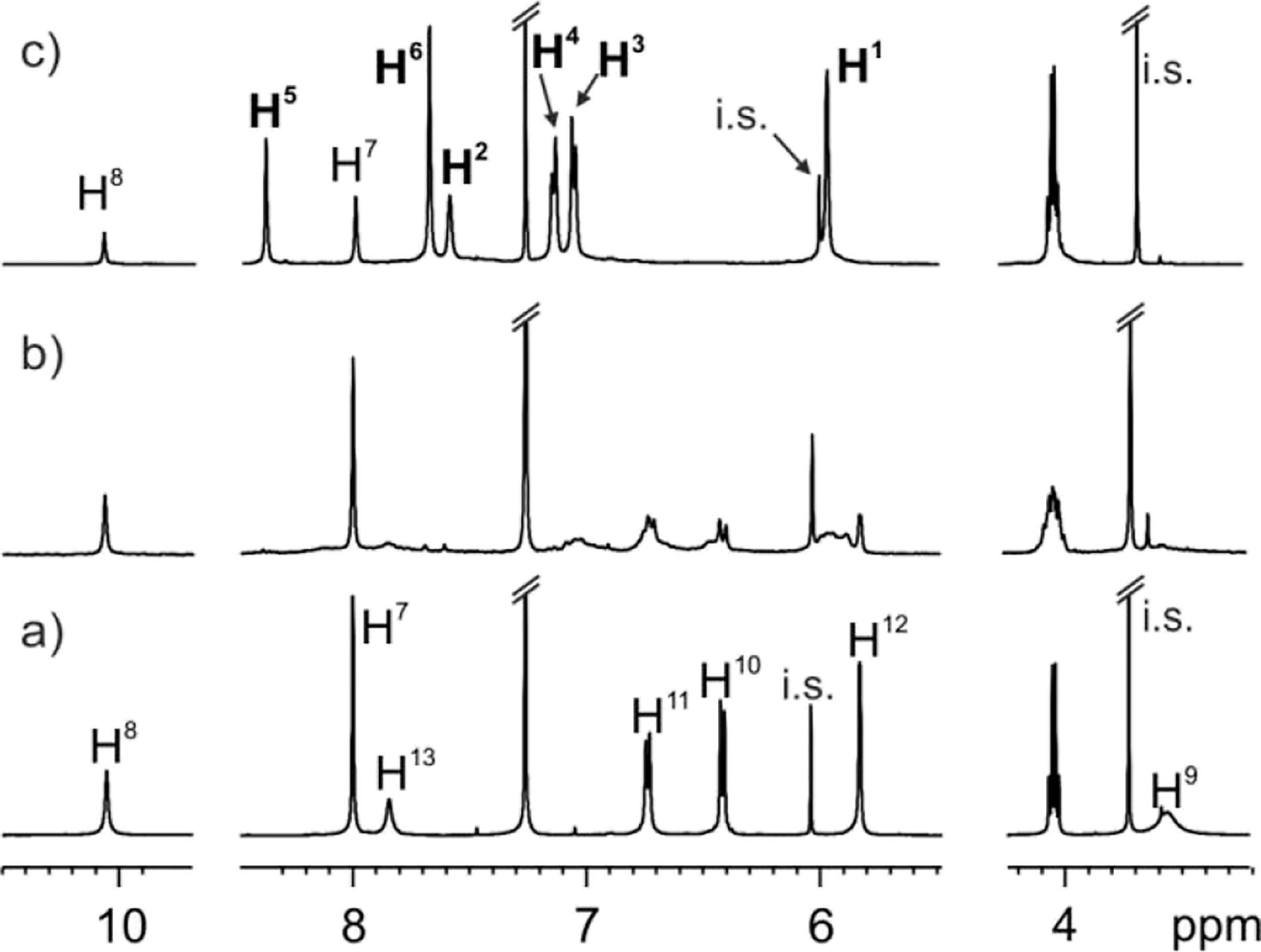
Selected regions of the ^1^H HMR spectra (400 MHz, 298 K, CDCl_3_:CD_3_CN 9:1 mixture) of solutions containing (a) tetra-amine AE-C[4]P **2** (2 mM) and terephthalaldehyde **3** (4.4 mM); (b) same mixture of (a), following the addition of 0.5% mol of acetic acid; (c) same mixture of (b) after 36 h. See Scheme 1 for the proton assignment. Hydrogen atoms in cage **1** are depicted in bold format for clarity. i.s. internal standard.

We dissolved freshly distilled terephthalaldehyde **3** (0.25 mL 8.8 mM, 2.2 equiv) and tetra-amine tetra-ester calix[4]pyrrole **2** (0.25 mL 4 mM, 1 equiv) in a CDCl_3_:CD_3_CN 9:1 solvent mixture (0.5 mL) containing 1,3,5-trimethoxybenzene as internal standard (i.s.). The resulting solution was placed in a J Young NMR tube and analyzed at different time intervals using ^1^H NMR spectroscopy (Figure 1). The initial ^1^H NMR spectrum of the mixture showed sharp and well-defined signals corresponding to the hydrogen atoms of the unreacted starting materials (Figure 1a). Following the addition of 0.5% acetic acid, the ^1^H NMR spectrum of the mixture evidenced the appearance of broad signals and the concomitant decrease in intensity of the signals corresponding to the tetra-amine AE-C[4]P **2** and the bis-aldehyde **3** (Figure 1b). This observation suggested the formation of oligomeric species linked by imine bonds of unknown stoichiometry and ill-defined structures. The solution mixture was allowed to stand at r.t. After 36 h, the ^1^H NMR spectrum of the mixture revealed the appearance of a new set of sharp and well-defined proton signals (Figure 1c). We did not detect any of the signals corresponding to the protons of the tetra-amine AE-C[4]P **2**. In contrast, we observed residual signals of the protons of bis-aldehyde **3** (H^7^ and H^8^).^37^

The new set of proton signals agreed with the quantitative self-assembly of the octa-imine cage **1**, displaying an apparent *D*_4*h*_ symmetry (Scheme 1). We attributed the singlet resonating at δ = 8.4 ppm to the imine protons (H^5^). The pyrrole NHs (H^2^) appeared as a broad singlet centered at δ = 7.6 ppm. The aromatic protons of the spacer panels (H^6^) produced a singlet at δ = 7.7 ppm, while those of the *meso*-aryl substituents (H^3^ and H^4^) gave two *ortho*-coupled doublets, at δ = 7.0 and 7.1 ppm. A 2D ROESY experiment evidenced the existence of several cross-peaks due to close intramolecular contact in agreement with the octa-imine cage **1** structure and the proton assignment (Figure S3). Octa-imine cage was characterized by a complete set of high-resolution spectra (NMR and HRMS, Figures S1–S5).

Using the integral values of the internal standard (i.s.) as a reference, we determined that the self-assembly of the octa-imine cage **1** proceeded in almost quantitative yield (>90%).^38^ Previously, we showed that in the solid-state, structurally related mononuclear Pd(II) and Pt(II) C[4]P cages self-assembled in the same solvent mixture by including acetonitrile molecules in their cavities.^39^ The cone conformation of a “four wall” AE-C[4]P represents a perfect fit for including an acetonitrile molecule. The nitrogen atom of the included acetonitrile forms four convergent hydrogen bonds with the pyrrole NHs of the C[4]P cone conformation. Molecular modeling studies showed that for the octa-imine cage **1**, one CD_3_CN molecule could be included in each one of its two C[4]P hemispheres, resulting in the solvate cage complex (CD_3_CN)_2_⊂**1** having its middle aromatic cavity collapsed.^40^

Next, we studied the self-assembly of the octa-imine cage **1** in CDCl_3_ solution using 0.5 equiv of 4,4-(buta-1,3-diyne-1,4-diyl)bis-pyridine-*N*-oxide **8** as a template. The ^1^H NMR spectrum of a solution containing a mixture of tetra-amine **2** (1 equiv), bis-aldehyde **3** (2.2 equiv), and template **8** (0.5 equiv) was acquired approximately 1 h following its preparation. The spectrum showed sharp and well-defined proton signals (Figure S8) diagnostic of the quantitative self-assembly of the **8**⊂**1** cage inclusion complex (>90%). We characterized the encapsulation complex by a complete set of high-resolution spectra (NMR and HRMS, see SI). A ^1^H DOSY NMR experiment in a 9:1 CDCl_3_:CD_3_CN solvent mixture assigned the same diffusion constant value to the protons of the cage and the included guest, *D* = 3.10 ± 0.06 × 10^–10^ m^2^ s^–1^, evidencing their involvement in the same encapsulation complex (Figure S9). Moreover, the diffusion constant value coincided with that of the free octa-imine cage **1** in the same solvent mixture (Figure S5).

**Figure 2:**
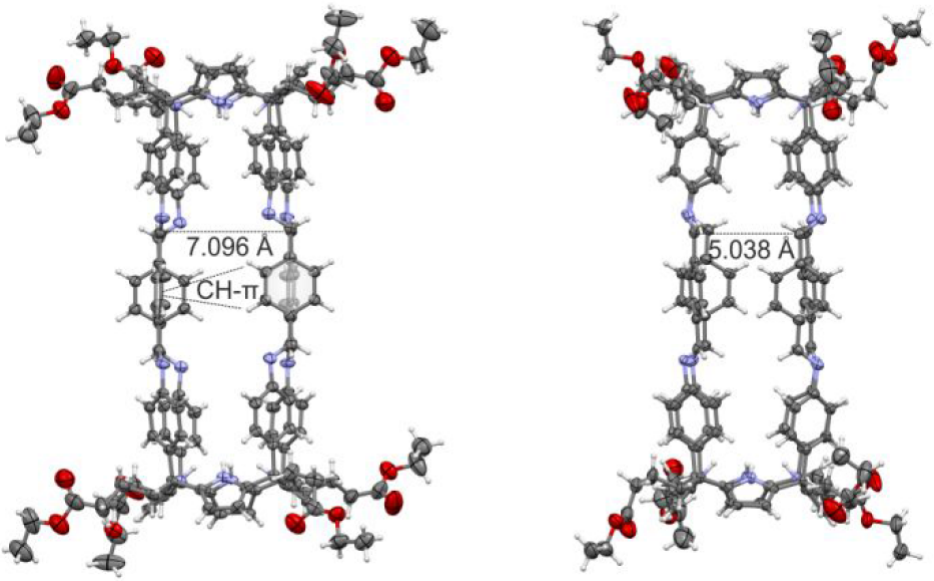
Side views of the X-ray crystal structure of the octa-imine **8**⊂**1** complex showing the two differently sized portals. C_imine_–C_imine_ distance is shown for each portal. CH−π interactions between the edge-to-face oriented central aromatic panels are depicted. Bound guest is omitted for clarity. Thermal ellipsoids for C, N, and O atoms are set at 50% probability. H atoms are shown as spheres of 0.30 Å in diameter. To see the structure with the included guest, see Figure S10 in the Supporting Information.

Single crystals suitable for X-ray diffraction grew from the chloroform solution containing the **8**⊂**1** complex (Figures 2 and S10). In the solid state, all imine bonds of the **8**⊂**1** complex showed *E*-conformation. The imine protons were arranged in pairs facing one another in the two C[4]P hemispheres of **1**. This arrangement defined two differently sized portals for cage **1**. The aromatic spacers were involved in CH-π interactions, with the two adjacent panels displaying alternate edge-to-face orientations.

### Binding Studies of the Octa-imine Cage **1** with Pyridine-*N*-oxide Derivatives

As exemplified by the solid-state structure of the **8**⊂**1** complex, receptors based on AE-C[4]P units bind pyridine-*N*-oxide derivatives with high affinity in chlorinated solvents.^30,36^

Molecular modeling studies (Scigress, MM3) showed that pyridine-*N*-oxide derivatives **4a** and **5** were good fits for the polar aromatic cavity of the octa-imine cage **1**. The structures of the 2:1 homocomplexes, (**4a**)_2_⊂**1** and (**5**)_2_⊂**1**, and the heterocomplex (**4a**·**5**)⊂**1** were energy-minimized using DFT calculations at the RI^41−43^-BP86^41^-D3BJ^44,45^/def-SV(P)^46,47^ level of theory as implemented in Turbomole 7.0.^48,49^ The results of the quantum calculations produced sensible structures for all the inclusion complexes (see Figures S68, S69, and S71). In the (**4a**·**5**)⊂**1** heterocomplex, the *para*-substituted reacting groups (azido and ethynyl) of the pyridine-*N*-oxides were located at a reasonable distance to engage in a 1,3-dipolar cycloaddition reaction (Figure S72).

#### Azido Pyridine-*N*-oxide **4a** and 4-Ethynyl pyridine-*N*-oxide 5

First, we probed the binding properties of the octa-imine cage **1** with azido pyridine-*N*-oxide guest, **4a**, by means of ^1^H NMR spectroscopic titrations (Scheme 2). Adding 1 equiv of the azido *N*-oxide **4a** to a 2 mM solution of octa-imine **1** in CDCl_3_:CD_3_CN 9:1 solvent mixture induced the appearance of two new sets of signals for the protons of cage **1**. We attributed these new sets of signals to bound **1** in two inclusion complexes of different stoichiometry. The proton signals of the free octa-imine **1** were still detected in the ^1^H NMR spectrum of the mixture (Figure S29).

**Figure 3:**
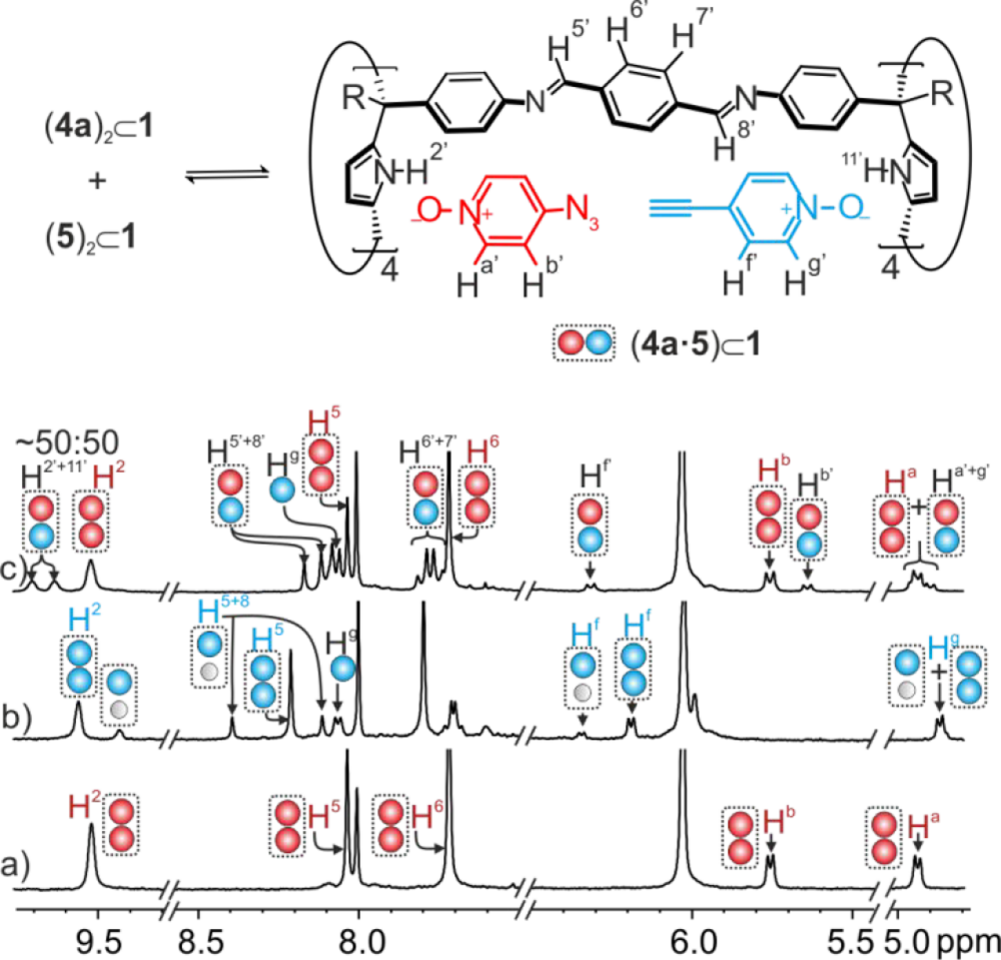
Selected regions of the ^1^H NMR spectra of (a) 1:2 mixture of **1**:**4a**; (b) 1:2 mixture of **1**:**5**; and (c) 1:2:2 mixture of **1**:**4a**:**5**. Proton assignments in blue and red correspond to 1:1 and 2:1 homocomplexes with **4a** and **5**, respectively. Primed protons in black correspond to ternary heterocomplex (**4a**·**5**)⊂**1** and protons in black to the free guest **5**. The disproportionation equilibrium of homocomplexes (**4a**)_2_⊂**1** and (**5**)_2_⊂**1** into the heterocounterpart (**4a**·**5**)⊂**1** is shown on the top.

Adding more than 1.0 equiv of **4a** provoked a noticeable increase in the intensity of one of the two new sets of signals and the disappearance of that assigned to free **1**. When 2.0 equiv of **4a** were added, the ^1^H NMR spectrum of the solution showed an exclusive set of sharp and well-defined proton signals for **1** (Figure 3a). The pyrrole NHs resonated at δ = 9.54 ppm as a broad singlet. The downfield shift experienced by the NHs was indicative of their involvement in hydrogen bonds. The spacer’s imine and aromatic protons appeared as two sharp singlets centered at δ = 8.06 and δ = 7.74 ppm, respectively. Moreover, the proton signals of **4a** resonated as two *ortho*-coupled doublets at δ = 5.77 and 4.96 ppm, (Δδ = −3.14 and −1.13 ppm, respectively). The upfield shifts experienced by the pyridine-*N*-oxide protons confirmed its inclusion in the polar C[4]P aromatic cavity defining the hemispheres of cage **1**. The chemical shift changes and the number of proton signals were consistent with the quantitative formation of the (**4a**)_2_⊂**1** complex featuring *D*_4*h*_ symmetry (see energy minimized structure in Figure S68). The exclusive observation of the proton signals of the (**4a**)_2_⊂**1** complex after adding 2.0 equiv of **4a** allowed us to estimate its binding constant as larger than 10^8^ M^–2^.^50^

Taken together, the results described above indicated that cage **1** included the azido pyridine-*N*-oxide **4a** in its polar cavity, producing two inclusion complexes of 1:1, **4a**⊂**1**, and 2:1, (**4a**)_2_⊂**1**, stoichiometry. In the 1:1 complex, **4a** is likely coincluded with one acetonitrile molecule: (**4a·CH**_**3**_**CN**)⊂**1**.

We used the integral values of selected proton signals of the three species involving cage **1** to determine their concentrations’ ratios throughout the ^1^H NMR titration. Combining the experimentally determined concentrations’ ratios with the theoretical speciation profiles produced by the HySS2009 software, we estimated the stepwise macroscopic binding constant values as K[(CH_3_CN)_2_⊂**1** + **4a** ⇌ (**4a·**CH_3_CN)⊂**1**] = 2.0 × 10^4^ M^–1^; K[(**4a·**CH_3_CN)⊂**1** + **4a** ⇌ (**4a**)_**2**_⊂**1**] = 3.1 × 10^4^ M^–1^. This result assigned a reduced positive cooperativity to the second binding process.

In the same line, the observation of a single binding isotherm in the isothermal titration calorimetry (ITC) experiments (Figure S30) confirmed that the binding process of including two copies of **4a** in the polar cavity of **1** did not feature significant levels of cooperativity, as anticipated from the ^1^H NMR titration results (α = (2 × *K*_1:1⇌2:1_)/(*K*_1:1_/2) = 6).^51,52^

We performed analogous ^1^H NMR and ITC titration experiments with 4-ethynyl pyridine-*N*-oxide **5**. As before, we estimated the values for the stepwise binding constants, K[(CH_3_CN)_2_⊂**1** + **5** ⇌ (**5·**CH_3_CN)⊂**1**] = 3.2 × 10^4^ M^–1^; K[(**5·**CH_3_CN)⊂**1** + **5** ⇌ (**5**)_**2**_⊂**1**] = 1.2 × 10^3^ M^–1^, by comparing experimental and theoretical speciation profiles of the ^1^H NMR titration experiments using HySS2009 software (vide supra). From them, we derived that the binding process showed a small negative cooperativity factor, α = (2 × *K*_1:1⇌2:1_)/(*K*_1:1_/2) = 0.15. The ITC experiments conducted to accurately characterize the binding process of **1** and **5** in a CHCl_3_:CH_3_CN 9:1 solvent mixture yielded a single sigmoidal binding isotherm (Figure S35). The inflection point was centered at a **5**/**1** molar ratio of 2. The experimental data fit provided an average value for the binding constant of 4.5 × 10^4^ M^–1^ for the two binding events. Most likely, the negative cooperativity calculated from the NMR titration data is too subtle to be detected by ITC experiments.

#### Pairwise Inclusion of Pyridine-*N*-Oxides **4a** and **5** in Cage **1**

We explored the formation of the ternary heterocomplex (**4a**·**5**)⊂**1** by adding 2 equiv of each pyridine-*N*-oxide, **4a** and **5**, to a 2 mM solution of the octa-imine cage **1** in a 9:1 CDCl_3_:CD_3_CN solvent mixture. The ^1^H NMR spectrum of the solution evidenced, in the downfield region, the diagnostic signals of the imine and pyrrolic NH protons of the (**4a**)_2_⊂**1** complex. However, we did not detect the corresponding signals for the (**5**)_2_⊂**1** complex and the 1:1 complexes (**4a**·**CH**_**3**_**CN**)⊂**1** and (**5·CH**_**3**_**CN**)⊂**1**. We observed a new set of split NH and imine signals that we assigned to the protons of the (**4a**·**5**)⊂**1** ternary heterocomplex featuring two chemically nonequivalent hemispheres (Figure 3). Using the integral values of the NH signals of the two detected cage complexes, we estimated their concentrations to be [(**4a**)_2_⊂**1**] ∼ 0.9 mM and [(**4a·5**)⊂**1**] ∼ 0.8 mM. As expected, we also observed signals corresponding to the protons of the excess free *N*-oxides **4a** and **5**. Using the previously estimated constant values for the 1:1 and 2:1 homoinclusion complexes of **1** with **4a** and **5** and the HySS2009 software (vide supra), we simulated the theoretical speciation profile of the mixture. The theoretical simulation agreed with the experimental speciation profile of the observed inclusion complexes when a binding constant value of β[(CH_3_CN)_2_⊂**1** + **4a** + **5** ⇌ (**4a**·**5**)⊂1] = 2.8 × 10^8^ M^–2^ was assigned to the ternary heteroinclusion complex (see Figures S36–S38).

In summary, the ternary complexes (**4a**)_2_⊂**1** and (**4a**·**5**)⊂**1** are almost 1 order of magnitude more stable than the (**5**)_2_⊂**1** counterpart (Table 1). This produced the exclusive observation of the two former complexes and the lack of detection of the latter in the presence of an excess of the *N*-oxides. It also resulted in an equilibrium constant for the disproportionation process of homocomplexes into the heterocounterpart of *K*_dispro_= 3. The kinetics of the formation of the inclusion complexes of the pyridine-*N*-oxides and cage **1** are fast on the human time scale (i.e., seconds/mins). However, their binding processes showed slow kinetic on the ^1^H chemical shift time scale (i.e., separate signals for free and bound binding partners, Figure 3).

**Table 1:** Stepwise and Overall Binding Constant Values (*K*_1:1,_ M^–1^; *K*_1:1⇌2:1_,M^–1^; β_2:1_ = *K*_1:1_ × *K*_1:1⇌2:1_, M^–2^) Determined for the 1:1 and 2:1 Homo- and Heteroternary Complexes of the Octa-imine Cage **1** with the Pyridine-*N*-oxide Derivatives **4a**, **4b**, **4c**, and **5** as Guests^a^

host	1st guest	*K*_1:1_ (M^–1^)^b^	2nd guest	*K*_1:1⇌2:1_ (M^–1^)^c^	β_2:1_ (M^–2^)^d^
**1**	**4a**	2.0 × 10^4^	**4a**	3.1 × 10^4^	6.2 × 10^8^
**1**	**5**	3.2 × 10^4^	**5**	1.3 × 10^3^	4.0 × 10^7^
**1**	**4b**	4.0 × 10^5^	**4b**	4.0 × 10^3^	1.6 × 10^9^
**1**	**4c**	4.0 × 10^5^	**4c**	1.0 × 10^4^	4.0 × 10^9^
**1**	**4a**	2.0 × 10^4^	**5**	1.4 × 10^4^	2.8 × 10^8^
**1**	**5**	3.2 × 10^4^	**4a**	8.8 × 10^3^	2.8 × 10^8^
**1**	**4b**	4.0 × 10^5^	**5**	5.0 × 10^2^	2.0 × 10^8^
**1**	**5**	3.2 × 10^4^	**4b**	6.3 × 10^3^	2.0 × 10^8^
**1**	**4c**	4.0 × 10^5^	**5**	3.3 × 10^2^	1.3 × 10^8^
**1**	**5**	3.2 × 10^4^	**4c**	4.1 × 10^3^	1.3 × 10^8^

a Binding constant values were determined by fine-tuning theoretical speciation profiles produced using HySS2009 software to the experimentally measured concentration ratio of the different species detected in the ^1^H NMR spectra registered during spectroscopic titrations. For the formation of 1:1 and 2:1 homocomplexes, we use a binding model considering two reagents, (CH_3_CN)_2_⊂**1**, and **G = 1st Guest** = **2nd Guest** forming two species (**G**·CH_3_CN)⊂**1**, and (**G**·**G**)⊂**1**. For the formation of the 2:1 heterocomplexes, we use a binding model considering three reagents, (CH_3_CN)_2_⊂**1**, **G1** = **1st Guest**, and **G2** = **2nd Guest** forming five species: (**G1**·CH_3_CN)⊂**1**, (**G2**·CH_3_CN)⊂**1**, (**G1**·**G1**)⊂**1,** (**G2·G2**)⊂**1**, and (**G1**·**G2**)⊂**1**. In the latter binding model, the constant values of the 1:1 and 2:1 homocomplexes were fixed to those determined with the former.

b The *K*_1:1_ constants correspond to the binding equilibrium producing the 1:1 complex from the host solvate: (CH_3_CN)_2_⊂**1** + **1st Guest** ⇌ (**1st Guest** ·CH_3_CN)⊂**1**.

c The *K*_1:1⇌2:1_ constants refer to the stepwise equilibrium for the formation of the 2:1 complexes from the 1:1 counterparts: (**1st Guest** · CH_3_CN)⊂**1** + **2nd Guest** ⇌ (**1st Guest** · **2nd Guest**)⊂**1**.

d β_2:1_ = *K*_1:1_ × *K*_1:1⇌2:1_, stands for the overall binding constant of the equilibrium producing the 2:1 complexes from the host solvate: (CH_3_CN)_2_⊂**1** + **1st Guest** + **2nd Guest** ⇌ (**1st Guest** · **2nd Guest**)⊂**1**.

### Monitoring the 1,3-Dipolar Cycloaddition Reaction of **4a** with **5** Included in **1**

We used ^1^H NMR spectroscopy to analyze, at different time intervals and during 2 weeks, the solution containing the almost equimolar mixture of cage complexes (**4a·5**)⊂**1** and (**4a**)_2_⊂**1**. We did not detect noticeable changes in the acquired ^1^H NMR spectra during this time. This result indicated that the cycloaddition reaction between **4a** and **5** was not accelerated to a detectable extent by inclusion in the octa-imine cage **1** for 2 weeks under these experimental conditions.

Intending to have available the ^1^H NMR spectrum of the **6a**⊂**1** complex, corresponding to the 1,4-disubstituted triazole isomer of the cycloaddition reaction of **4a** with **5** included in cage **1**, we performed a solid–liquid extraction experiment of **6a** using a 2 mM solution cage **1** in a 9:1 CDCl_3_:CD_3_CN solvent mixture.^53^ After sonication and filtration, the ^1^H NMR spectrum of the obtained solution did not show any sharp signal that could be assigned to the protons of bound **6a**. Moreover, some proton signals of cage **1** broaden beyond detection (Figure S39).

**Figure 4:**
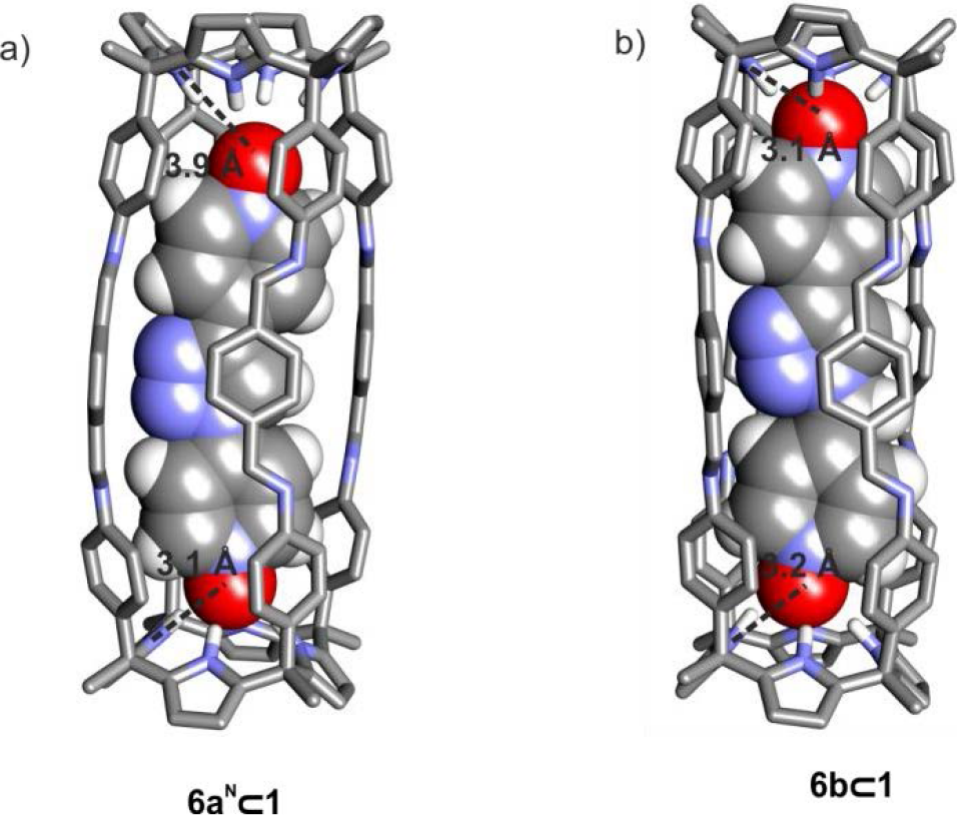
Energy minimized structure (DFT) of complexes (a) **6a**^**N**^⊂**1** and (b) **6b**⊂**1**. The N–O···N–H hydrogen-bonding distances of its two hemispheres are depicted.

We hypothesized that the length of the bis-*N*-oxide **6a** was too short to span the gap between the two C[4]P cage’s hemispheres of **1** and produce a ditopic interaction with optimal hydrogen-bonding distances. To support this hypothesis, we optimized the structure of the two possible isomers of the **6a**⊂**1** complex at the RI^41−43^-BP86^41^-D3BJ^44,45^/def-SV(P)^46,47^ level of theory using Turbomole v7.0 (**6a**^**C**^⊂**1** and **6a**^**N**^⊂**1** in Figure S72).^48,49^ The computed energies were similar for both isomers (Δ*E* < 0.2 kcal·mol^–1^). For any of the two isomers, the energy-minimized structure showed that the N–O···N–H hydrogen bonding distances of its two hemispheres were significantly different (3.1 and 3.9 Å, Figure 4a).

This result suggested that reaching an energetically favorable transition state (TS) for the 1,3-dipolar cycloaddition reaction between **4a** and **5** included in **1** would require that at least one of the pyridine-*N*-oxides knobs, holding the reacting groups, moves toward the other. In doing so, the *N*-oxide knob will be displaced from its optimal binding geometry. The distance of the hydrogen bonding, π–π, and CH−π interactions of the *N*-oxide with the AE-C[4]P unit will be elongated, producing a concomitant increase in the energy of the complex that will be translated to that of the TS. We consider that these geometric requirements might serve to explain why the cycloaddition between **4a** and **5** is not significantly accelerated when included in **1**.

This reasoning also finds that the reduced flexibility of cage **1** cannot cope with the inadequate complementarity of sizes (i.e., length) between the cage’s cavity and guest **6a** to produce an optimal ditopic interaction nor with approaching the included reactants **4a** and **5** without disrupting their intermolecular interactions with the container.

### Study of the 1,3-Dipolar Cycloaddition Reaction of **4b** with **5** Included in **1**

We considered that 4-azido(methyl) pyridine-*N*-oxide, **4b**, featuring a methylene unit between the azido group and the *para*-carbon of the pyridine-*N*-oxide, should locate the two reacting groups in the heterocomplex (**4b·5**)⊂**1** in closer spatial proximity compared to the (**4a·5**)⊂**1** analog described in the previous section. For the same token, the bis-*N*-oxide **6b**, the 1,4-disubstituted triazole isomer of the cycloaddition reaction of **4b** and **5,** should be a superior fit for the dimensions of the cavity of cage **1** than the homologous **6a** (Figure 4b). First, we experimentally investigated the inclusion of **6b** in cage **1**. The ^1^H NMR spectrum of an equimolar mixture of **6b** and **1** produced sharp and well-defined proton signals diagnostic of the quantitative formation of the **6b**⊂**1** complex, featuring *C*_4_ symmetry (Figures S45–S47).^54^

With this information, we evaluated the pairwise inclusion of *N*-oxides **4b** and **5** in the octa-imine cage **1** and their putative cycloaddition reaction once included. We prepared a 2 mM solution of **1** and added 1 equiv of the new dipole **4b** and 2 equiv of the previously used dipolarophile **5**. Owing to the larger thermodynamic stability of the (**4b**)_2_⊂**1** and aiming to increase the concentration of the heteroternary complex (**4b·5**)⊂**1**, we used a 1:1:2 molar ratio of **1**, **4b**, and **5**, instead of the 1:2:2 used for the assembly of the (**4a·5**)⊂**1**.^55^

**Figure 5:**
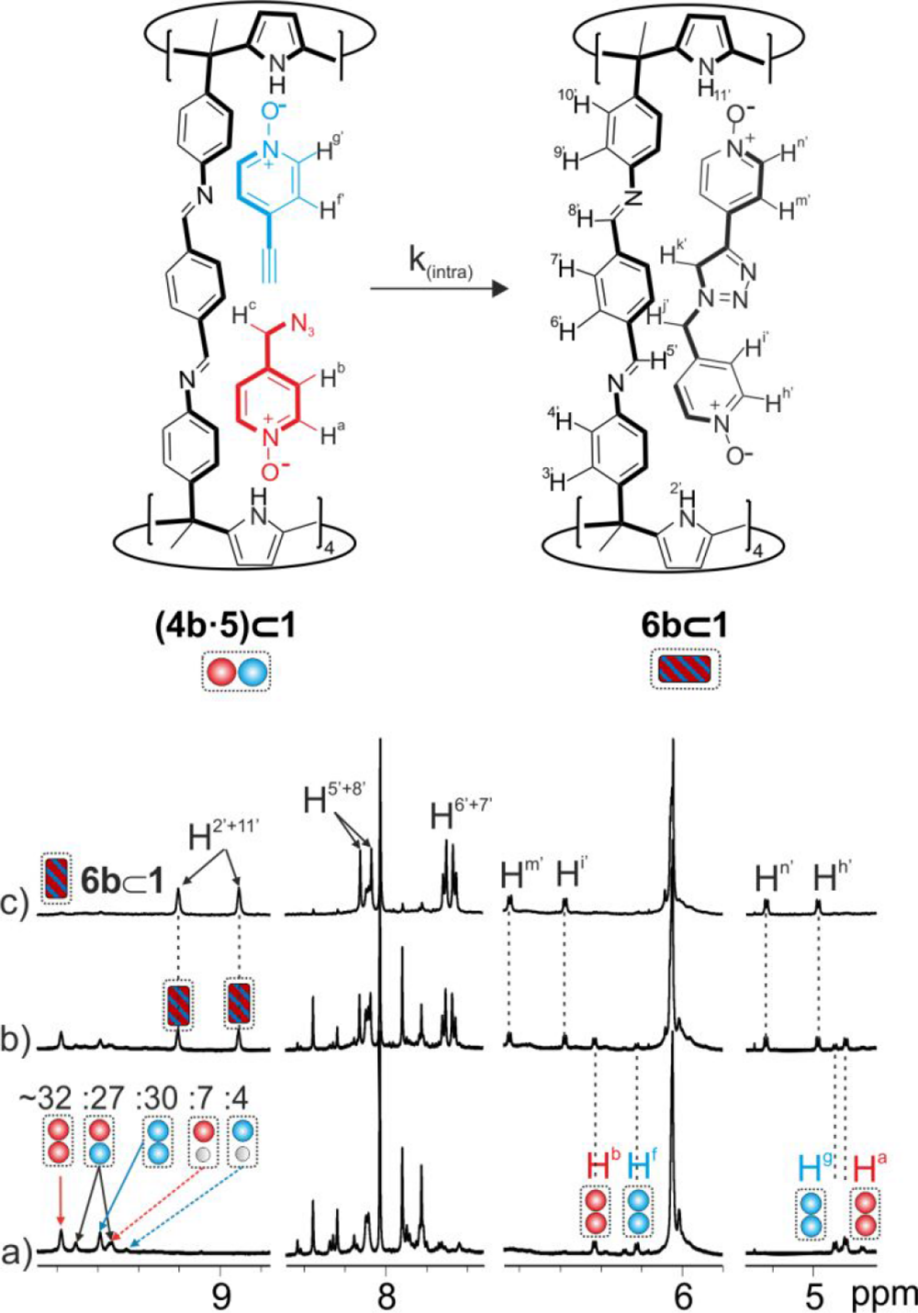
Selected regions of the ^1^H NMR spectra corresponding to the kinetic study of the formation of the **6b**⊂**1** complex starting from a 1:1:2 mixture of **1**:**4b**:**5**. (a) 1:1:2 mixture of **1**:**4b**:**5** (*t* = 0 h); (b) 1:1:2 mixture of **1**:**4b**:**5** (*t* = 8 h); and (c) 1:1:2 mixture of **1**:**4b**:**5** (*t* = 48 h). The percentage of bound species at *t* = 0 h is indicated in spectrum (a). Proton assignments in blue and red in spectrum (a) correspond to complexes with **5** and **4b**, respectively. Primed protons in black correspond to the **6b**⊂**1** complex. The scheme of the 1,3-dipolar cycloaddition reaction between **4b** and **5** inside the cage yielded complex **6b**⊂**1** is shown on the top.

The ^1^H NMR spectrum of the solution acquired following the preparation of the mixture showed the homoternary complexes (**5**)_2_⊂**1** and (**4b**)_2_⊂**1** as the major species (Figures 5a and S42). We also detected, to a much-reduced extent, the proton signals of the 1:1 complexes: (**5·CH**_**3**_**CN**)⊂**1** and (**4b·CH**_**3**_**CN**)⊂**1**. We attributed an additional set of signals of cage **1** to the protons in the heteroternary (**4b·5**)⊂**1** complex. Using integral values of selected proton signals, we quantified its concentration as [(**4b·5**)⊂**1**] ∼ 0.54 mM. Finally, we also identified proton signals of low intensity for the free cage **1**, and the free *N*-oxides **4b** and **5**. The theoretical simulation (HySS2009) of the experimentally observed speciation profile assigned a stability constant value of β[(CH_3_CN)_2_⊂**1** + **4b** + **5** ⇌ (**4b**·**5**)⊂**1**] = 2.0 × 10^8^ M^–2^ to the heteroternary complex (see Figure S44).

We analyzed the changes in the solution with time using ^1^H NMR spectroscopy. After 30 min, we observed the emergence of a new set of proton signals of cage **1**. This was especially clear in the upfield spectrum region where two new pyrrole NHs signals centered at δ = 9.2 and 8.9 ppm appeared. The intensity of the latest set of signals increased with time at the expense of those of the homocomplexes (**4b**)_2_⊂**1** and (**5**)_2_⊂**1**. Moreover, the intensity of the proton signals of the heterocomplex (**4b·5**)⊂**1** remained almost constant in the initial phase of the monitoring process (∼0.54 mM). After 48 h, we exclusively observed the set of signals of the newly formed species (Figure 5c), which coincided with those of the previously prepared (**6b**)⊂**1** complex. Taken in concert, these results indicated that the 1,3-dipolar cycloaddition between **4b** and **5** occurred when included in cage **1**. Furthermore, it only produced the 1,4-triazole isomer **6b**.^56^ We performed analogous kinetic experiments using different molar ratios of **1**:**4b**:**5** (1:1:1 and 1:5:5), producing the ternary complex (**4b·5**)⊂**1** in lower concentrations (0.32 mM and 0.1 mM, respectively). We used the changes in the integral values of selected proton signals of the **6b**⊂**1** complex to determine its changes in concentration with time. First, we used the initial rates method to unequivocally assign to the included 1,3-dipolar cycloaddition reaction between **4b** and **4a** a first-order rate law concerning exclusively the concentration of the heteroternary complex, υ_intra_ini_ = d[(**6b**⊂**1**]**/**d*t***=***k*_intra_ [(**4b·5**)⊂**1**]. We derived an average value for the reaction’s rate constant, *k*_(6b-intra)_ = 5 (±3) × 10^–5^ s^–1^, by linear fitting the kinetic data at different concentrations.^57^

**Figure 6:**
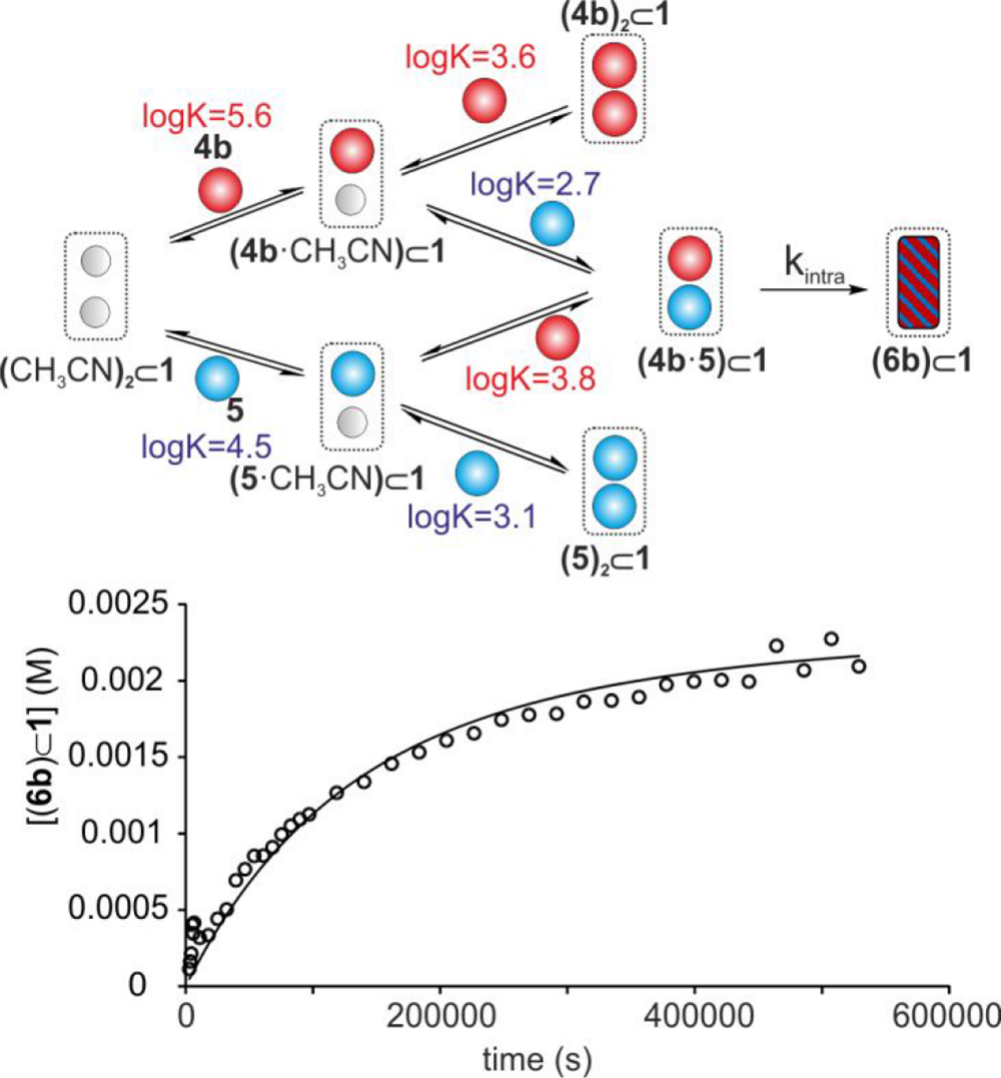
(Top) Theoretical kinetic model used for the nonlinear mathematical analysis of the experimental data. (Bottom) Changes in the concentration of the **6b**⊂**1** complex (empty dots) with time starting from a 1:1:1 mixture of **1**:**4b**:**5** in CDCl_3_:CD_3_CN 9:1. The solid line represents the fit of the kinetic data to the theoretical model using the parameter estimation module of COPASI Software Version 4.25. All binding equilibria’s *k*_on_/*k*_off_ ratios were manually fixed based on the determined binding constants. *k*_(intra)_ was the only variable parameter used for the fit.

Second, we constructed an elaborated kinetic model considering all the binding equilibria in the solution and the irreversible 1,3-dipolar cycloaddition reaction of **4b** and **5** occurring in **1** (Figure 6). The contribution from the uncatalyzed reaction (bulk) was not considered in the model (i.e., negligible at this concentration). Neither did we consider the dissociation of the cycloaddition product complex to give free **6b** in solution (K[(CH_3_CN)_2_⊂**1** + **6b** ⇌ (**6b**·CH_3_CN)⊂**1**] > 10^8^ M^–1^). This model will help derive rate constant values following the included cycloaddition reaction to completion and by a best-fit computer simulation (COPASI) of the kinetics over the whole reaction curve. We assumed that the rate constants of the equilibrium steps were faster than that of the irreversible included reaction.^58^ We manually fixed the *k*_on_/*k*_off_ ratios of the binding equilibria to the determined association constants values of the formed complexes, *K* = *k*_on_/*k*_off_.^59^

The experimental kinetic data obtained for the complete formation of the **6b**⊂**1** complex at different molar ratios of **1**:**4b**:**5** showed an excellent fit to the elaborated kinetic model, producing an average rate constant for the included cycloaddition reaction of *k*_(6b-__intra)_ = 7 (±5) × 10^–5^ s^–1^ (Figures S55–S57). This value coincided with the one previously derived using the initial rates method.

In assessing the acceleration factor caused by including the cycloaddition reaction between 4-ethynyl pyridine-*N*-oxide **5** and 4-azido(methyl) pyridine-*N*-oxide **4b** in the octa-imine **1**, we determined the reaction’s rate constant in the bulk solution. To this end, we reacted the substrates at 25 mM concentration in a 9:1 CDCl_3_:CD_3_CN solvent mixture at 298 K. The high concentration used to carry out the bulk reaction and its low reaction rate required HPLC to monitor its progress. We used 4-methyl pyridine-*N*-oxide as the internal standard (i.s.) (Figures S59–S65 and Table S1).^60^ Under these conditions, the cycloaddition reaction produced a mixture of the 1,4-, **6b**, and 1,5-, **7b**, triazoles in approximately 2:1 molar ratio (Scheme 2). We fit the experimental data to a theoretical kinetic model, which considered the irreversible bimolecular reaction between **5** and **4b** to produce isomer **6b** using COPASI. The fit returned a value for *k*_(6b-bulk)_ of 5.6 × 10^–8^ M^–1^ s^–1^.^61^ Considering this result, at 2 mM concentration of reactants, the 1,3-dipolar cycloaddition reaction between **5** and **4b** in the bulk solution will require ∼317 years to produce a 1 mM concentration of **6b**. For this reason, we considered the amount of **6b** produced by the background reaction negligible and neglected its inclusion in the elaborated kinetic binding model. The analysis of the kinetics of the cycloaddition reaction between **4a** and **5** in the bulk solution yielded a rate constant *k*_(6a-bulk)_ of similar magnitude (see Table 2).

**Table 2:** Rate Constant Values for the 1,3-Dipolar Cycloaddition Reactions in Bulk (*k*_bulk_, M^–1^ s^–1^) and the Octa-imine Cage Cavity (*k*_intra_, s^–1^)^a^

guest pair	product	*k*_bulk_ (M^–1^ s^–1^)^b^	*k*_intra_ (s^–1^)^c^	EM (M)^d^
(**4a·5**)	**6a**	5.1 × 10^–8^	n.d.	n.d.
(**4b·5**)	**6b**	5.6 × 10^–8^	5.0 × 10^–5^	∼10^3^
(**4c·5**)	**6c**	3.6 × 10^–8^	8.1 × 10^–5^	∼10^3^

a Acceleration factors reported as effective molarities (EM, M) are also listed.

b Determined by best-computer fit of the kinetic experimental data using COPASI and a kinetic theoretical model for an irreversible first-order bimolecular reaction. Experimental data were obtained from the changes in concentration of the product, **6a**–**c**, with time for the reactions between **4a**–**c** with **5** at 298 K. The initial concentration of reactants was 25 mM in a 9:1 CDCl_3_:CD_3_CN solvent mixture. We used HPLC and calibration curves to accurately determine the concentrations of **6a**–**c**.

c Determined by best-computer fit of the kinetic experimental data using COPASI and an elaborated kinetic model considering six binding equilibria in the solution, producing the 1:1 and 2:1 homocomplexes, as well as the ternary (Michaelis) complex, and the irreversible pseudounimolecular reaction of the ternary complex producing the product bound in the octa-imine cage. Experimental kinetic data were derived from the concentration changes with the time of the complex that resulted from including the reaction product in the container cage. We used ^1^H NMR spectroscopy to monitor the reaction starting from a mM equimolar mixture of **1**, **4**, and **5** dissolved in a CDCl_3_:CD_3_CN 9:1 solvent mixture.

d EM = *k*_intra_/*k*_bulk_; n.d. not determined. We did not detect noticeable changes in the ^1^H NMR spectra acquired during 2 weeks for the 1:1:1 mixture of **1**, **4a**, and **5**.

We assessed the rate acceleration factor provoked by the inclusion of the cycloaddition reaction between **5** and **4b** in the cavity of cage **1**, determining its effective molarity, EM = *k*_(6b-intra)_/*k*_(6b-bulk)_ = 1.0 (±0.2) × 10^3^ M. This EM value can be assigned to an entropy factor of −13.7 e.u. or an energy difference of 4.1 kcal·mol^–1^ in favor to the transition state (TS) of the cycloaddition reaction between **5** and **4b** included in **1** compared to that in the bulk solution. This result agreed with the hypothesis of Page and Jenks, stating that enzymes might carry out a significant fraction of their extraordinary rate accelerations, compensating reactions’ entropy costs by substrates’ binding and confinement in the active site, without the need to invoke other concepts or the enthalpic stabilization of the TS.^3^ A reaction’s acceleration factor was also assessed from the ratio of initial rates, *v*_0(6b-intra)_/*v*_0(6b-bulk)_, in the presence and absence of cage **1**, respectively. We calculated the hypothetical initial reaction rate of the reaction in bulk, *v*_0(6b-bulk)_, at 2 mM concentrations of **4b** and **5** using the determined value of *k*_(6b-bulk)_ (see Table S1). We took the value of *v*_0(6b-intra)_ from the reaction performed using 2 mM concentrations of **5**, **4b**, and **1**. The ratio of initial rate values returned an acceleration factor of 10^4^, which cannot be converted into an energy difference of the TS but provided a physical magnitude of the effect caused by the reaction inclusion on its rate.

Unfortunately, as with other reactions accelerated by inclusion in molecular containers, the tight binding of the product, bis-*N*-oxide **6b**, to cage **1** inhibited turnover.

We also performed a series of control experiments (see Supporting Information section 5) to verify that the coinclusion of **4b** and **5** in the cavity of cage **1** unequivocally caused the acceleration of their dipolar cycloaddition. For example, the 1,3-cycloaddition reaction of azido/ethynyl guest molecules that do not simultaneously fit into the cage’s cavity do not form the ternary heterocomplex (e.g., azido(methyl) pyridine-*N*-oxide **4b** and 4-(4-ethynylphenyl) pyridine-*N*-oxide **10**), is not accelerated to a measurable extent (see Supporting Information, Table S2).

### Study of the 1,3-Dipolar Cycloaddition Reaction of **4c** with **5** Included in **1**

We also studied the cycloaddition reaction between azido(ethyl) pyridine-*N*-oxide **4c,** a dipole featuring two methylene units between the azido group and the *para*-carbon of the pyridine-*N*-oxide, and the same dipolarophile **5**. The regioselective reaction produced exclusively the 1,4-triazole isomer, **6c**, in the **6c**⊂**1** complex. We assessed the EM reaction’s acceleration factor as ∼10^3^ M. We did not expect that the cycloaddition reactions of **4b** with **5** and of **4c** with **5**, included in **1**, experienced EM acceleration factors of the same magnitude. The TS of the reaction between **4c** and **5,** when included in **1,** required locking an additional single bond rotation compared to that of **4b** with **5,** which should be associated with an additional entropy cost and the corresponding increase of the TS barrier. However, from the obtained result, we concluded that **4c** did not experience low-energy conformational changes within the heteroternary (**4c·5**)⊂**1** complex, contributing negatively to the reaction’s acceleration.

### Comparison of the Obtained Results with Previous Examples of Accelerating 1,3-Dipolar Cycloadditions by Inclusion in Molecular Containers

What is unique about octa-imine **1**, is its sizable polar cavity equipped with two convergent and endohedrally functionalized binding sites, AE-C[4]P. These characteristics allowed the tight-binding and pairwise inclusion of two pyridine-*N*-oxides in a well-defined and fixed orientation. The arrangement of some of the included *N*-oxides provoked a suitable alignment of their *para*-substituents in a chemical reaction. We selected the 1,3-dipolar Huisgen cycloaddition reaction as a benchmark to investigate the acceleration caused by including the reacting groups in the cavity of **1**. As mentioned in the introduction, we were not the first to examine the acceleration effect resulting from including a 1,3-dipolar cycloaddition of azide and alkyne in a molecular container. For example, Rebek and Jia studied the inclusion of the cycloaddition reaction of phenyl azide with phenylacetylene in a resorcin[4]arene dimer.^27^ The reaction was regioselective, and an EM acceleration factor of 1.2 × 10^2^ M was later determined.^25^ The dimer had an aromatic cavity suitable for coincluding the two reactants. However, it lacked inner functional groups to control the relative positioning of the substrates, the reacting groups’ orientation, and the substrates’ binding affinity. As is the case here, the direct observation of the ternary-hetero complex simplified the kinetic data analysis.

We assigned the increase of an order of magnitude of the EM (1.0 × 10^3^ M) measured for related dipolar cycloaddition reaction included in cage **1** to the tighter binding of the pyridine-*N*-oxide knobs of the substrates to the polar AE-C[4]P defining the hemispheres of the container. The *N*-oxides are located in fixed positions in the container’s cavity, owing to the formation of four convergent hydrogen bonds between their oxygen atoms and the pyrrole NHs. Their relative motions within the heteroternary complex are reduced, and the reacting groups are adequately oriented. The fixed position of the *N*-oxide substrates is evidenced by the lack of reactivity in the cycloaddition reaction of **4a** and **5** included in **1**.

Mock and co-workers^24^ used CB[6] as a molecular container to accelerate the cycloaddition reaction of azido and alkynyl *N*-*tert*-butylated substrates in water solution. The reaction was regioselective, but the ternary complex was not experimentally observed. This raised the problem of “non-productive binding”, that is, the existence of a heteroternary complex in which the second substrate had the reactive group in the exterior of CB[6], evidencing that it was not suitable to unequivocally control the reacting groups’ orientation as cage **1** does. All these considerations complicated the kinetic analysis and led to some internal data inconsistency. For this reason, the authors referred to the reported “acceleration factor” as an approximation. As for the case of Rebek and Jia, an EM acceleration factor was also estimated a few years later by Mandolini and co-workers to be 1.6 × 10^4^ M.^25^ We consider that the Mock example represents a particular case of reaction acceleration induced by inclusion in a container. The substrates are charged, not neutral. Hence, the container neutralizes the repulsive Coulombic interaction occurring in the bulk. Due to its limited size, the container does not include the reactants, only the reacting groups. In short, the results presented here constitute the most significant acceleration reported for a bimolecular reaction included in a molecular container by directly detecting the ternary complex.

## Conclusions

We self-assembled and characterized a [4 + 2] octa-imine calix[4]pyrrole cage in a CDCl_3_:CD_3_CN 9:1 solvent mixture. Adding 0.5% of acetic acid or a bis-pyridine-*N*-oxide template molecule significantly improved the yield of the self-assembly reaction (>90%). Octa-imine cage **1** formed thermodynamically and kinetically stable 1:1 and 2:1 homo- and heterocomplexes with pyridine-*N*-oxide guests featuring an azido (**4a**, **4b, 4c**) or ethynyl substituent (**5**) in the *para-* position. Octa-imine cage **1** was found to accelerate the included 1,3-dipolar cycloaddition reactions between 4-azido(alkyl) pyridine-*N*-oxides, **4b** and **4c**, with 4-ethynyl pyridine-*N*-oxide **5**. The ternary Michaelis inclusion complexes, (**4b/4c·5**)⊂**1**, were detected in solution. The calculated EMs (∼10^3^ M) of the reactions included in octa-imine **1** are one figure larger than the one determined for an analogous reaction in Rebek’s resorcinarene dimer. We attributed our results to the tighter binding and fixed (and well-oriented) position of the reacting substrates within the polar container, reducing the entropy cost needed to achieve the TS.

Surprisingly, the included 1,3-cycloaddition of 4-azido pyridine-*N*-oxide **4a** and 4-ethynyl pyridine-*N*-oxide **5** was not accelerated to a measurable extent under analogous conditions. Nevertheless, the (**4a·5**)⊂**1** inclusion complex was present in solution. We hypothesized that the guests’ sizes (i.e., length) are inadequate for the productive cycloaddition reaction inside octa-imine cage **1**. The fixed position of the included guests and the reduced flexibility of container **1** may not be able to handle the geometric requirements needed to achieve the TS of the included reaction. Specifically, the necessary elongation of N–O···N–H hydrogen bonds for at least one guest increases the energy barrier for achieving the TS.

In brief, including bimolecular 1,3-cycloaddition Huisgen reactions in cage **1** imposes geometric and steric constraints on their transition state. This explains the observed accelerations and reactions’ regioselectivity and the measured lack of acceleration in one of the included reactions.^62^ Currently, we are investigating the properties of **1** and its more giant analogs for the acceleration of other included bimolecular reactions. We hope to communicate our results in due time.

## Data Availability

All dataset collection of computational results of this manuscript is available in the ioChem-BD repository and can be accessed through this link http://dx.doi.org/10.19061/iochem-bd-1-358 CCDC: 2393702.

## Supporting Information Available

General information and methods, synthetic procedures, characterization of new compounds, ^1^H NMR titrations, ITC experiments, fit of the experimental data to the kinetic model, and DFT calculations (PDF)
Structure of octa-imine cage **1** complex with bis-pyridine-*N*-oxide guest **8** (CIF)

## Supplementary Material

au4c01118_si_001.pdf

au4c01118_si_002.cif

## Abbreviations

EM: effective molarity
AE-C[4]P: aryl-extended calix[4]pyrrole
DA: Diels–Alder
ITC: isothermal titration calorimetry
TS: transition state
