# Supplementary material for: Acceleration and Selectivity of 1,3-Dipolar Cycloaddition Reactions Included in a Polar [4 + 2] Octa-imine Bis-calix[4]pyrrole Cage
Source: JACS Au. 2025 Jan 23;5(2):902–12. doi: 10.1021/jacsau.4c01118 (PMC11862939; doi:10.1021/jacsau.4c01118)
Supplement: Supplementary file 1 — au4c01118_si_001.pdf [file au4c01118_si_001.pdf]

Supporting Information

Acceleration and selectivity of 1,3-dipolar cycloaddition reactions included in a polar [4 +2] octa-imine bis-calix[4]pyrrole cage

Yifan Li,[a,b] Chiara F. M. Mirabella,[a] Gemma Aragay,[a] Pablo Ballester[a,c]*

[a] Institute of Chemical Research of Catalonia (ICIQ), The Barcelona Institute of Science and Technology (BIST), Avgda. Països Catalans, 16, 43007 Tarragona, Spain

[b] Universitat Rovira i Virgili, Departament de Química Analítica i Química Orgànica, c/Marcel·lí Domingo,1, 43007 Tarragona, Spain

[c] ICREA, Passeig Lluís Companys, 23, 08010 Barcelona, Spain

Table of contents

1. General information and instruments S3
2. Synthesis and characterization data S4
   2.1. Synthesis of octa-imine cage **1**. S4
   2.2. Synthesis of pyridyl *N*-oxide derivatives S11
3. Binding studies and characterization of octa-imine cage **1** with monotopic and ditopic pyridine *N*-oxide derivatives in $CDCl_3$: CD_3CN 9:1 mixture. S20
   3.1. Binding studies of 4-azido pyridine *N*-oxide **4a** with **1**. S20
   3.2. Binding studies of 4-azidomethyl pyridine *N*-oxide **4b** with **1** S22
   3.3. Binding studies of 4-azidoethyl pyridine *N*-oxide **4c** with **1**. S24
   3.4. Binding studies of 4-ethynyl pyridine *N*-oxide **5** with **1**. S25
   3.5. Pair-wise inclusion of pyridine *N*-oxides **4a** and **5** in **1**. S27
   3.6. Pair-wise inclusion of pyridine *N*-oxides **4b** and **5** with in **1**. S30
   3.7. Pair-wise inclusion of pyridine *N*-oxides **4c** and **5** in **1**. S34
4. Kinetic characterization of the cycloaddition reaction S38
   4.1. Cycloaddition reactions of **4b** and **4c** with **5** in octa-imine cage **1** S38
   4.2. Cycloaddition reactions of **4a**, **4b**, and **4c** with **5** in the bulk. S40
5. Control experiments S44
6. DFT Calculations S46
7. References S50

1. General information and instruments

Reagents were purchased from commercial suppliers and used without further purification. All reactions were performed under Ar atmosphere unless otherwise specified. All solvents were of HPLC grade quality, commercially obtained, and used without further purification except pyrrole, which was distilled and freshly used. Anhydrous solvents were obtained from a solvent purification system SPS-400-6 from Innovative Technologies.

Routine ^{1}H NMR and $^{13}C\{^{1}H\}$NMR spectra were recorded on a Bruker Avance 400 (400 MHz for ^{1}H NMR and 100 MHz for ^{13}C NMR), Bruker Avance 500 (500 MHz for ^{1}H NMR and 125 MHz for ^{13}C NMR). Deuterated solvents (Sigma Aldrich) are indicated in the characterization, and chemical shifts are given in ppm. Residual solvent peaks were used as references. All NMR *J* values are given in Hz. COSY and ROESY experiments were recorded to help with the proton assignment.

Mass spectrometric experiments were performed on a Bruker HPLC-TOF (MicroTOF Focus) and on a Synapt G2-S HDMS (Waters Co., Milford, MA, USA) traveling wave ion mobility mass spectrometer. All ions were generated by electrospray ionization (ESI) in the positive and negative modes.

ITC experiments were performed in a MicroCal VP-ITC Micro Calorimeter with the VP Viewer 2000 software. All the titrations were carried out in chloroform: acetonitrile 9:1 solution mixture at 288 K. Titrations were carried out by adding small aliquots (8 μL, 16 s) of a solution of guest into a solution of host in the same solvent. The spacing time was set as 600 s. The concentration of guest solution was approximately sixteen times more concentrated than the host solution. The association constants and the thermodynamic parameters were obtained from the fit of the titration data to either a simple one set of sites binding model or sequential binding sites model by using the Microcal ITC Data Analysis module.
Crystal structure was determined using a Rigaku MicroMax-007HF diffractometer equipped with a PILATUS 2000K detector and a Bruker Apex II Duo equipped with an APEX II detector, both using Mo Ka radiation. Crystal structure solution was achieved using VLD and Patterson methods as implemented in SIR2014 v14.10. Least-squares refinement on F2 using all measured intensities was carried out using the program SHELX-2018/3.

HPLC analysis were performed using an Agilent technologies 1200 series equipped with a BEH HILIC column (3.5 μm, 4.6×150 mm, Waters Xbridge®) with the corresponding precolumn (3.5 μm, 3.9 mm × 5mm, Waters Xbridge®) and a gradient elution (from CH_3CN/H_2O 98:2 to 60:40 in 15 min, 1 mL/min; Injection volume: 5 μL. Detection wavelength 300 nm).

2. Synthesis and characterization data

Tetra-amine tetra-ester aryl-extended calix[4]pyrrole **2** was synthesized using reported procedures.[1] Commercially available terephthaldehyde **3** was distilled under reduced pressure before using it as reactant.

2.1. Synthesis of octa-imine cage 1.

Synthesis in 9:1 $CDCl_3$:CD_3CN solvent mixture.

Freshly distilled terephthalaldehyde **3** solution in 9:1 $CDCl_3$:CD_3CN (250 µL, 8.8 mM) was added to 250 µL of a 4 mM solution of tetra-amino tetra-ester calix[4]pyrrole **2** in the same solvent mixture and placed in an NMR tube. 1,3,5-trimethoxybenzene was added as internal standard (i.s. final concentration = 1 mM).

The ^{1}H NMR of the reaction mixture after 4 days at 300 K showed the signals corresponding to the formation of the cage solvate $(CD_3CN)_2 \subset$**1**. Integration of selected proton signals of the octa-imine cage and those of the i.s. determined a yield of 70% for the assembly of the octa-imine cage **1**.

Synthesis in 9:1 $CDCl_3$:CD_3CN solvent mixture with 0.5 % mol acetic acid.

Freshly distilled terephthalaldehyde **3** solution in 9:1 $CDCl_3$:CD_3CN (250 µL, 8.8 mM) was added to 250 µL of a 4 mM solution of tetra-amino tetra-ester calix[4]pyrrole **2** in the same solvent mixture and placed in an NMR tube. 1,3,5-trimethoxybenzene was added as internal standard (i.s. final concentration = 1 mM). Then, 0.5% mol of acid acetic (1.25 µL, 4 mM) was added to the reaction mixture. The ^{1}H NMR of the reaction mixture after 36 h at r.t. showed the signals corresponding to the formation of the cage **1**. Integration of selected proton signals of the octa-imine cage and those of the i.s. determined a yield of 90% for the assembly of the octa-imine cage **1**.

1: ^{1}H NMR (500 MHz, $CDCl_3$:CD_3CN 9:1) δ(ppm): 8.37 (s, 8H), 7.68 (s, 16H), 7.60 (br. s, 8H), 7.14 (d, *J* = 8.1 Hz, 16H), 7.06 (d, *J* = 8.1 Hz, 16H), 5.96 (s, 16H), 4.06 (q, *J* = 7.1 Hz, 16H), 2.39-2.31 (m, 16H), 2.21 (t, *J* = 7.3 Hz, 16H), 1.55-1.44 (m, 16H), 1.2 (t, *J* = 7.1 Hz, 24H). HR-MS (ESI TOF) m/z: $[M+2H]^{2+}$ Calculated for $C_{168}H_{170}N_{16}O_{16}$ 1333.6485; Found 1333.6630.

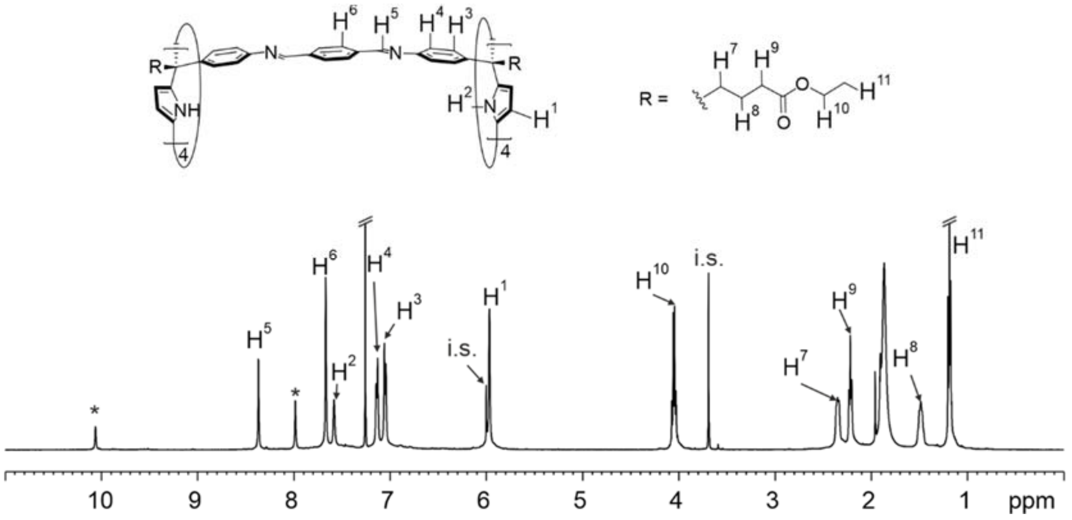

Figure S1. ^{1}H NMR spectrum (500 MHz, at 298 K, $CDCl_3$: CD_3CN 9:1) of cage **1.** * Corresponds to protons of the linker in excess.

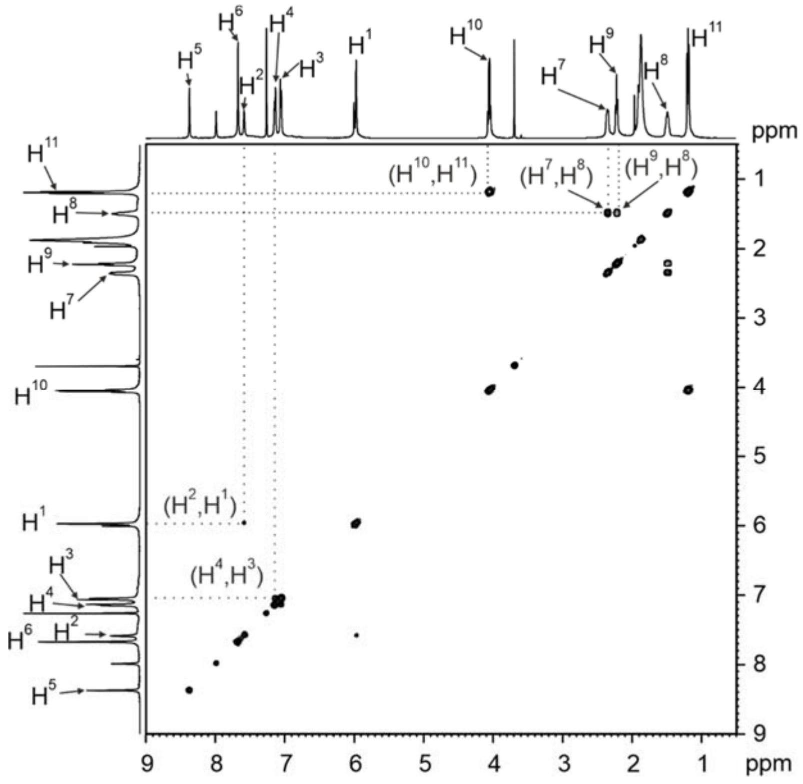

Figure S2. Selected region of ^{1}H-^{1}H COSY NMR (500 MHz, at 298 K, $CDCl_3$: CD_3CN 9:1) of a 2 mM solution of octa-imine cage **1** with an excess of terephthaldehyde linker **3**. See **Figure S1.** for protons assignment.

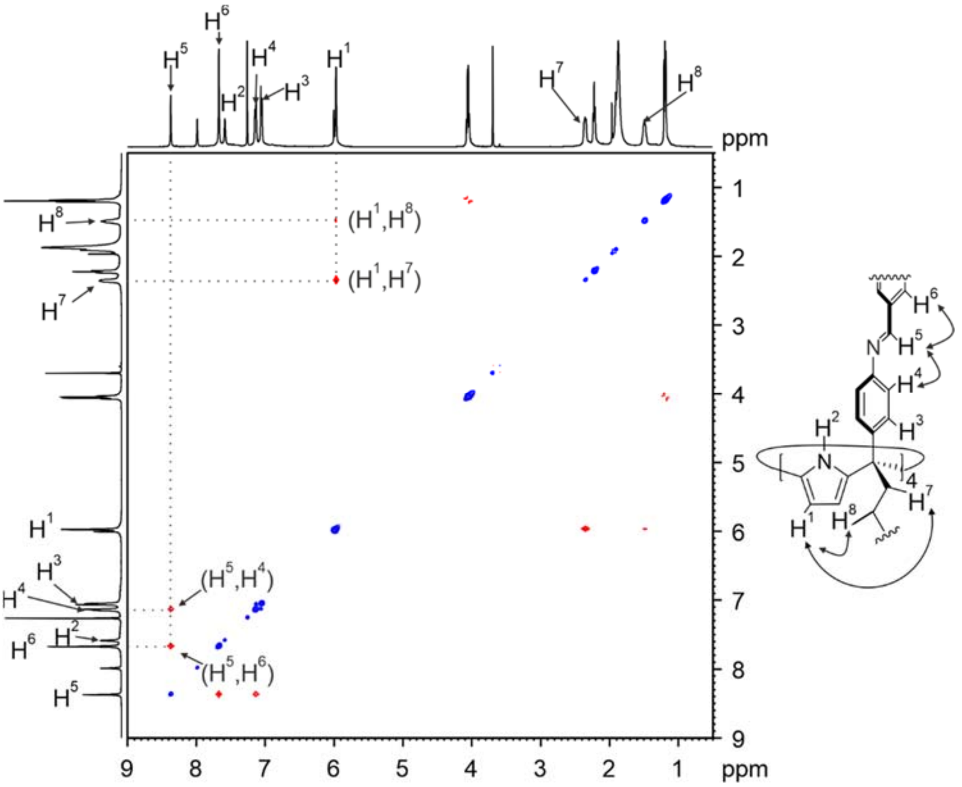

Figure S3. Selected region of ^{1}H-^{1}H ROESY NMR (500 MHz, at 298 K, $CDCl_3$:CD_3CN 9:1, D8 = 0.30 s) of a 2 mM solution of octa-imine cage **1**. See **Figure S1** for protons assignment.

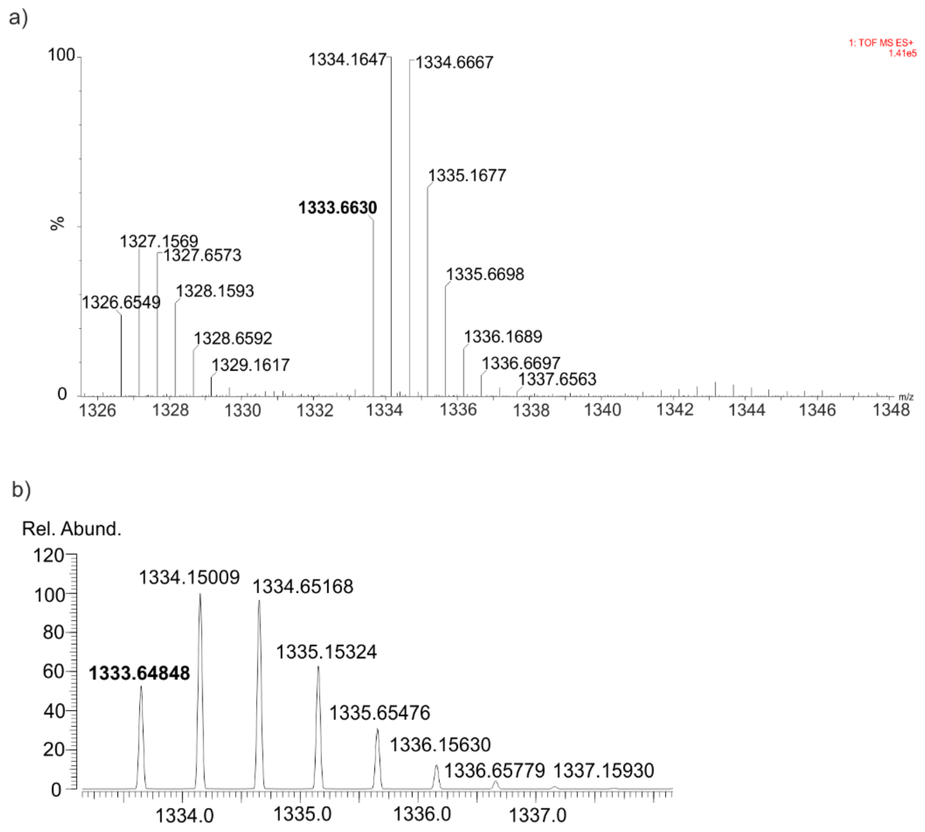

Figure S 4. Experimental (a) and theoretical (b) isotopic distribution of $[M+2H]^{2+}$ ion peak (M = **1** = $C_{168}H_{168}N_{16}O_{16}$). The exact mass for the monoisotopic peak is highlighted in bold.

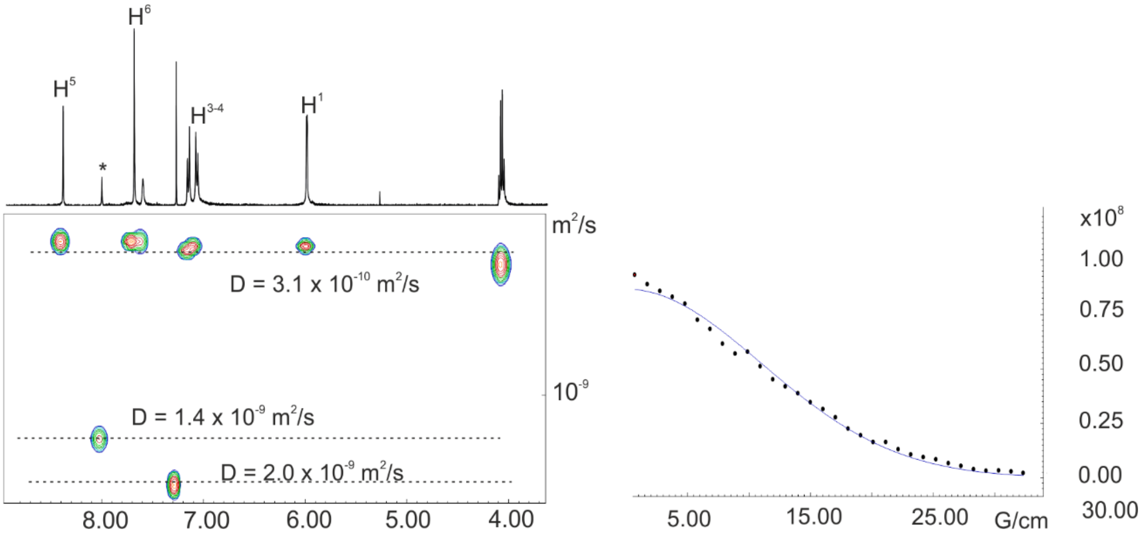

Figure S5. Left) ^{1}H pseudo-2D DOSY NMR profile of octa-imine cage **1** in a 9:1 $CDCl_3$:CD_3CN mixture at 289 K and millimolar concentration. Right) Fit of the data to a mono-exponential function. *Residual peak related to the excess of terephthaldehyde linker **3**.

Aiming to experimentally prove the inclusion of CH_3CN molecules in the self-assembled octa-imine cage **1**, we performed a 1D-GOESY experiment in a 9:1 $CDCl_3$:CH_3CN solution. Notably, the replacement of CD_3CN for CH_3CN should induce the formation of the putative $(CH_3CN)_n\subset$**1** cage complex. The selective excitation of the singlet corresponding to the CH_3CN protons produced a 1D-GOESY spectrum displaying, in addition to the excited singlet, a low-intensity in-phase broad singlet resonating at $\delta = 0.4$ ppm ($\Delta\delta$ = -1.6 ppm) (Figure S6). We assigned this signal to the protons of CH_3CN molecules in the polar hemispheres of the octa-imine cage **1** experiencing the shielding effect of the four *meso*-aromatic substituents. The included acetonitrile molecule(s) were involved in a chemical exchange with those in the bulk solvent, producing saturation transfer. Hence, the chemical exchange process showed fast dynamics on the 1D-GOESY time scale but was slow on the ^{1}H chemical shift counterpart. This result helped us to identify and assign a broad-singlet in the ^{1}H NMR spectrum of **1** acquired in the 9:1 $CDCl_3$:CH_3CN solution mixture. The singlet broadening was detrimental to obtaining an accurate integration value. We hypothesized that, most likely, one molecule of CH_3CN was included in each of the two polar hemispheres of **1**. However, we cannot rule out the inclusion of a third molecule of CH_3CN in the central aromatic cavity. If included, this third molecule might experience a chemical exchange with those in the bulk solvent that is fast on the ^{1}H NMR timescale and does not produce a second saturation-transfer peak in the 1D GOESY spectrum.

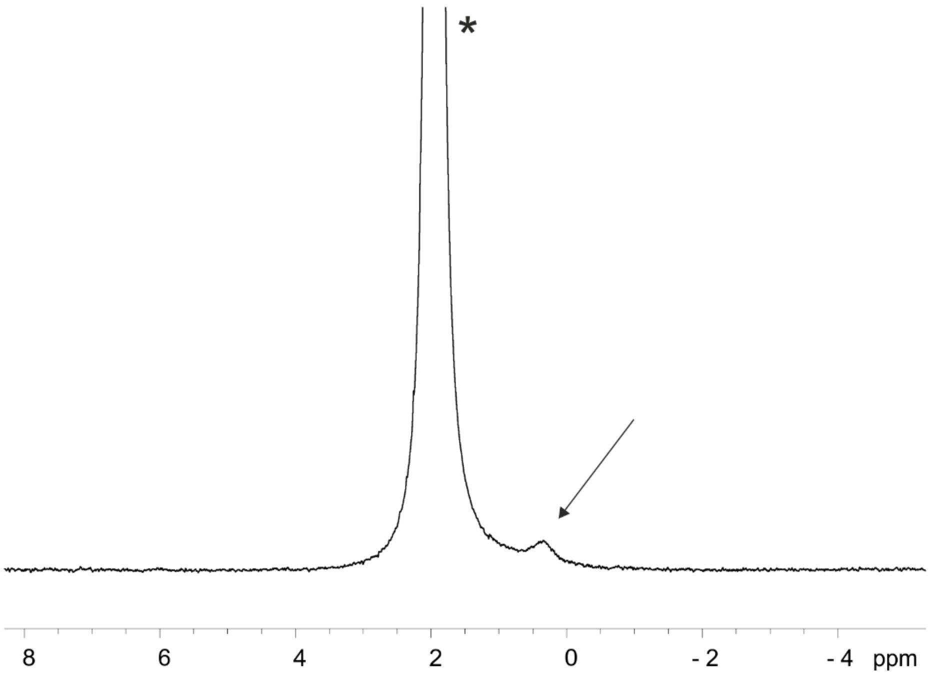

Figure S 6. 1D GOESY NMR (500 MHz, 9:1 $CDCl_3$:CH_3CN, 289 K) spectrum of octa-imine capsule **1.** t_{mix} = 0.3 s. * Residual solvent peak.

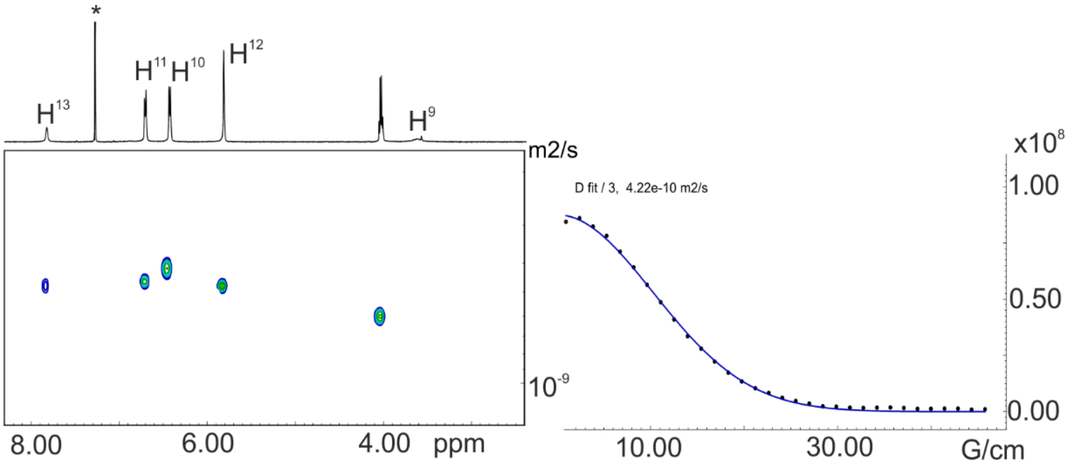

Figure S7. Left) ^{1}H pseudo-2D DOSY NMR profile of tetra-amine tetra-ester aryl-extended calix[4]pyrrole **2** in a 9:1 $CDCl_3$:CD_3CN mixture at 289 K and millimolar concentration. Right) Fit of the data to a mono-exponential function ($D = 4.6 \pm 0.2 \times 10^{-10}$ m^2s^{-1}).

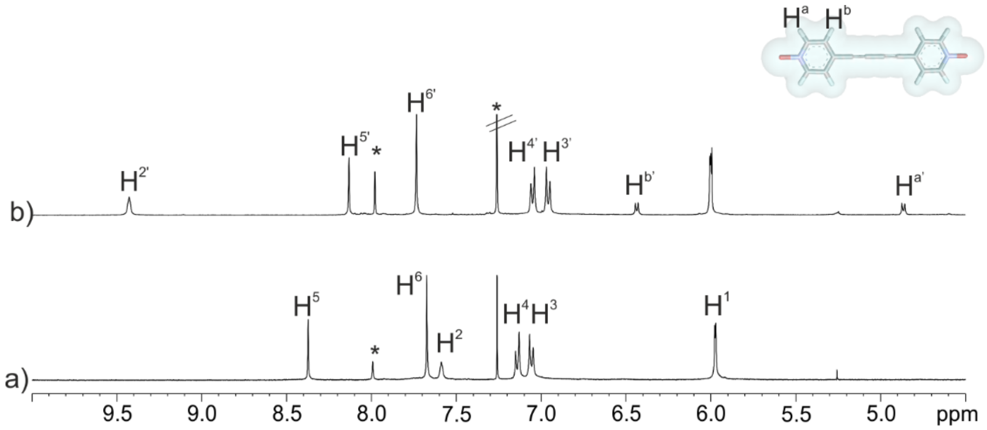

Figure S8. ^{1}H NMR spectrum (500 MHz, at 298 K, $CDCl_3$:CD_3CN 9:1) of the octa-imine cage **1** with a) 0 equiv. and b) 1 equiv. of 4,4-(buta-1,3-diyne-1,4-diyl)bis-pyridine *N*-oxide guest **8**. Primed protons correspond to complex **8⊂1**.

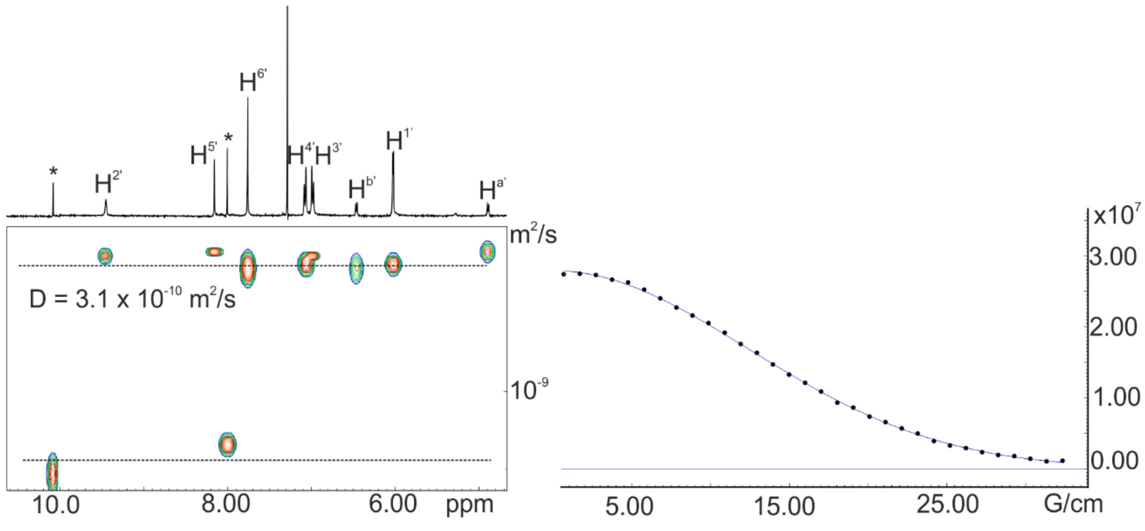

Figure S9. Left) ^{1}H pseudo-2D DOSY NMR profile of octa-imine cage **1** with 4,4-(buta-1,3-diyne-1,4-diyl)bis-pyridine *N*-oxide guest included in $CDCl_3$:CD_3CN mixture at 289K and millimolar concentration. Right) bottom - Fit of the data to a mono-exponential function (D = 3.1 ± 0.06 × 10^{-10} m^2s^{-1}).

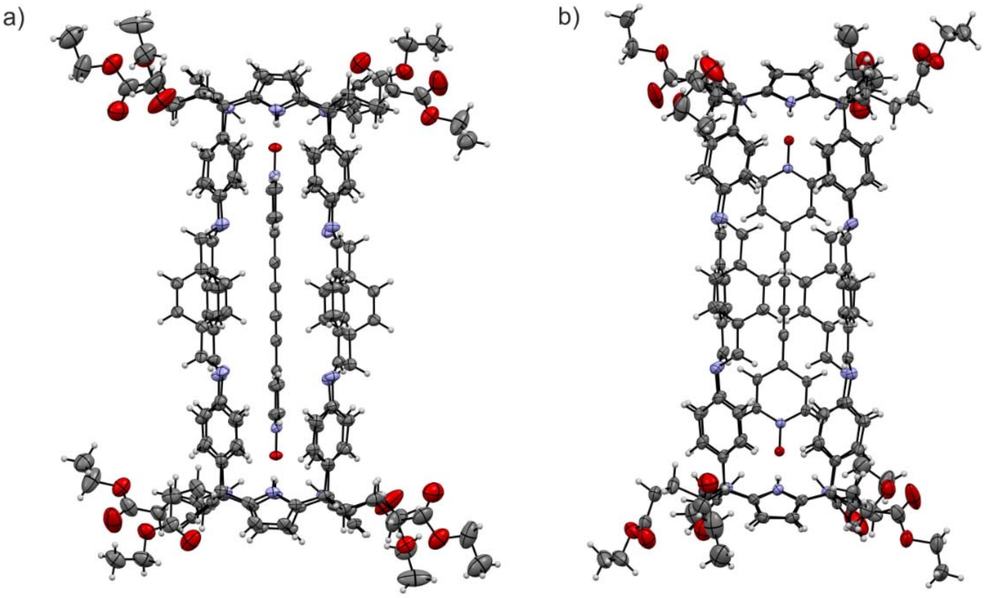

Figure S 10. Side-views of the X-ray crystal structure of the octa-imine **8⊂1** complex. Thermal ellipsoids for C, N, and O atoms are set at 50% probability. H atoms are shown as spheres of 0.30 Å in diameter.

2.2. Synthesis of pyridyl *N*-oxide derivatives

General procedure for the synthesis of para-substituted pyridyl N-oxides: para-substituted pyridyl *N*-oxide derivatives were obtained by oxidation of the corresponding pyridine analogue using the following general procedure. The para-substituted pyridine derivative (1 mmol) was dissolved in a 1:1 mixture of water/2-butone (60 mL). Then $NaHCO_3$ (1.68 g, 20 mmol, 20 equiv.) was added into the two-phase solution under stirring. A water solution of OXONE® (2.50 g, 4 mmol, 4 equiv.) in 20 mL was added to the mixture in 30 minutes under stirring. After 24 h, the mixture was diluted with chloroform (50 mL) and the water phase was washed with chloroform (2 x 50mL). The organic layers were collected, dried over anhydrous Na_2SO_4, filtered, and concentrated under vacuum. The crude was purified by column chromatography (neutral alumina, chloroform: i-propanol 95:5) to obtain the pyridyl *N*-oxide derivates in 40-60% yield.

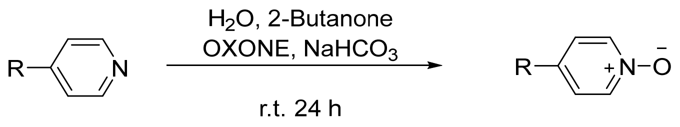

Scheme S1. Synthesis of pyridyl *N*-oxide derivatives **4a** (R = N_3), **4b** (R = -CH_2N_3), **4c** (R = -$(CH_2)_2N_3$), **5** (R = -C≡CH), **10** (R = -(C_6H_4)-C≡CH).

*General procedure for the synthesis of Bis-pyridyl bis-N-oxide 1,4-1,2,3-triazole derivatives **6a-6c**:* Bis-pyridyl bis-*N*-oxide 1,4-1,2,3-triazole derivatives were obtained by 1,3-dipolar cycloaddition reaction between the para-substituted pyridine *N*-oxide precursors with alkyne and azide terminal groups. In general, the pyridyl *N*-oxide alkyne derivative (0.1 mmol) and pyridyl *N*-oxide azide derivative (0.1 mmol) were dissolved in 5 mL of dry dichloromethane under argon. Then, $Cu(CH_3CN)_4PF_6$ (5 μmol, 0.05 equiv.) and tris[(1-benzyl-1H-1,2,3-triazol-4-yl)methyl]amine (TBTA) (5 μmol, 0.05 equiv.) were added to the mixture and stirred for 2 h at r.t. protected from light. A white precipitate was formed in the reaction mixture. The white precipitate was collected via filtration and washed with dichloromethane (5 mL) and acetonitrile (5 mL) without further purification.

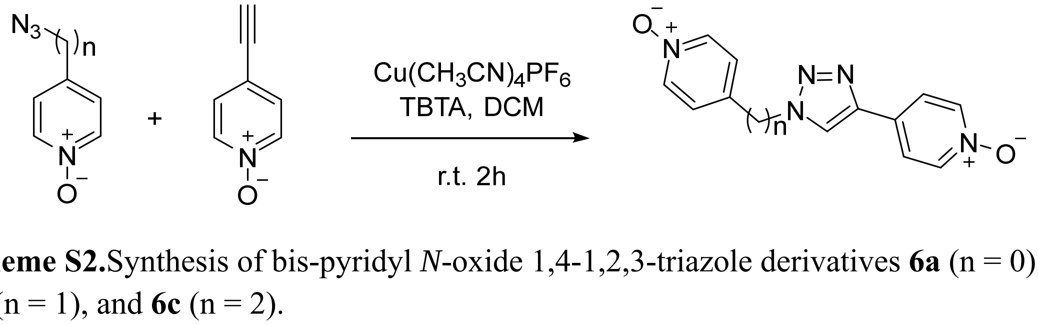

Scheme S2. Synthesis of bis-pyridyl *N*-oxide 1,4-1,2,3-triazole derivatives **6a** (n = 0), **6b** (n = 1), and **6c** (n = 2).

4a: 4-azidopyridine was obtained using previously described procedures[2] and used as starting material to produce compound **4a** following the general procedure described above.

^{1}H NMR (400 MHz, 298 K, $CDCl_3$): δ (ppm) = 8.07 (d, J = 7.5 Hz, 2H), 6.89 (d, J = 7.5 Hz, 2H). ^{13}C{^{1}H} NMR (100 MHz, 298 K, $CDCl_3$): δ (ppm) =140.3, 138.8, 116.6. HR-MS (ESI TOF) m/z: $[M+Na]^+$ calculated for $C_5H_4N_4NaO^+$ 159.0277, found 159.0282.

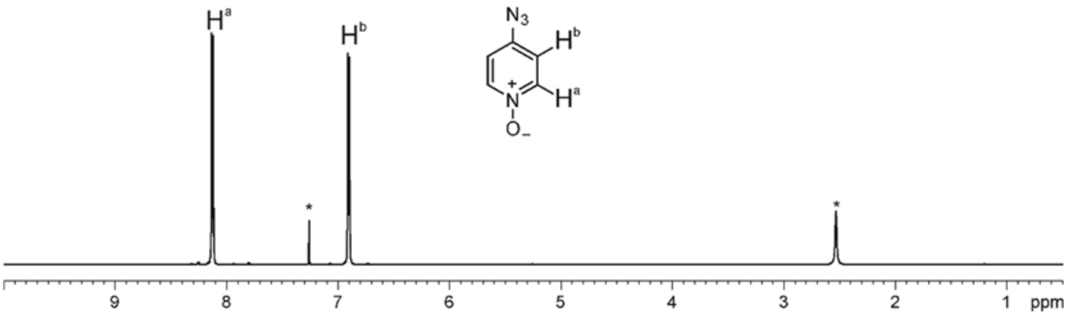

Figure S11. ^{1}H NMR spectrum (400 MHz, 298 K, $CDCl_3$) of **4a**. *Residual solvent peaks.

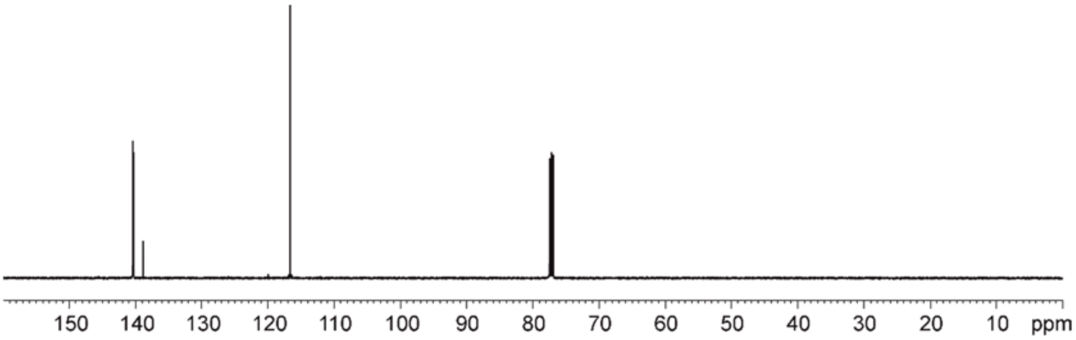

Figure S12. ^{13}C{^{1}H} NMR spectrum (100 MHz, 298 K, $CDCl_3$) of **4a**.

4b: 4-Azidomethyl-pyridine **9a** was prepared using a previously reported procedure[3] and used as starting material to prepare compound **4b** using the general procedure described above.

^{1}H NMR (400 MHz, 298 K, $CDCl_3$): δ (ppm) = 8.19 (d, J = 6.8 Hz, 2H), 7.22 (d, J = 6.8 Hz, 2H), 4.39 (s, 2H). ^{13}C{^{1}H} NMR (100 MHz, 298 K, $CDCl_3$): δ (ppm) =139.4, 134.3, 125.1, 52.43. HR-MS (ESI TOF) m/z: $[M+H]^+$ calculated for $C_6H_7N_4O^+$=151.0620, found 151.0613.

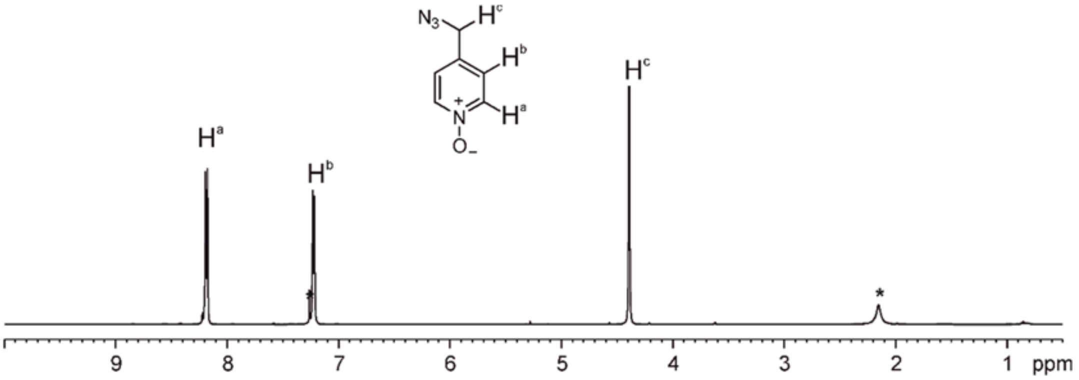

Figure S13. ^{1}H NMR spectrum (400 MHz, 298 K, $CDCl_3$) of **4b**. *Residual solvent peaks.

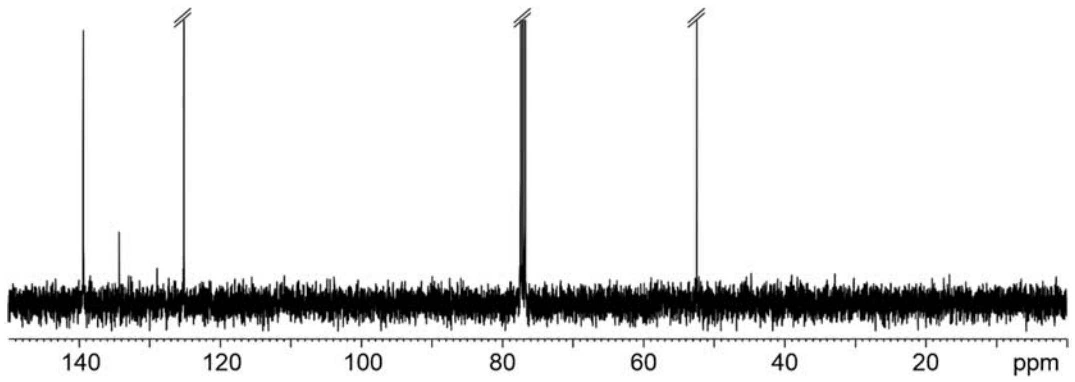

Figure S14. $^{13}C\{^{1}H\}$ NMR spectrum (100 MHz, 298 K, $CDCl_3$) of **4b**.

5: commercially available 4-ethynylpyridine was used for the preparation of compound **5** using the general procedure described above.

^{1}H NMR (400 MHz, 298 K, $CDCl_3$): δ (ppm) = 8.12 (d, *J* = 7.2 Hz, 2H), 7.32 (d, *J* = 7.2 Hz, 2H), 3.39 (s, 1H). $^{13}C\{^{1}H\}$ NMR (100 MHz, 298K, $CDCl_3$): δ (ppm) = 139.2, 129.1, 119.8, 83.5, 80.0. HR-MS (ESI TOF) m/z: $[M+H]^+$ calculated for $C_7H_6NO^+$ = 120.0449, found 120.0447.

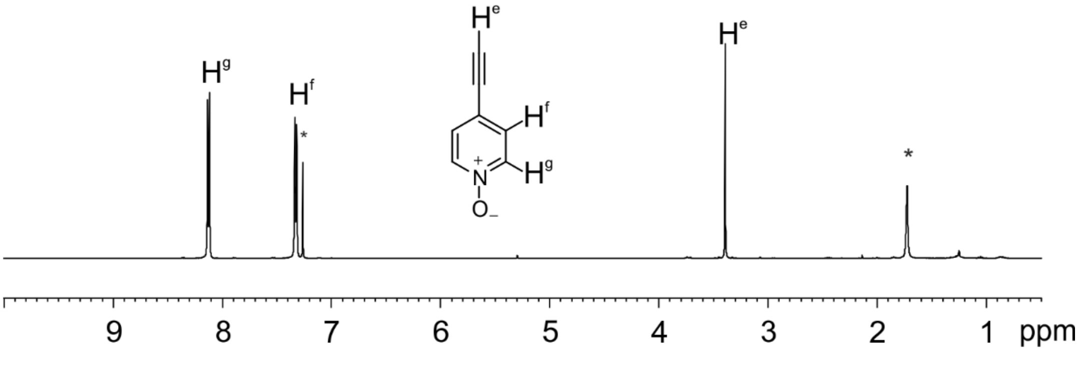

Figure S15. ^{1}H NMR spectrum (400 MHz, 298 K, $CDCl_3$) of **5**. *Residual solvent peaks.

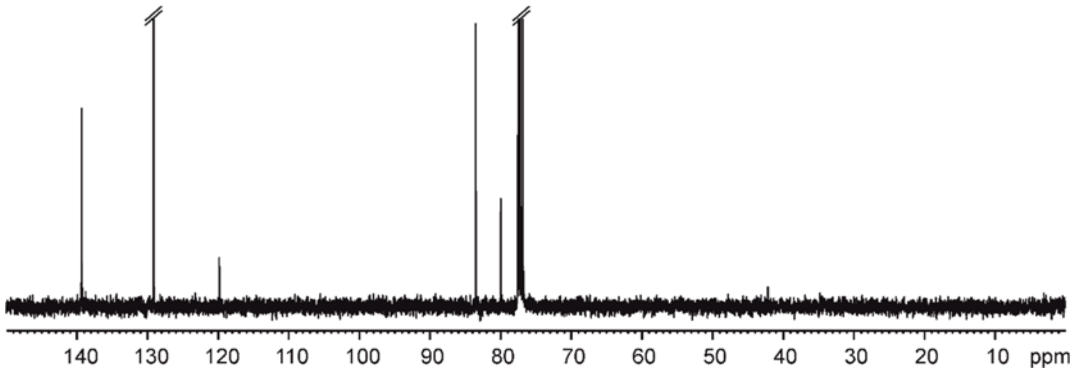

Figure S16. $^{13}C\{^{1}H\}$ NMR spectrum (100 MHz, 298 K, $CDCl_3$) of **5**.

10: commercially available 4-(4-ethynylphenyl)-pyridine was used for the preparation of compound **10** using the general procedure described above.

^{1}H NMR (400 MHz, 298 K, $CDCl_3$): δ (ppm) = 8.26 (d, *J* = 7.2 Hz, 2H), 7.60 (d, *J* = 8.4 Hz, 2H), 7.54 (d, *J* = 8.4 Hz, 2H), 7.50 (d, *J* = 7.2 Hz, 2H), 3.20 (s, 2H). $^{13}C\{^{1}H\}$ NMR (100 MHz, 298K, $CDCl_3$): δ (ppm) =139.6, 137.9, 136.4, 133.2, 126.4, 123.8, 123.3, 82.9, 79.3. HR-MS (ESI TOF) m/z: $[M+H]^+$ calculated for $C_{13}H_{10}NO^+$= 196.0762, found 196.0761.

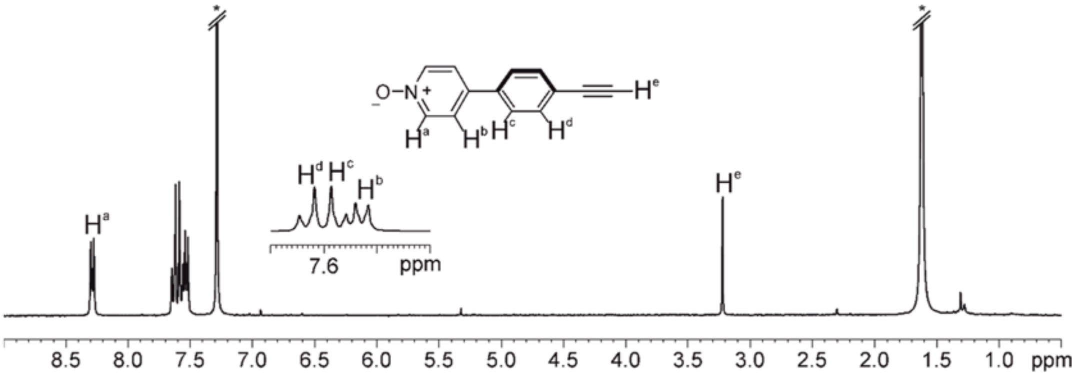

Figure S17. ^{1}H NMR spectrum (400 MHz, 298 K, $CDCl_3$) of **10**. *Residual solvent peaks.

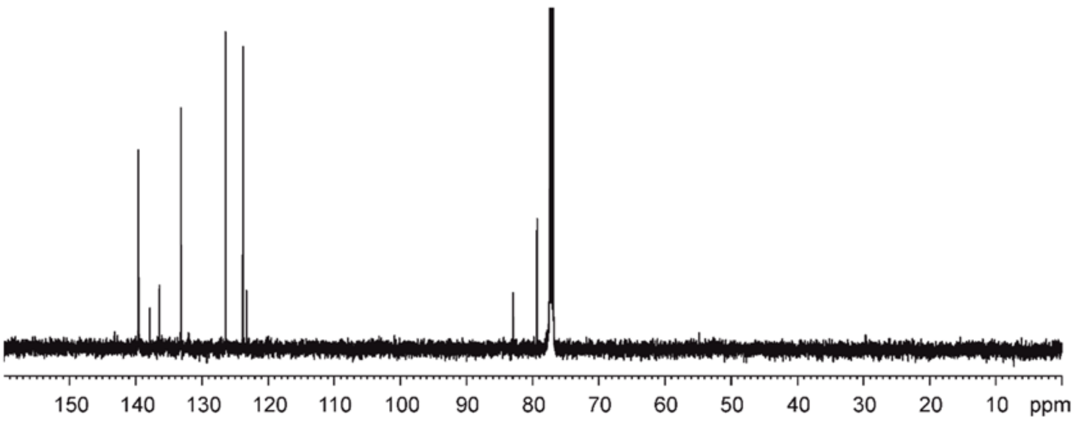

Figure S18. $^{13}C\{^1H\}$ NMR spectrum (100 MHz, 298 K, $CDCl_3$) of **10**.

6a: *N*-oxides **4a** and **5** were used for the preparation of compound **6a** using the general procedure described above for the synthesis of bis-pyridyl bis-*N*-oxide 1,4-1,2,3-triazole derivatives.

^1H NMR (400 MHz, 298 K, d_6-DMSO): δ (ppm) = 9.55 (s, 1H), 8.46 (d, J = 7.3 Hz, 2H), 8.35 (d, J = 7.0 Hz, 2H), 7.99 (d, J = 7.3 Hz, 2H), 7.88 (d, J = 7.0 Hz, 2H) ppm. $^{13}C\{^1H\}$ NMR (100 MHz, 298K, d_6-DMSO): δ (ppm) =144.5, 140.2, 139.5, 131.9, 126.2, 122.5, 120.9, 117.2. HR-MS (ESI TOF) m/z: $[M+Na]^+$ calculated for $C_{12}H_9N_5NaO_2^+$ = 278.0660, found 278.0648.

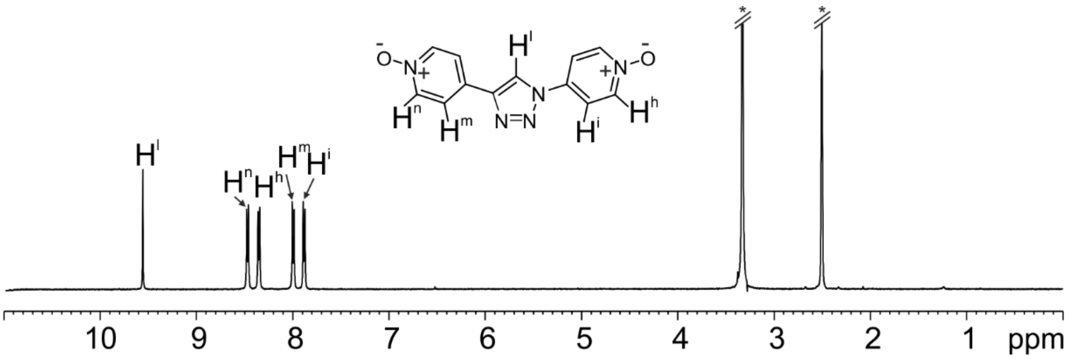

Figure S19. ^1H NMR spectrum (400 MHz, 298 K, d_6-DMSO) of **6a**. *Residual solvent peaks.

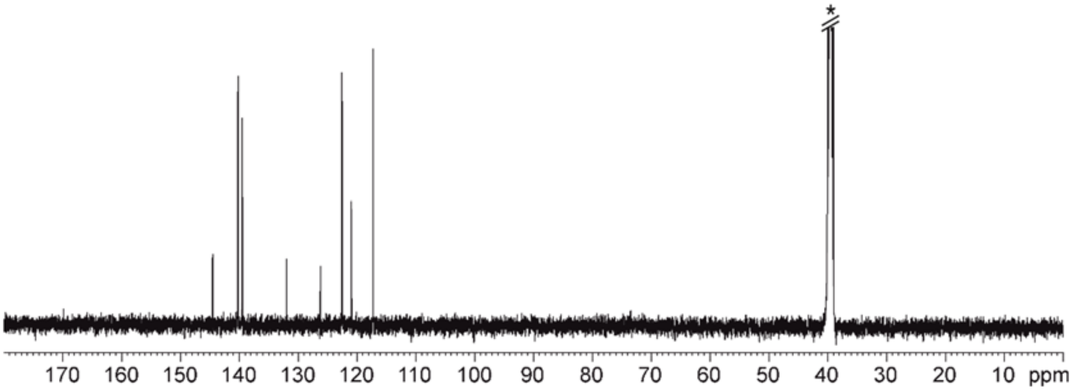

Figure S 20. $^{13}C\{^1H\}$ NMR spectrum (100 MHz, 298 K, d_6-DMSO) of **6a**. *Residual solvent peaks

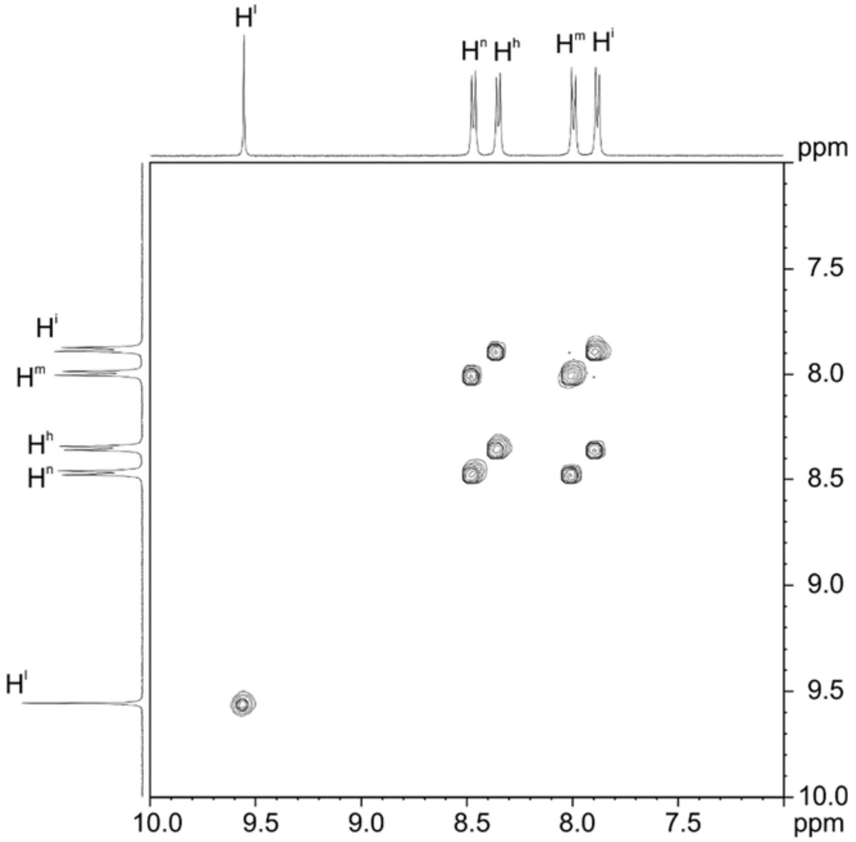

Figure S21. Selected region of ^{1}H-^{1}H COSY NMR (400 MHz, 298 K, *d*$_6$-DMSO) of **6a**.

6b: *N*-oxides **5** and **4b** were used for the preparation of compound **6b** using the general procedure described above for the synthesis of bis-pyridyl bis-*N*-oxide 1,4-1,2,3-triazole derivatives,

^{1}H NMR (400 MHz, 298 K, *d*$_6$-DMSO): δ (ppm) = 8.80 (s, 1H), 8.28 (d, *J* = 6.6 Hz, 2H), 8.23 (d, *J* = 6.4 Hz, 2H), 7.85 (d, *J* = 6.6 Hz, 2H), 7.35 (d, *J* = 6.4 Hz, 2H), 5.71 (s, 2H) ppm. $^{13}C\{^{1}H\}$ NMR (100 MHz, 298K, *d*$_6$-DMSO): δ (ppm) = 144.1, 139.7, 139.4, 133.2, 127.5, 126.5, 123.7, 122.8, 51.52. HR-MS (ESI TOF) m/z $[M+Na]^+$ calculated for $C_{13}H_{11}N_5NaO_2$: 292.0805, found 292.0806.

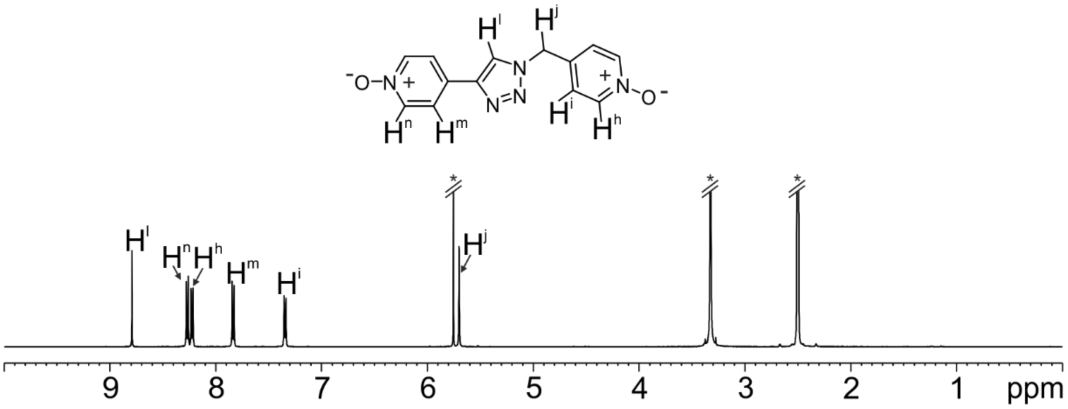

Figure S22. ^{1}H NMR spectrum (400 MHz, 298 K, d_6-DMSO) of **6b**. *Residual solvent peaks.

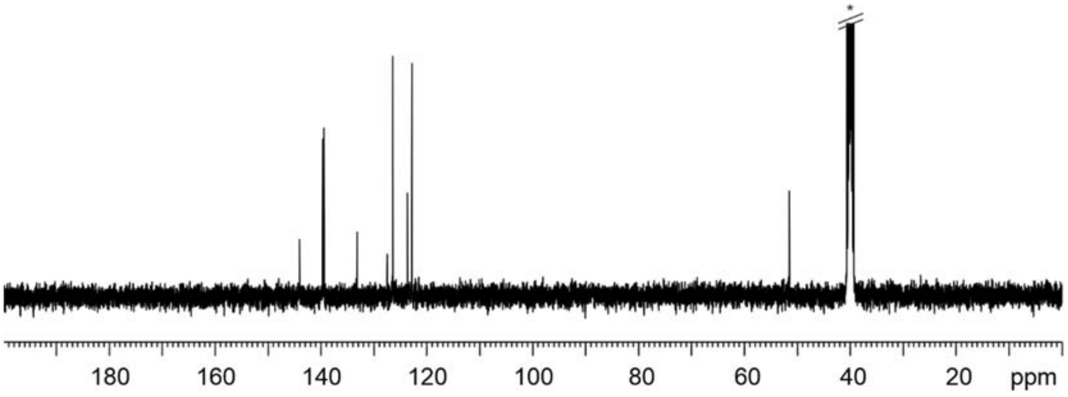

Figure S 23. ^{13}C{^{1}H} NMR spectrum (100 MHz, 298 K, d_6-DMSO) of **6b**.

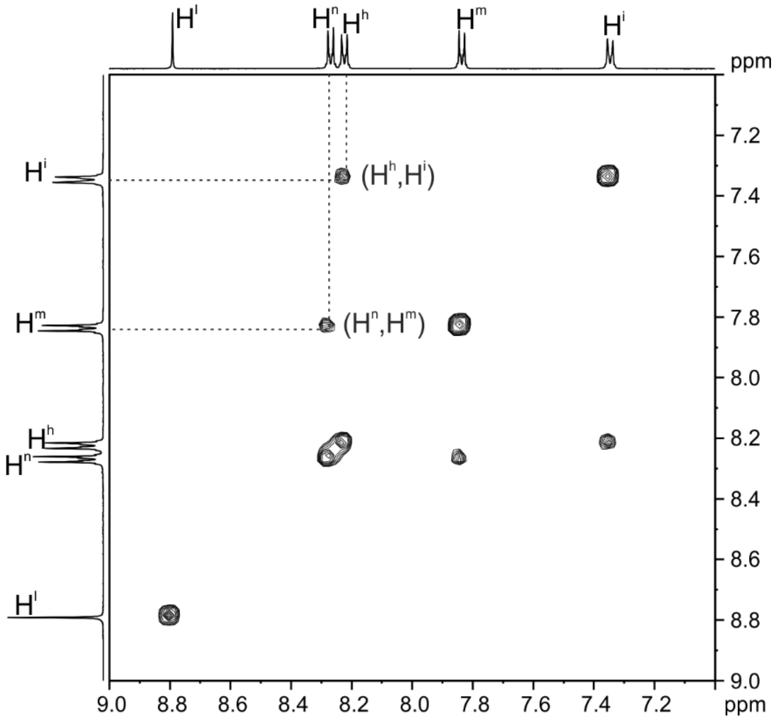

Figure S24. Selected region of ^1H-^1H COSY NMR (400 MHz, 298 K, d_6-DMSO) of **6b**.

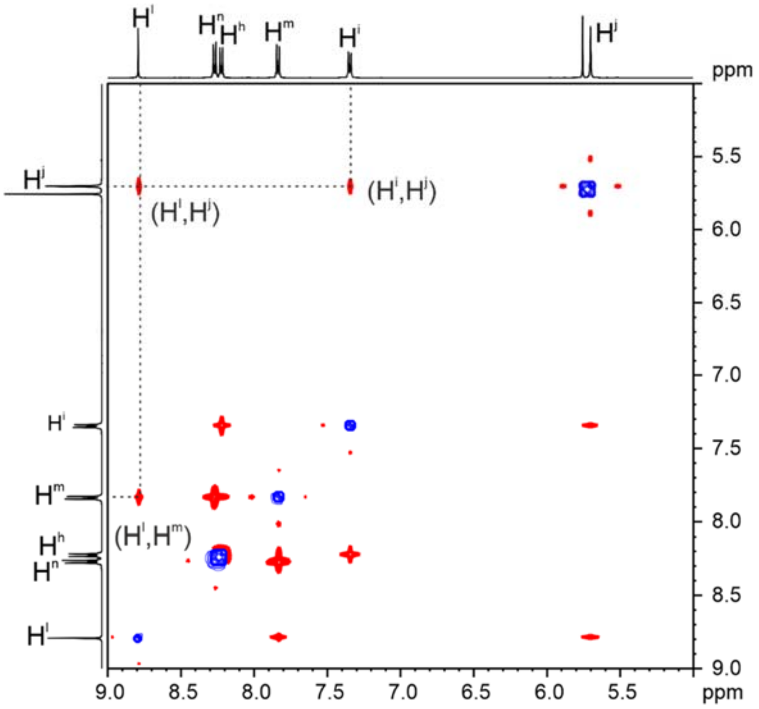

Figure S25. Selected region of ^1H-^1H ROESY NMR (400 MHz, 298 K, d_6-DMSO, D8 = 0.30 s) of **6b**.

6c: *N*-oxides **5** and **4c** were used for the preparation of compound **6c** using the general procedure described above for the synthesis of bis-pyridyl bis-*N*-oxide 1,4-disubstituted 1,2,3-triazole derivatives.

^1H NMR (400 MHz, 298 K, d_6-DMSO): δ (ppm) = 8.67 (s, 1H), 8.25 (d, J = 7.0 Hz, 2H), 8.10 (d, J = 6.8 Hz, 2H), 7.78 (d, J = 7.0 Hz, 2H), 7.23 (d, J = 6.8 Hz, 2H), 4.71 (t, J = 7.0 Hz, 2H) 3.21 (t, J = 7.0 Hz, 2H) ppm. ^{13}C NMR (100 MHz, 298K, d_6-DMSO): δ (ppm) = 143.6, 139.7, 138.9, 136.0, 127.7, 127.2, 123.2, 122.7, 50.20, 34.11. HR-MS (ESI TOF) m/z: $[M+H]^+$ calculated for $C_{14}H_{14}N_5O_2^+$ = 284.1143, found 284.1142.

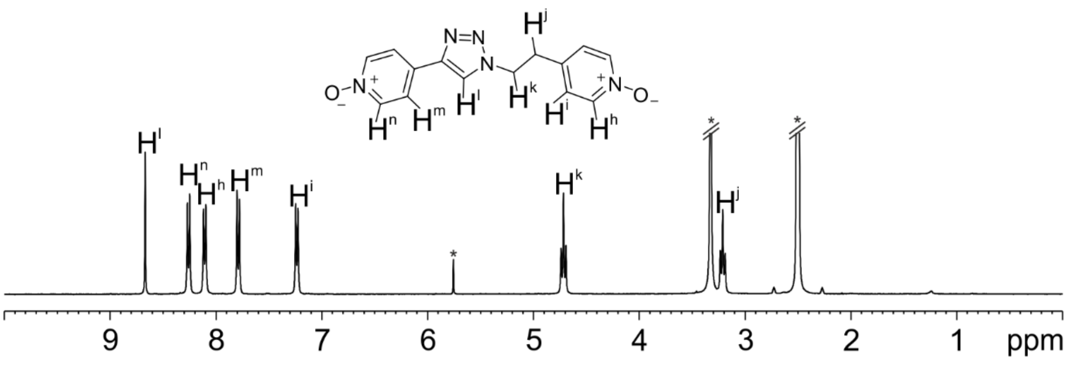

Figure S 26. ^1H NMR spectrum (400 MHz, 298 K, d_6-DMSO) of **6c**. *Residual solvent peaks.

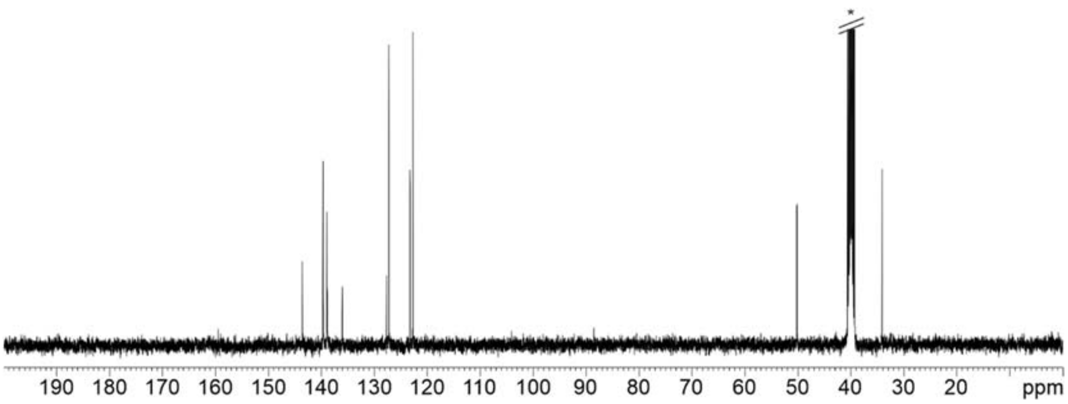

Figure S 27. $^{13}C\{^{1}H\}$ NMR spectrum (100 MHz, 298 K, d_6-DMSO) of **6c**.

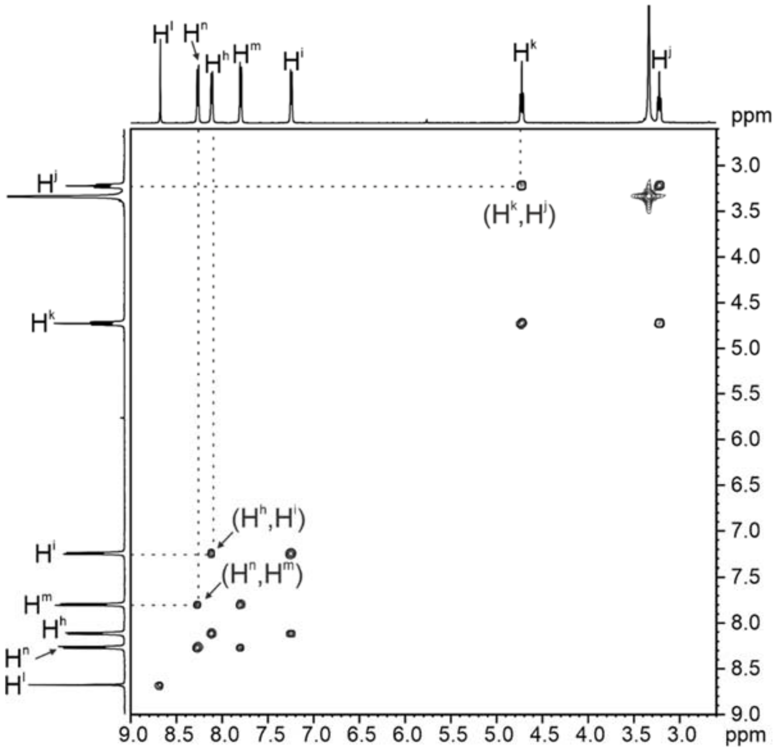

Figure S 28. Selected region of ^{1}H-^{1}H COSY NMR (400 MHz, 298 K, d_6-DMSO) of **6c**.

3. **Binding studies and characterization of octa-imine cage 1 with monotopic and ditopic pyridine *N*-oxide derivatives in CDCl3: CD3CN 9:1 mixture.**

3.1. Binding studies of 4-azido pyridine *N*-oxide 4a with 1.

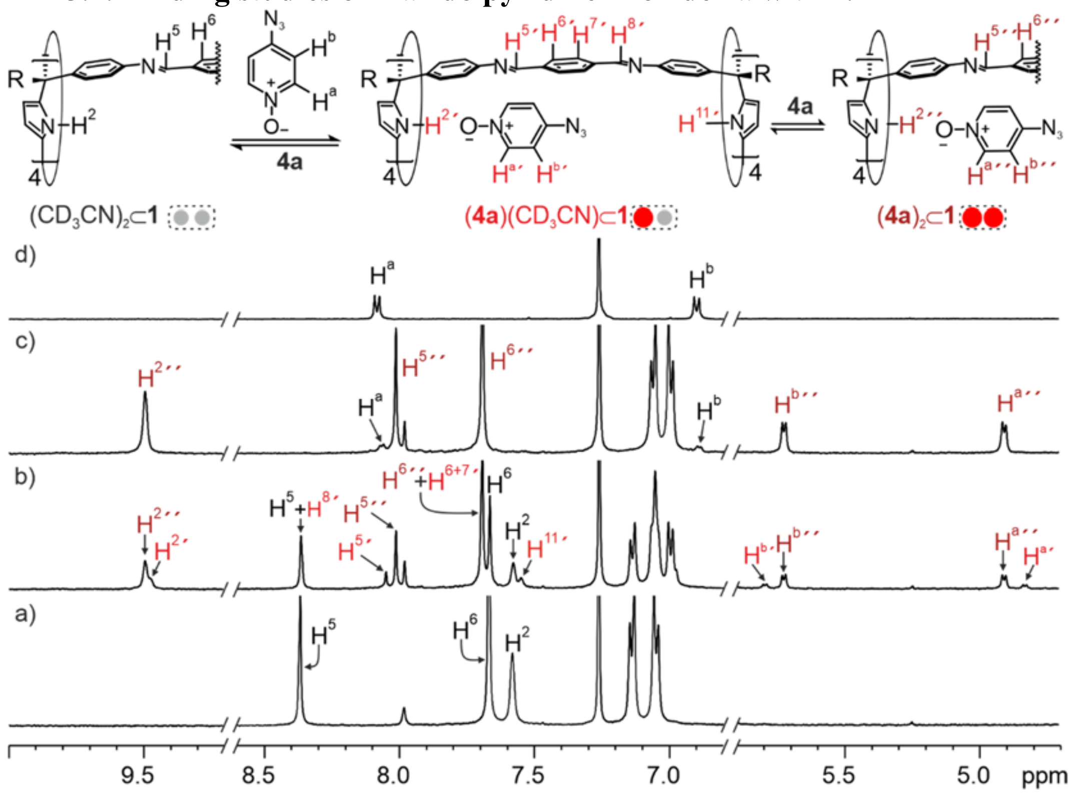

Figure S29. Selected regions of ^{1}H NMR (400 MHz, 298 K, CDCl3:CD3CN 9:1) spectra of the titration of a 2 mM solution of octa-imine cage **1**, upon addition of a) 0 equiv., b) 1 equiv. ; c) 2.1 equiv. of **4a**. Spectrum d) corresponds to the free **4a** in the same solvent mixture. Primed and double-primed protons correspond to the 1:1 and 2:1 complexes, correspondingly.

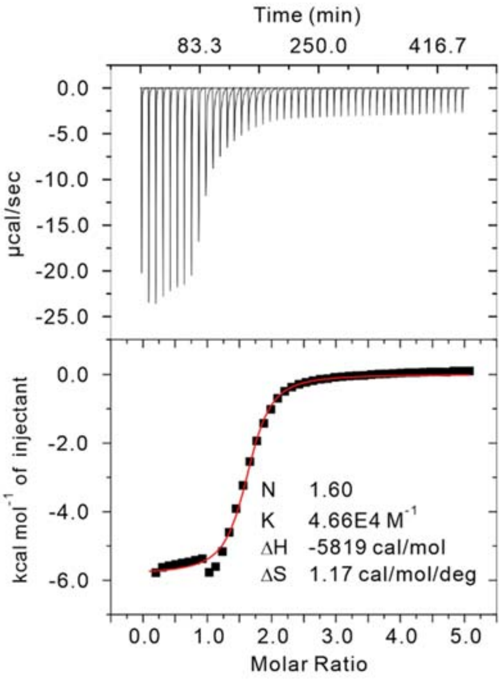

Figure S 30. Top- Traces of the raw data (heat vs time) of the ITC experiment of cage **1** ([cell] =1 × 10^{-3} M) with **4a** ([syringe] =1.6 × 10^{-2} M). The solutions were prepared using a chloroform: acetonitrile 9:1 solvent mixture. Bottom- Normalized integrated heat (black squares) vs. **4a**/**1** molar ratio. The experimental data were fit to one set of sites binding model (red line).

We conducted isothermal titration calorimetry (ITC) experiments to characterize the binding process of 1 with 4a thermodynamically. We obtained a single sigmoidal binding isotherm with an inflection point close to a **4a/1** molar ratio of 2 (Figure S30). The fit of the calorimetric data to the one set of sites binding model implemented in the Microcal software[4] returned the average microscopic binding constant value for the two sites as $K_{average} = 4.6 \times 10^4$ M^{-1}. The magnitude of the value is in good agreement with the average of those estimated from the speciation profiles of the ^{1}H NMR titration.[5]

3.2. Binding studies of 4-azidomethyl pyridine *N*-oxide 4b with 1.

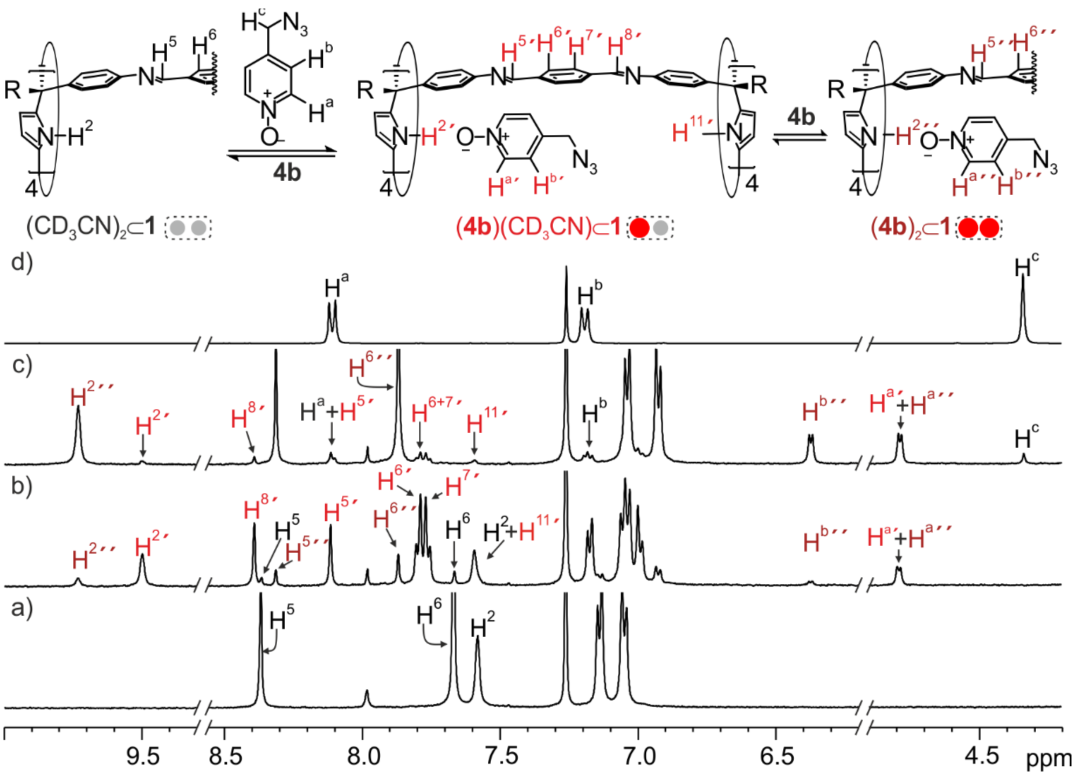

Figure S31. Selected regions of ^{1}H NMR (400 MHz, 298 K, $CDCl_3$:CD_3CN 9:1) spectra of the titration of a 2 mM solution of octa-imine cage **1**, upon addition of a) 0 equiv., b) 1 equiv. ; and c) 2.2 equiv. of **4b**. Spectrum d) corresponds to the free **4b** in the same solvent mixture. Primed and double-primed protons correspond to the 1:1 and 2:1 complexes, correspondingly.

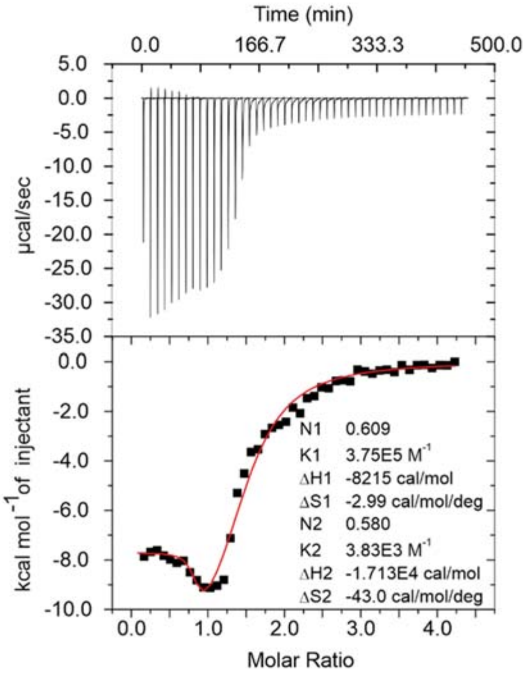

Figure S 32. Top- Traces of the raw data (heat *vs* time) of the ITC experiment of cage **1** ([cell] = 1×10^{-3} M) with **4b** ([syringe]= 1.6×10^{-2} M). The solutions were prepared using a chloroform: acetonitrile 9:1 solvent mixture. Bottom- Normalized integrated heat (black squares) vs. **4b**/**1** molar ratio. Experimental data were fit to a sequential two-binding sites model (red line).

3.3. Binding studies of 4-azidoethyl pyridine *N*-oxide 4c with 1.

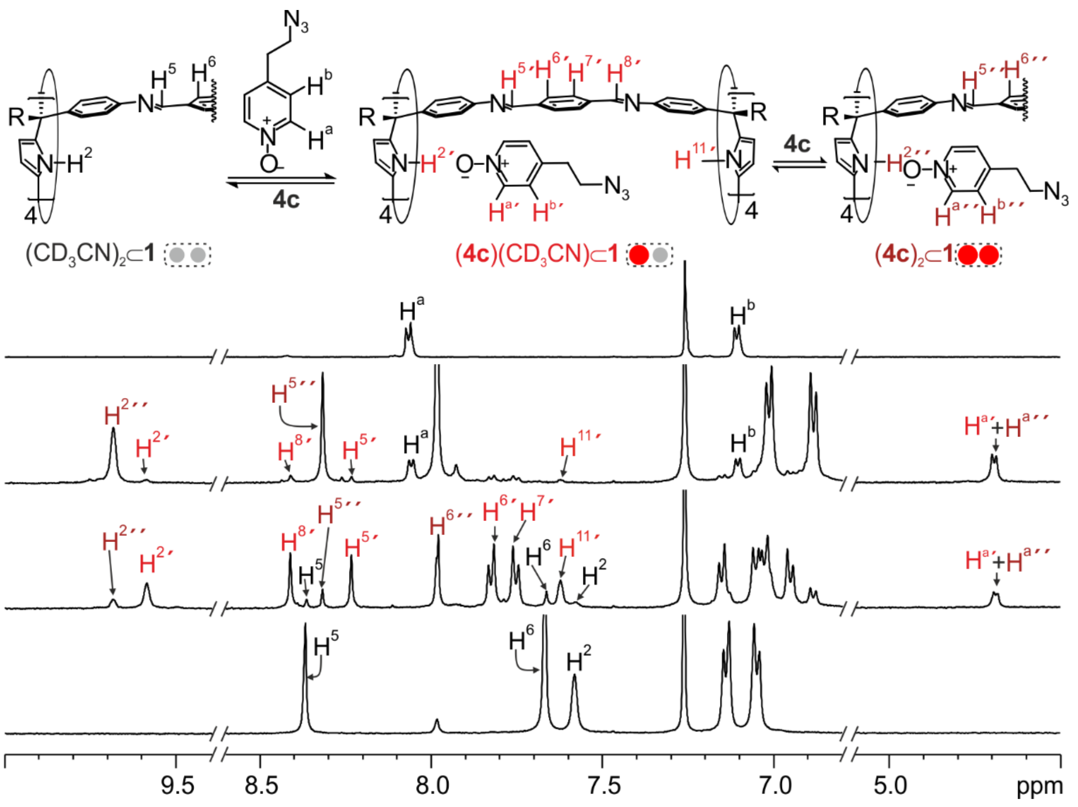

Figure S 33. Selected regions of ^{1}H NMR (400 MHz, 298 K, $CDCl_3$:CD_3CN 9:1) spectra of the titration of a 2 mM solution of octa-imine cage **1**, upon addition of a) 0 equiv., b) 1 equiv., and c) 2.0 equiv. of **4c**. Spectrum d) corresponds to the free **4c** in the same solvent mixture. Primed and double-primed protons correspond to the 1:1 and 2:1 complexes, correspondingly.

3.4. Binding studies of 4-ethynyl pyridine *N*-oxide 5 with 1.

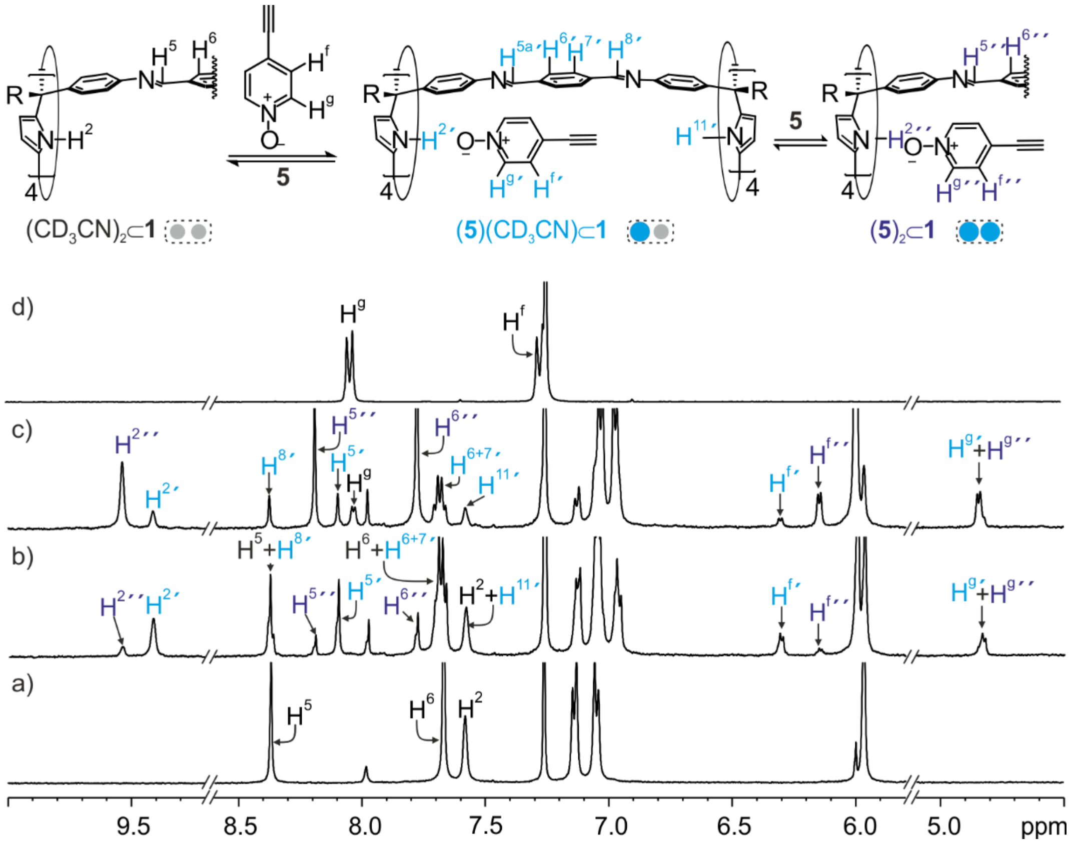

Figure S34. Selected regions of ^{1}H NMR (400 MHz, 298 K, $CDCl_3$:CD_3CN 9:1) spectra of the titration of a 2 mM solution of octa-imine cage **1**, upon addition of a) 0 equiv., b) 1 equiv., c) 3 equiv. of **5.** Spectrum d) corresponds to the free **5** in the same solvent mixture. Primed and double-primed proton signals correspond to those of the 1:1 and 2:1 complexes, respectively.

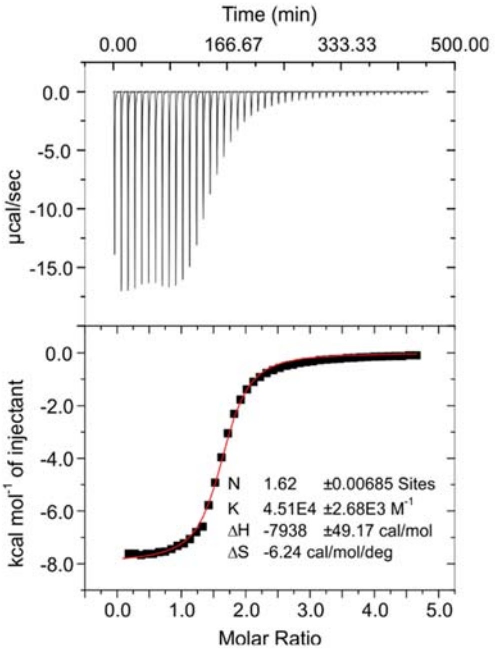

Figure S 35. Top- Traces of the raw data (heat vs time) of the ITC experiment of cage **1** ([cell] =1 × 10^{-3} M) with **5** ([syringe] =2.0 × 10^{-2} M). The solutions were prepared using a chloroform: acetonitrile 9:1 solvent mixture. Bottom- Normalized integrated heat (black squares) vs. **5**/**1** molar ratio. The experimental data were fit to one set of sites binding model (red line).

3.5. Pair-wise inclusion of pyridine *N*-oxides 4a and 5 in 1.

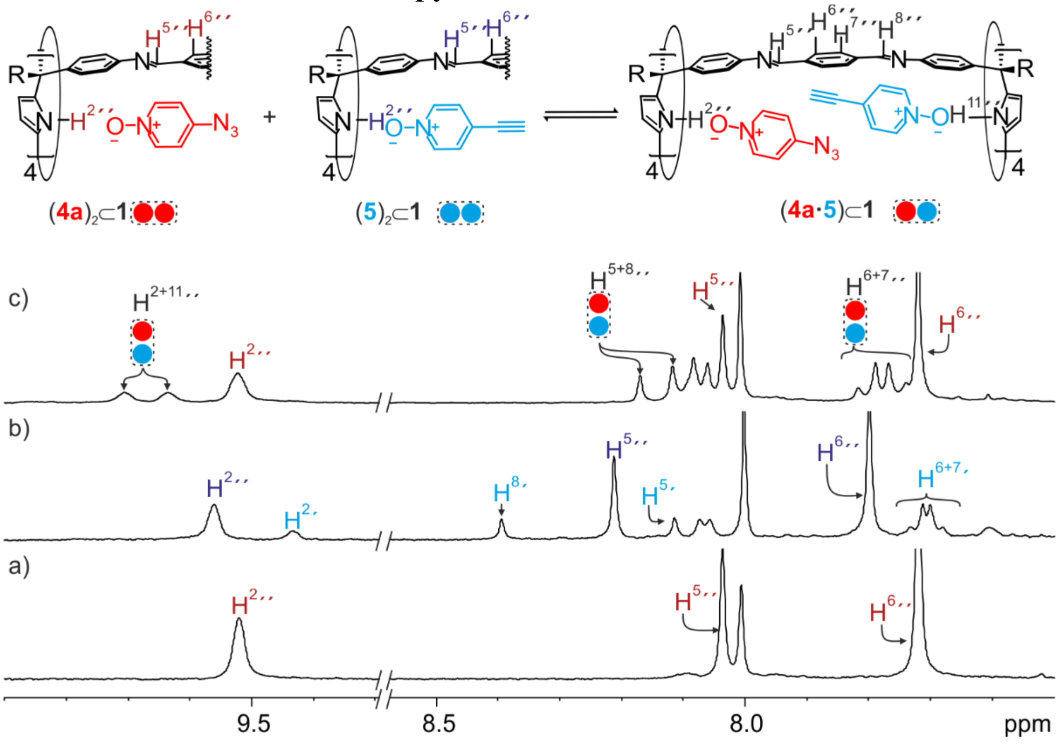

Figure S 36. Selected regions of the ^{1}H NMR (400 MHz, 298 K, CDCl3:CD3CN 9:1) spectra of a) 2:1 mixture of **1** and **4a**, b) 2:1 mixture of **1** and **5**, and c) 1:2:2 mixture of **1:4a:5**, respectively. Primed and double-primed protons correspond to the 1:1 and 2:1 homo- and hetero-complexes, correspondingly. Proton assignments in blue correspond to complexes with **5** and proton assignments in red to complexes with **4a**.

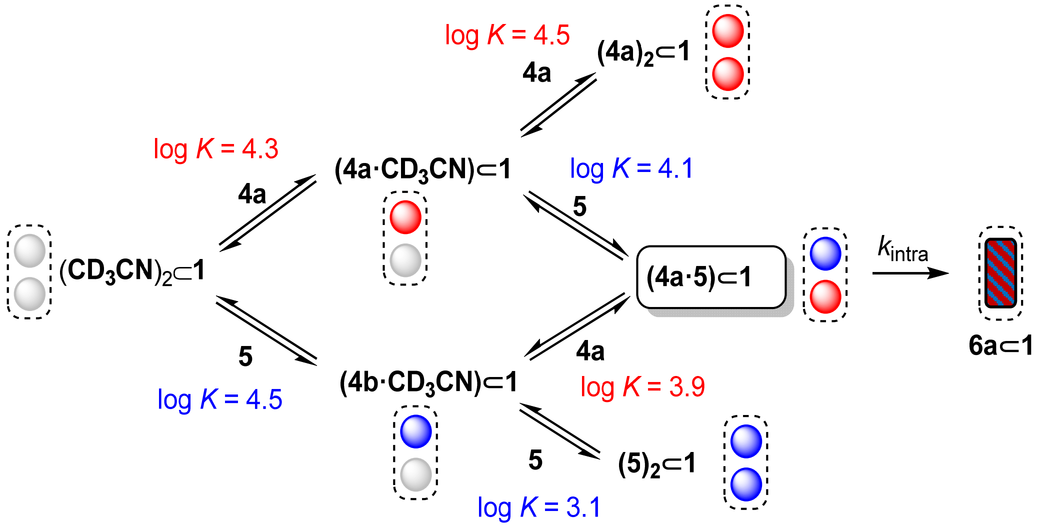

Figure S 37. Theoretical kinetic model used for the non-linear analysis of the experimental data including the values for the thermodynamic equilibrium constants determined/estimated for each binding process. Red spheres correspond to 4-azido pyridine *N*-oxide **4a**, blue spheres correspond to 4-ethynyl pyridine *N*-oxide **5**.

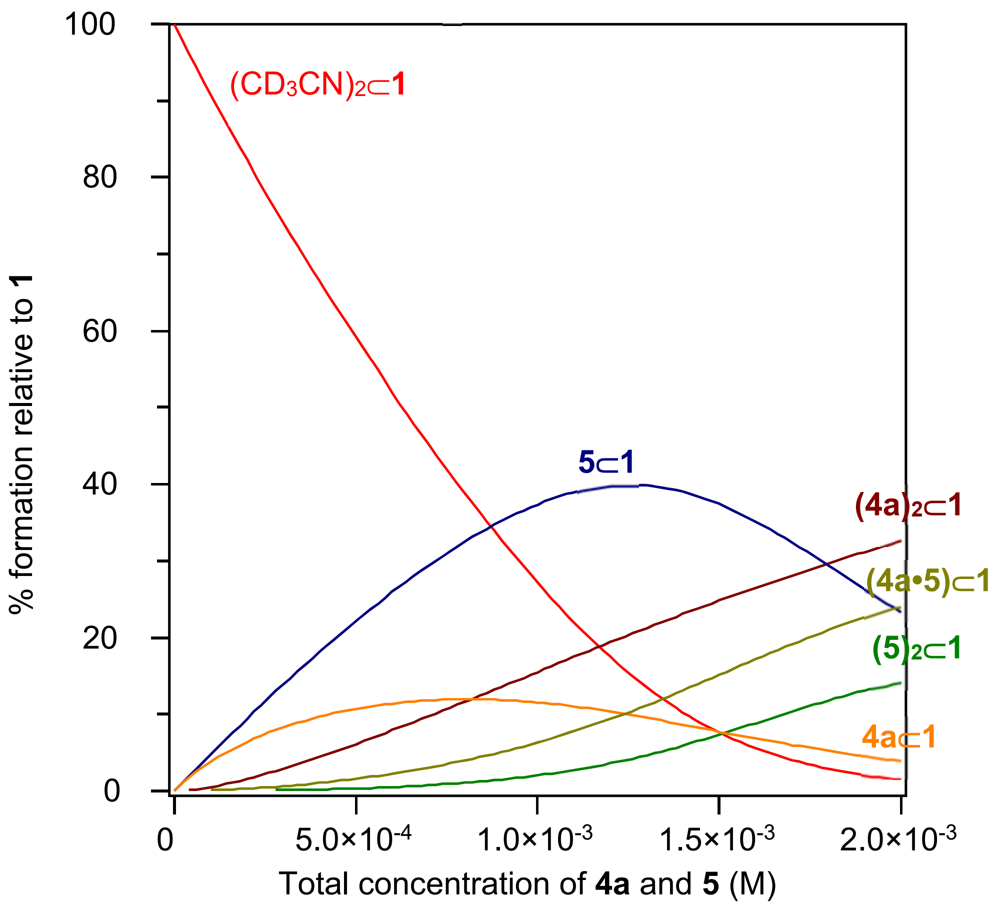

Figure S 38. Simulated speciation profile for octa-imine cage **1** (2 mM) with incremental amounts of **4a** (up to 2 mM) and **5** (up to 2 mM) determined using Hyperquad Simulation and Speciation (HySS2009) software using a model that considers the reversible formation of 1:1 and 2:1 homo- and hetero-inclusion complexes with the constants depicted in **Figure S 37**.

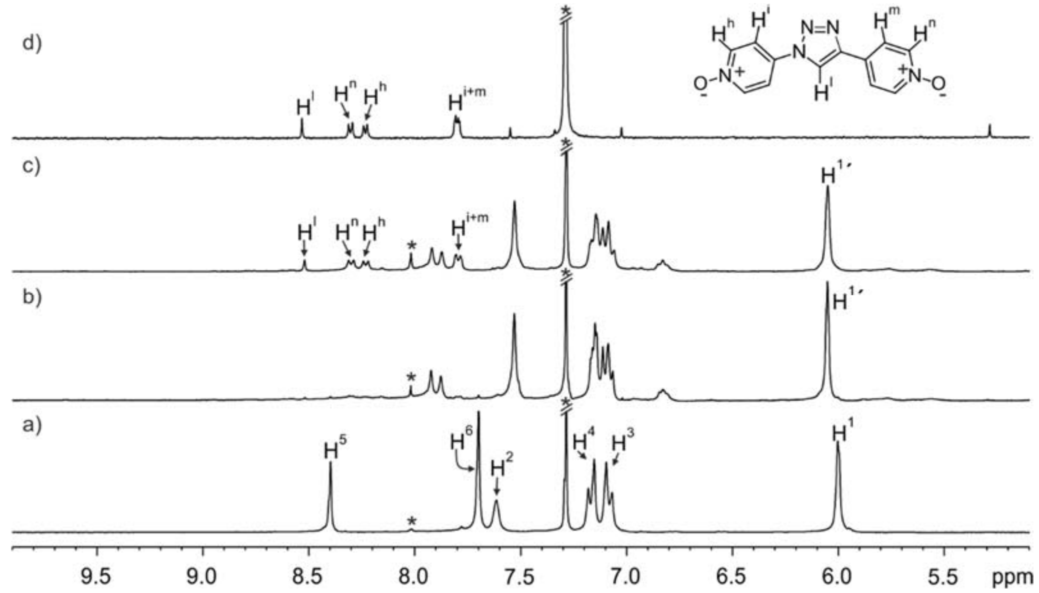

Figure S 39. Selected regions of ^{1}H NMR (400 MHz, at 298 K, $CDCl_3$: CD_3CN 9:1) spectra of a 2 mM solution of octa-imine cage **1** before (a) and after solid-liquid extraction of 1equiv. (b), and 2 equiv. (c) of **6a**. Due to the low solubility of **6a** in $CDCl_3$:CD_3CN 9:1 solvent mixture the specific amount of **6a** was added as MeOH solution in the NMR tube containing octa-imine cage **1**. The solvent was removed under reduced pressure and the resulting solid was re-dissolved in $CDCl_3$:CD_3CN 9:1 solvent mixture and sonicated for 5 min. Spectrum d) corresponds to the free bispyridine *N*-oxide **6a** after 5 min sonication in a $CDCl_3$:CD_3CN 9:1 solvent mixture.

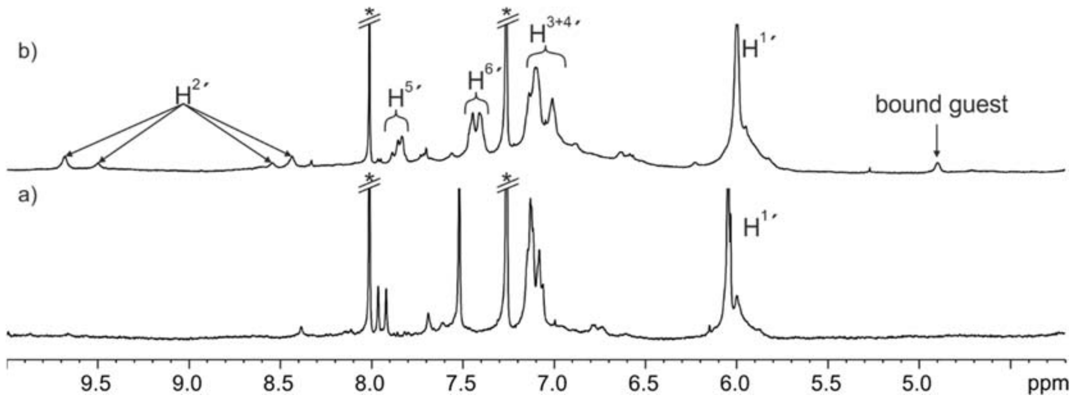

Figure S 40. ^{1}H NMR spectra (500 MHz cryoprobe, $CDCl_3$:CD_3CN 9:1) of a 2 mM solution of octa-imine cage **1** with 1 equiv. of **6a** a) at 298 K, and b) at 233 K.

3.6. Pair-wise inclusion of pyridine *N*-oxides 4b and 5 with in 1.

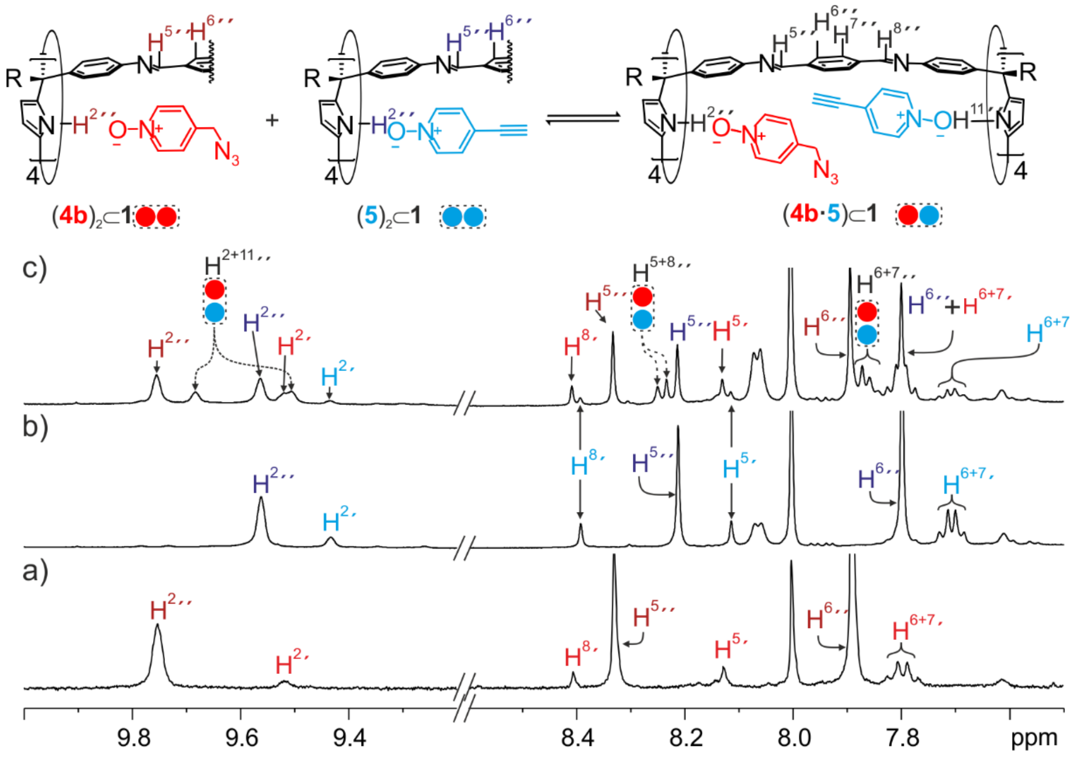

Figure S 41. Selected regions of ^{1}H NMR (400 MHz, 298K, $CDCl_3$:CD_3CN 9:1) spectra of solutions containing: a) 1:2 mixture of cage **1** and **4b**; b) 1:2 mixture of cage **1** and **5**; and c) 1:2:1 mixture of **1:5:4b**. Primed and double primed protons correspond to the 1:1 and 2:1 complexes, correspondingly. Proton assignments in blue correspond to complexes with **5** and proton assignments in red to complexes with **4b**.

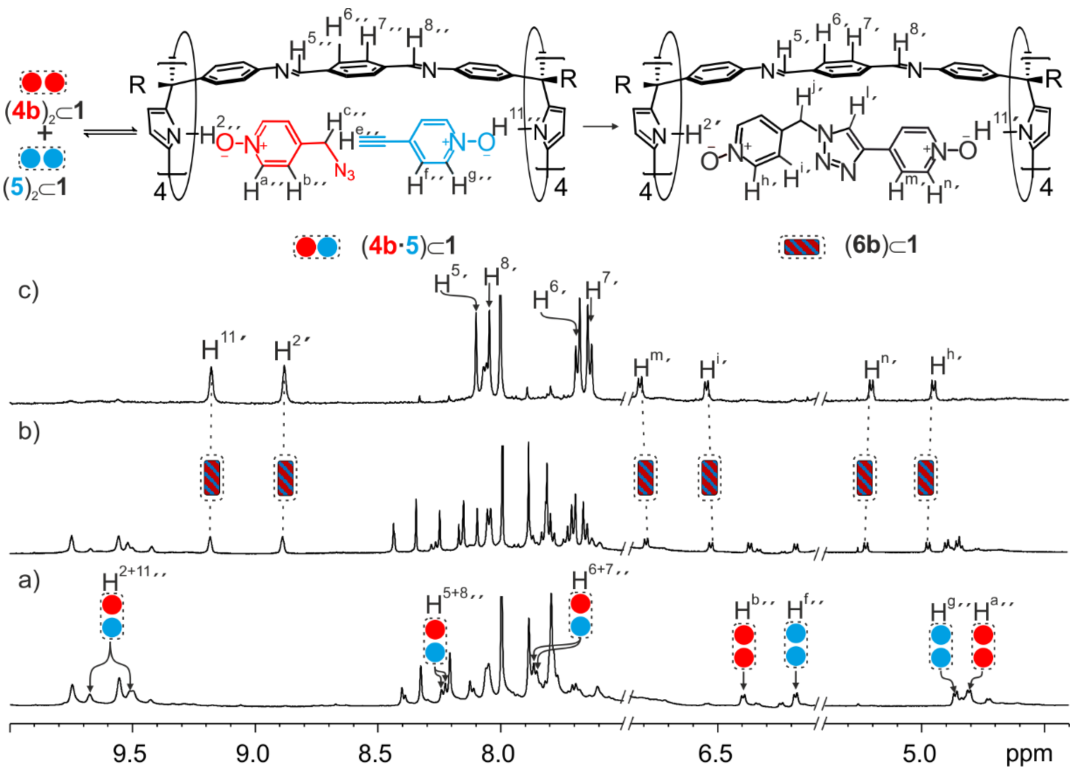

Figure S 42. Top) The 1,3-dipolar cycloaddition reaction between **4b** and **5** included in the octa-imine cage **1** yielded the complex **6b⊂1**. (Bottom) Selected regions of the ^{1}H NMR (300 MHz, 298 K, $CDCl_3$:CD_3CN 9:1) spectra corresponding to the monitoring of the formation of complex **6b⊂1** starting from a 1:1:2 molar mixture of compounds **1**, **4b**, and **5**, respectively, after a) 0 h, b) 8 h, c) 48 h. Primed letters indicate the proton signals in the 1:1 complexes, double-primed letters are used for the 2:1 homo- and hetero-complexes.

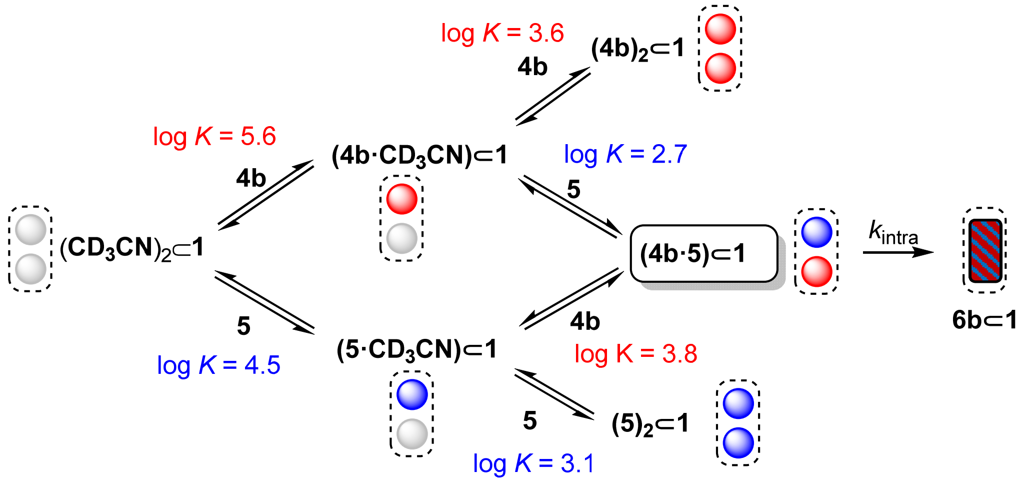

Figure S 43. Theoretical kinetic model used for the non-linear analysis of the experimental data including the values for the thermodynamic equilibrium constants determined/estimated for each binding process. Red spheres correspond to azido-methylpyridine *N*-oxide **4b**, blue spheres correspond to ethynyl pyridine *N*-oxide **5**.

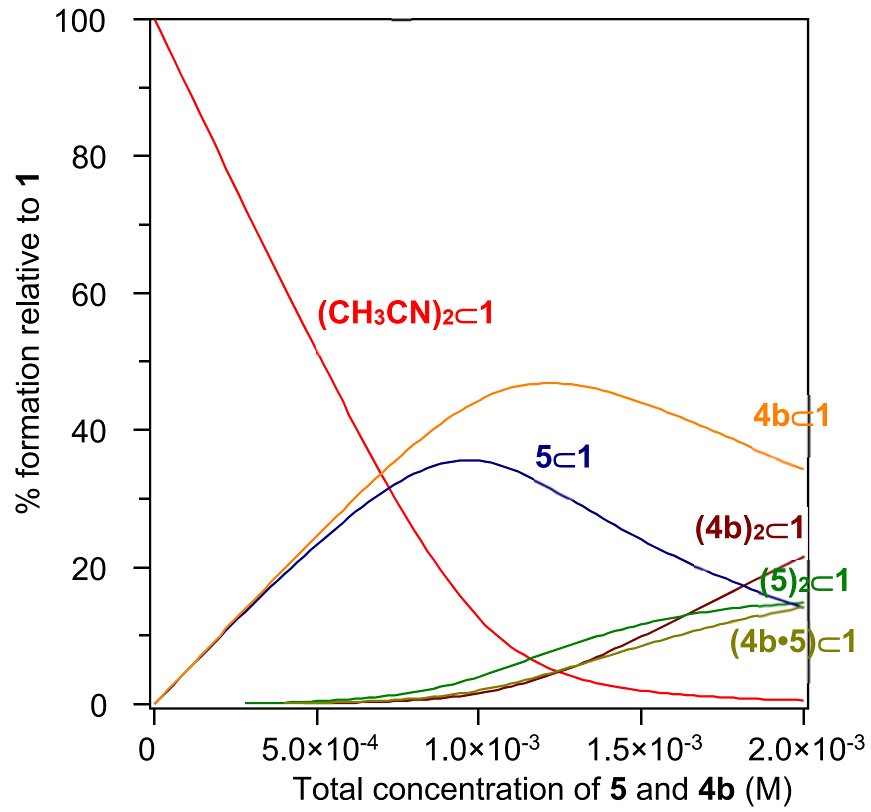

Figure S 44. Simulated speciation profile for octa-imine cage **1** (2 mM) with incremental amounts of **4b** (up to 2 mM) and **5** (up to 2 mM) determined using Hyperquad Simulation and Speciation (HySS2009) software using a model that consider the reversible formation of 1:1 and 2:1 homo- and hetero-inclusion complexes with the constants depicted in **Figure S 43**.

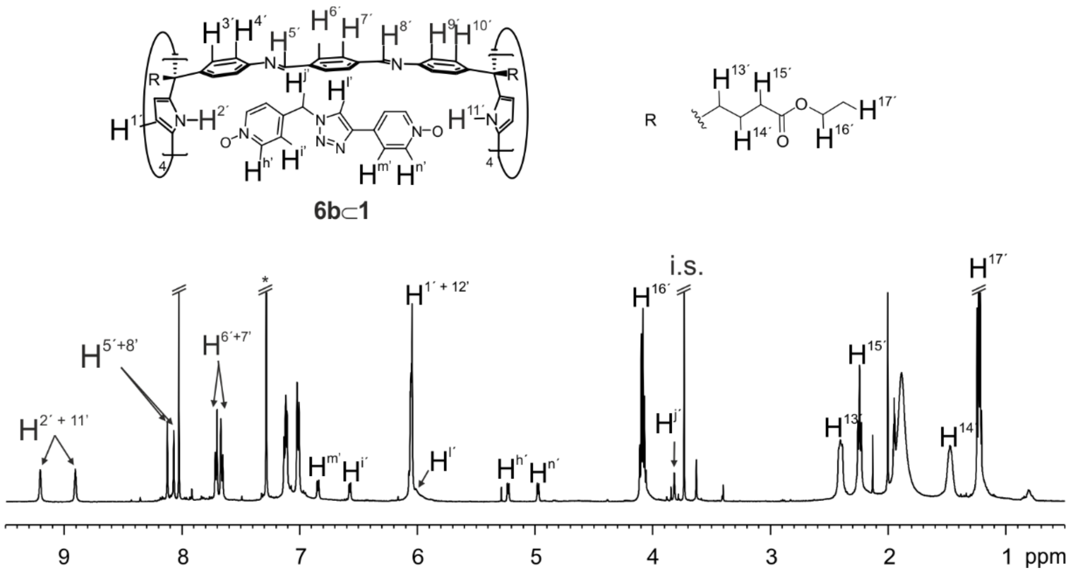

Figure S45. ^{1}H NMR spectrum (500 MHz, 298 K, $CDCl_3$:CD_3CN 9:1) of **6b⊂1** complex obtained from a solid-liquid extraction of the insoluble guest **6b** with a solution of $(CD_3CN)_2$⊂**1**.

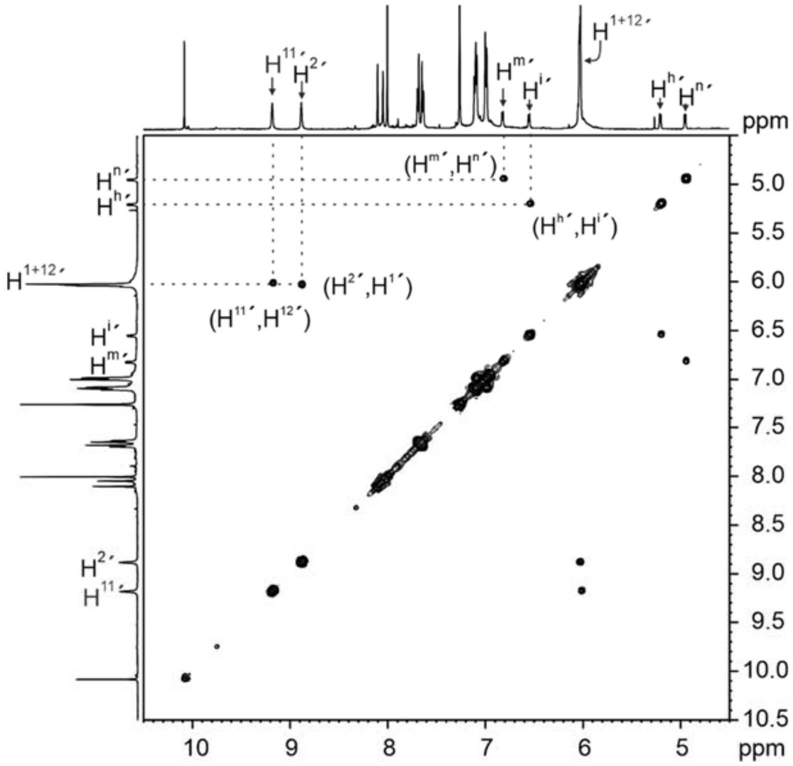

Figure S46. Selected region of the ^1H-^1H COSY NMR (500 MHz, at 298 K, $CDCl_3$: CD_3CN 9:1) of **6b⊂1** complex.

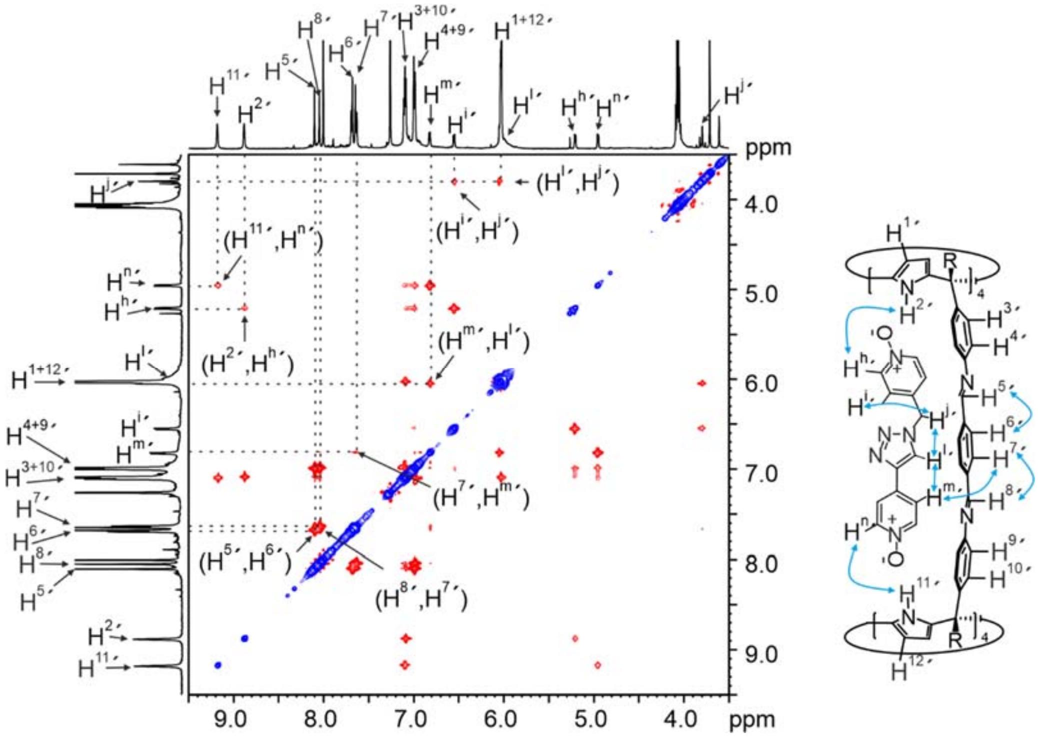

Figure S47. Selected region of the ^1H-^1H ROESY NMR (500 MHz, at 298 K, $CDCl_3$: CD_3CN 9:1, D8 = 0.30 s) of **6b⊂1** complex.

3.7. Pair-wise inclusion of pyridine *N*-oxides 4c and 5 in 1.

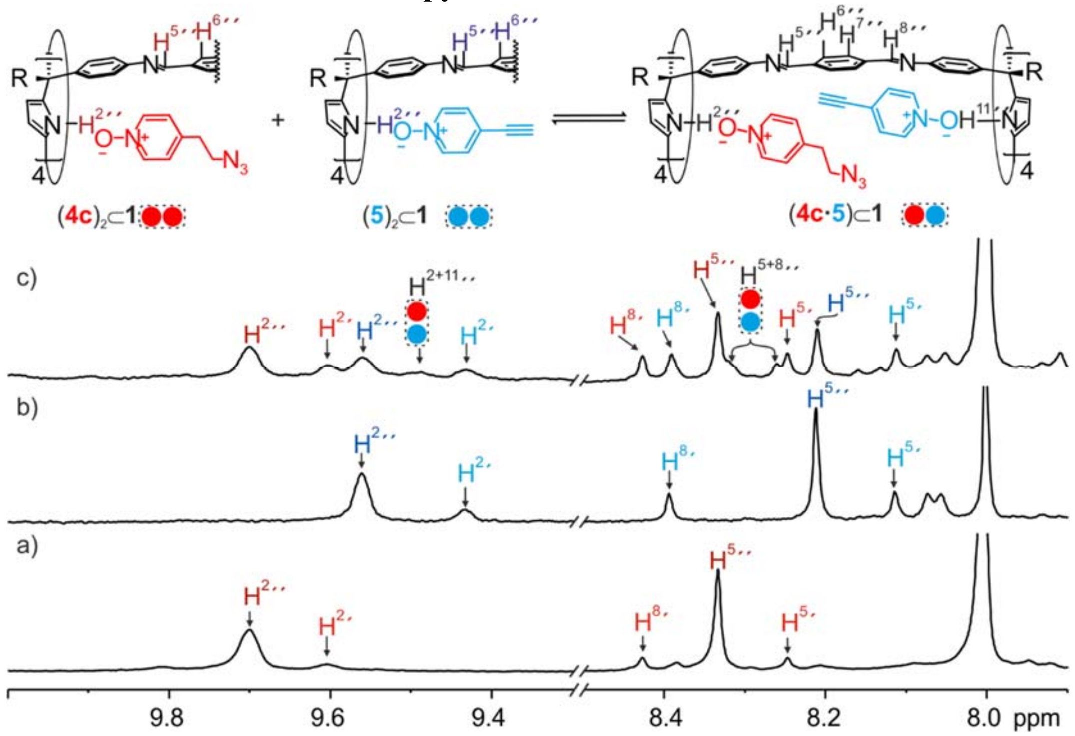

Figure S 48. Selected regions of ^{1}H NMR (400 MHz, 298K, $CDCl_3$:CD_3CN 9:1) spectra of solutions containing: a) 1:2 mixture of cage **1** and **4c**; b) 1:2 mixture of cage **1** and **5**; and c) 1:1:1 mixture of **1:5:4c**. Primed and double primed protons correspond to the 1:1 and 2:1 complexes, correspondingly. Proton assignments in blue correspond to complexes with **5** and proton assignments in red to complexes with **4c**.

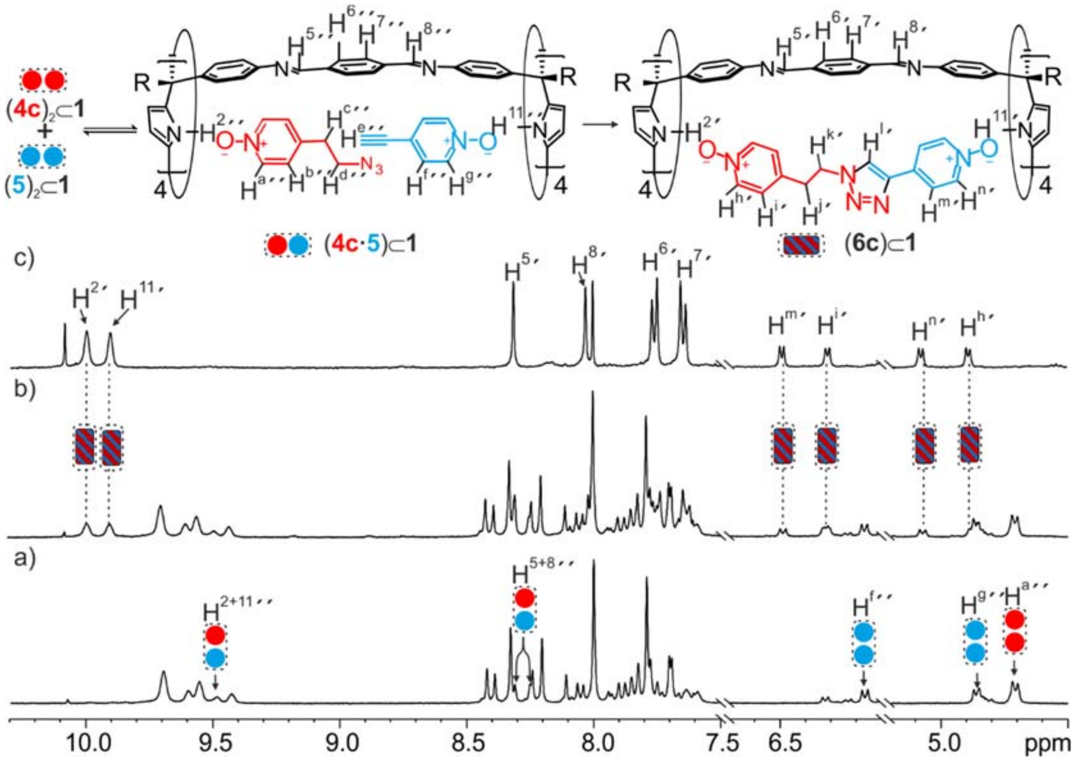

Figure S 49. Top) The 1,3-dipolar cycloaddition reaction between **4c** and **5** included in the octa-imine cage **1** yielded the complex **6c⊂1**. (Bottom) Selected regions of the ^{1}H NMR (300 MHz, 298 K, $CDCl_3$: CD_3CN 9:1) spectra corresponding to the monitoring of the formation of complex **6c⊂1** starting from a 1:1:1 molar mixture of compounds **1**, **4c**, and **5**, respectively, after a) 0 h, b) 36 h. Spectrum c) corresponds to the **6c⊂1** in the same solvent mixture. Primed letters indicate the proton signals in the 1:1 complexes, double-primed letters are used for the 2:1 homo- and hetero- complexes.

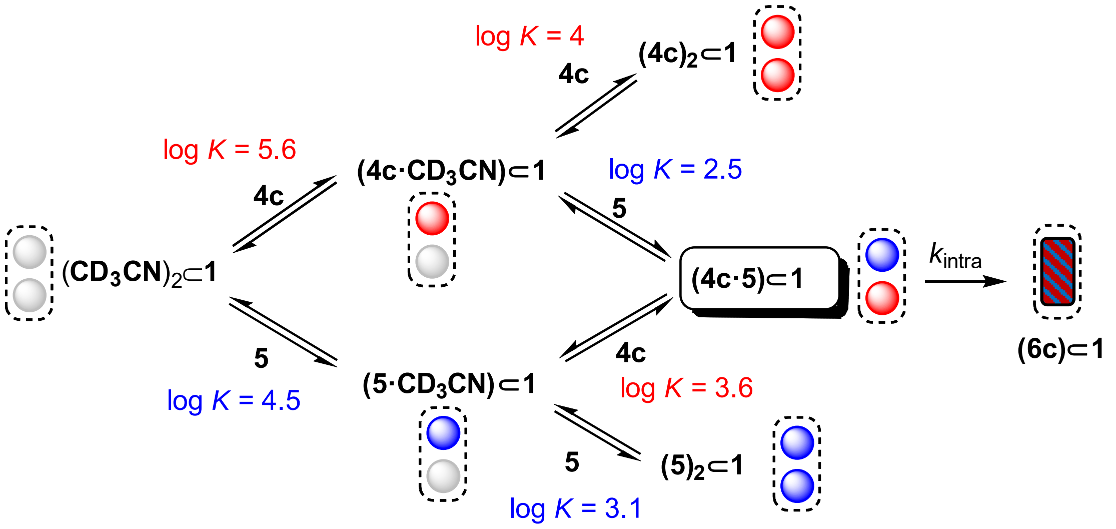

Figure S 50. Theoretical kinetic model used for the non-linear analysis of the experimental data including the values for the thermodynamic equilibrium constants determined/estimated for each binding process. Red spheres correspond to 4-azidoethylpyridine *N*-oxide **4c**, blue spheres correspond to ethynyl pyridine *N*-oxide **5**.

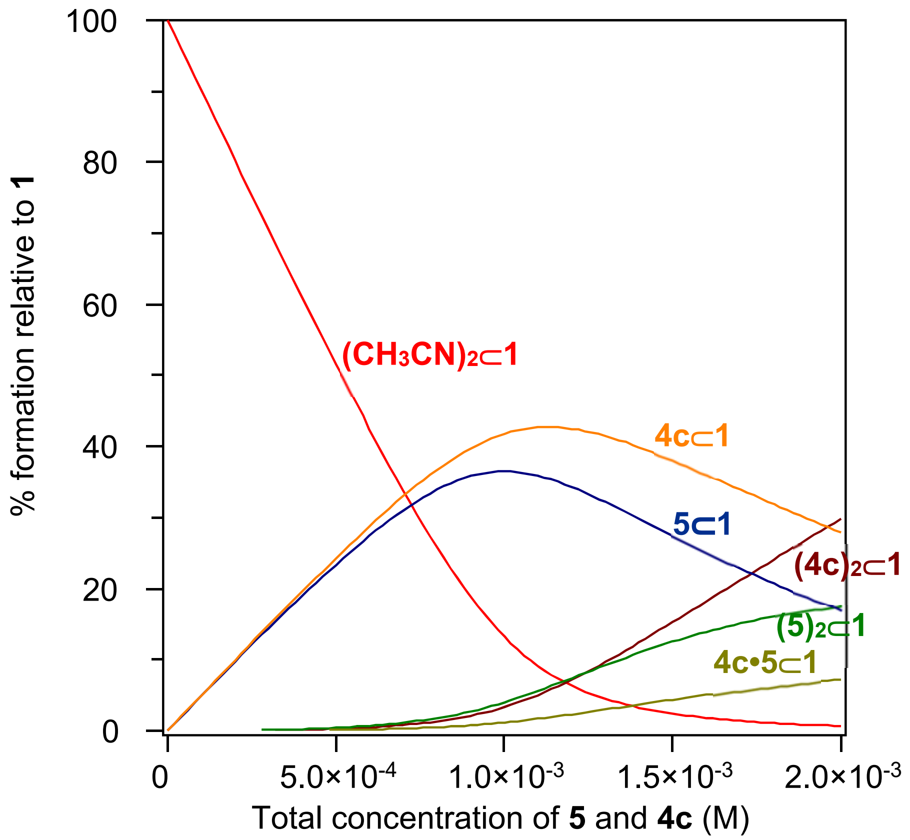

Figure S 51. Simulated speciation profile for octa-imine cage **1** (2 mM) with incremental amounts of **4c** (up to 2 mM) and **5** (up to 2 mM) determined using Hyperquad Simulation and Speciation (HySS2009) software using a model that consider the reversible formation of 1:1 and 2:1 homo- and hetero-inclusion complexes with the constants depicted in **Figure S 50**.

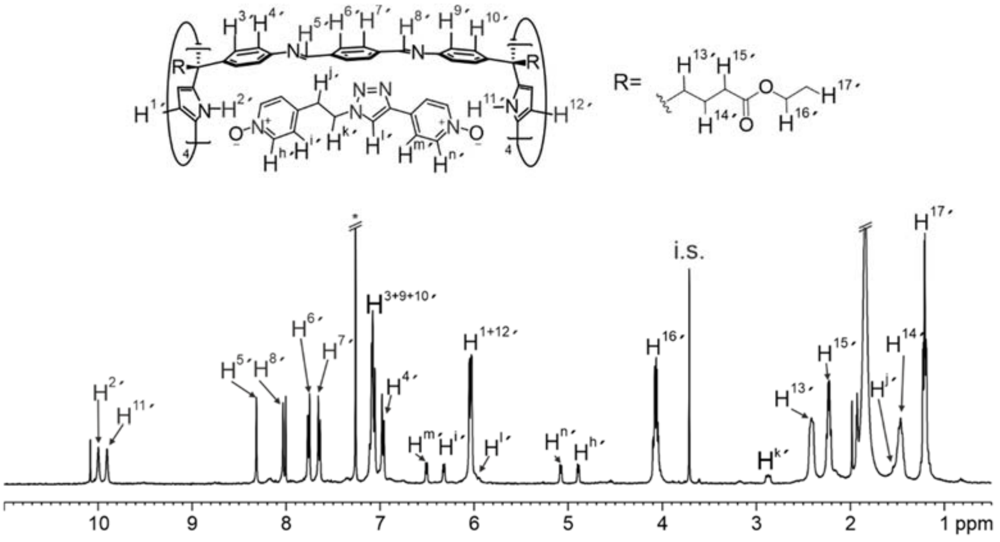

Figure S 52. ^{1}H NMR spectrum (500 MHz, at 298 K, $CDCl_3$: CD_3CN 9:1) of **6c⊂1** complex obtained from a solid-liquid extraction of the insoluble guest **6c** with a solution of $(CD_3CN)_2$⊂**1**.

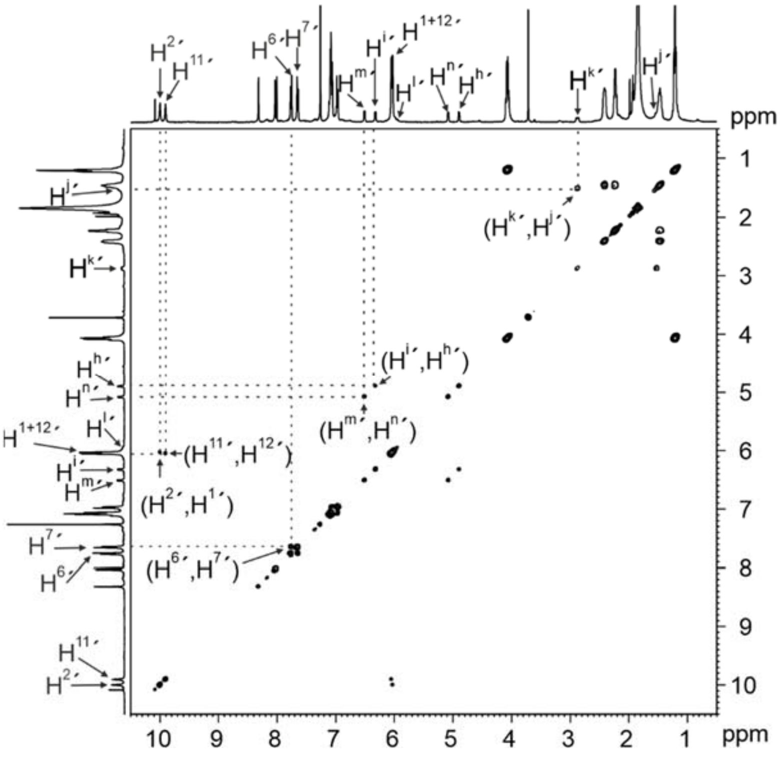

Figure S 53. Selected region of the ^{1}H-^{1}H COSY NMR (500 MHz, 298 K, $CDCl_3$: CD_3CN 9:1) of **6c⊂1** complex.

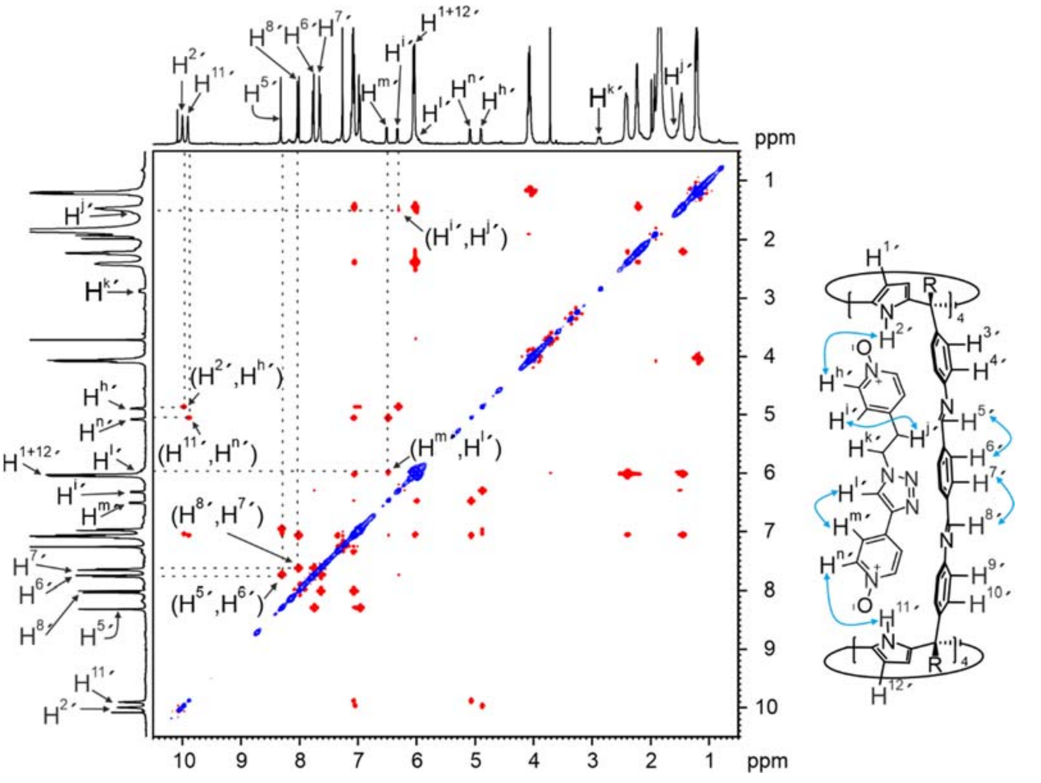

Figure S 54. Selected region of the ^{1}H-^{1}H ROESY NMR (500 MHz, 298 K, $CDCl_3$: CD_3CN 9:1, D8 = 0.30 s) of **6c⊂1** complex.

4. Kinetic characterization of the cycloaddition reaction

4.1. Cycloaddition reactions of 4b and 4c with 5 in octa-imine cage 1.

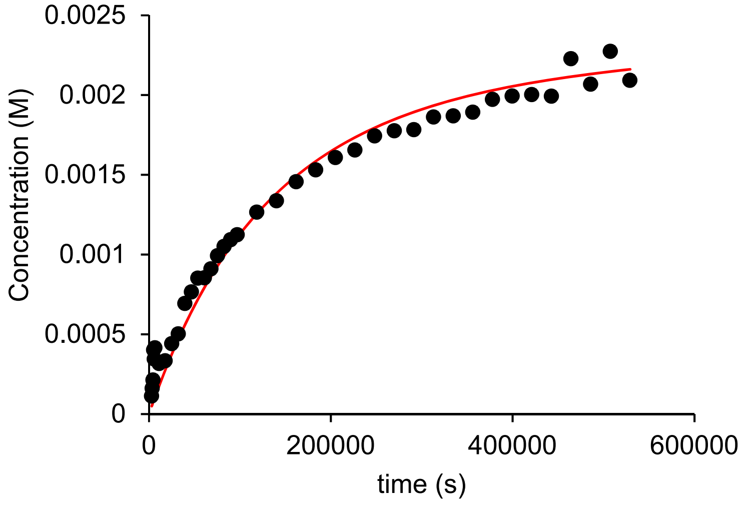

Figure S 55. Changes in concentration of **6b⊂1** complex (black dots) with time starting from a 1:1:1 mixture of **1**, **4b**, and **5**, respectively, in $CDCl_3$:CD_3CN 9:1. Solid red line represents the fit of the experimental kinetic data to the theoretical model using the parameters estimation module of COPASI software Version 4.25. The k_{on}/k_{off} ratio of all binding equilibria were manually fixed based on the determined binding constants. k_{intra} was the only variable parameter used for the fit and returned a value of $k_{intra} = 5.3 \times 10^{-5}$ s^{-1}.

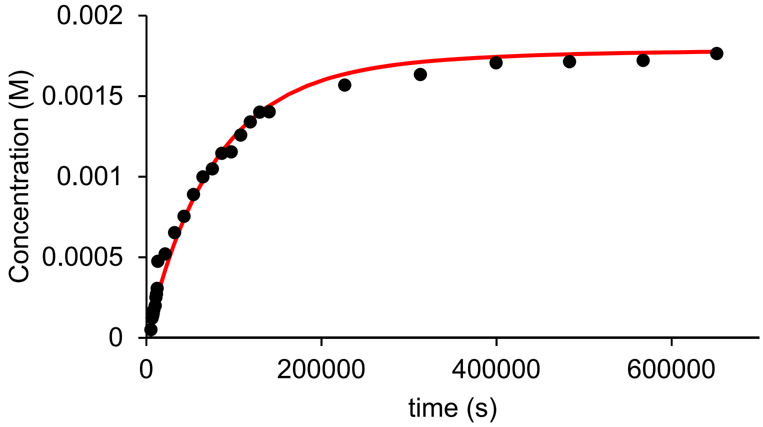

Figure S 56. Changes in concentration of **6b⊂1** complex (black dots) with time starting from a 1:1:2 mixture of **1**, **4b**, and **5**, respectively, in $CDCl_3$:CD_3CN 9:1. Solid red line represents the fit of the experimental kinetic data to the theoretical model using the parameters estimation module of COPASI software Version 4.25. The k_{on}/k_{off} ratio of all binding equilibria was manually fixed based on the determined binding constants. k_{intra} was the only variable parameter used for the fit and returned a value of $k_{intra} = 6.3 \times 10^{-5}$ s^{-1}.

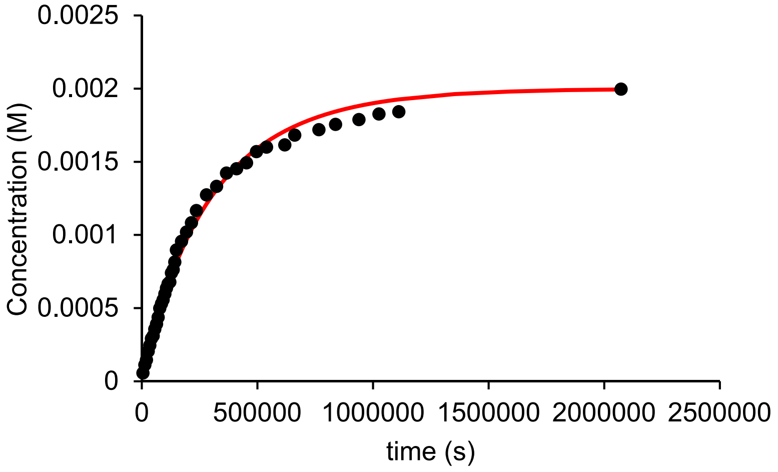

Figure S 57. Changes in concentration of **6b⊂1** complex (black dots) with time starting from a 1:5:5 mixture of **1**, **4b**, and **5**, respectively, in $CDCl_3$:CD_3CN 9:1. Solid red line represents the fit of the experimental kinetic data to the theoretical model using the parameters estimation module of COPASI software Version 4.25. The k_{on}/k_{off} ratio of all binding equilibria was manually fixed based on the determined binding constants. k_{intra} was the only variable parameter used for the fit and returned a value of $k_{intra} = 2.6 \times 10^{-5}$ s^{-1}.

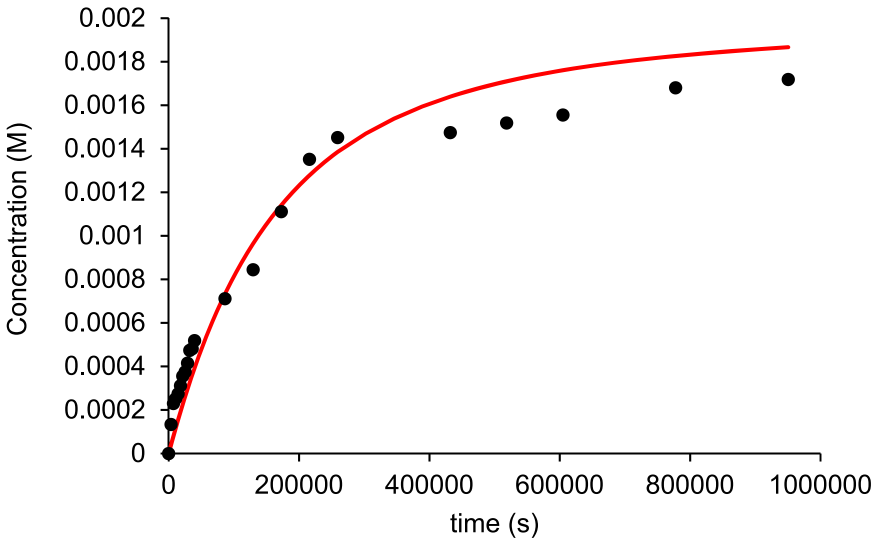

Figure S 58. Changes in the concentration of **6c⊂1** complex (black dots) with time starting from a 1:1:1 mixture of **1**, **4c**, and **5**, respectively, in $CDCl_3$:CD_3CN 9:1. Solid red line represents the fit of the experimental kinetic data to the theoretical model using the parameters estimation module of COPASI software Version 4.25. The k_{on}/k_{off} ratio of all binding equilibria was manually fixed based on the determined binding constants. k_{intra} was the only variable parameter used for the fit and returned a value of $k_{intra} = 8.1 \times 10^{-5}$ s^{-1}.

4.2. Cycloaddition reactions of 4a, 4b, and 4c with 5 in the bulk.

We carried out the cycloaddition reactions in the bulk in chloroform:acetonitrile 9:1 solvent mixture. We used 4-methylpyridine *N*-oxide as the internal standard (i.s.) (1 mM) which showed a distinct retention time compared to either reactants **4a-d** or products **6a-c** and **7a-c**. Four different concentrations of **6a-c** (0.1, 0.05, 0.02, 0.01 mM) were used for the calibration curve ($A_{product}/A_{i.s.}$). We assume that the 1,4-isomers (**6a-c**) have similar extinction coefficient than the 1,5-counterparts (**7a-c**).

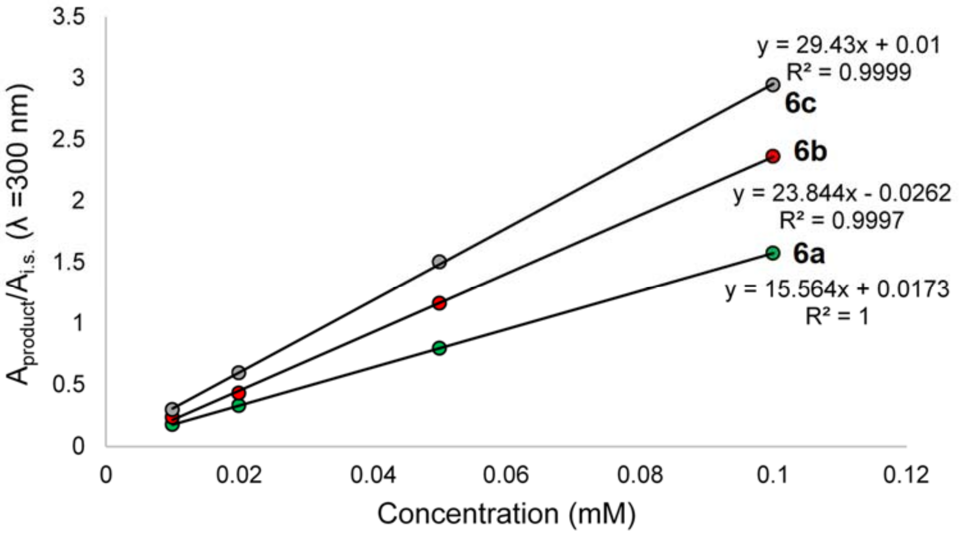

Figure S 59. Calibration curves of 1,4-cycloaddition products **6a-c** using 4-methyl pyridine *N*-oxide as i.s. (1 mM) and considering ($A_{product}/A_{i.s.}$) ratio at 300 nm.

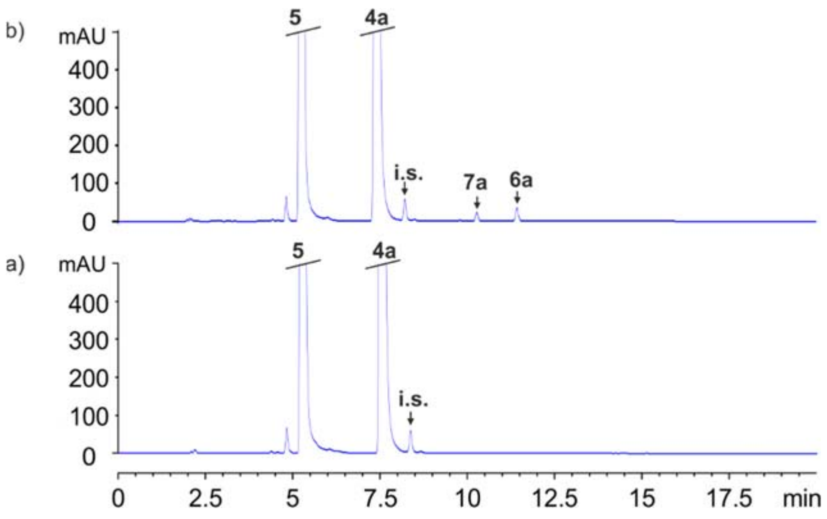

Figure S 60. HPLC trace of the reaction crude between **4a** and **5** ($CHCl_3$:CH_3CN 9:1, 25 mM for each and r.t.) a) 0h and b) 361 h. Both isomers **6a** and **7a** were detected in the reaction mixture. 4-methyl pyridine *N*-oxide was used as internal standard (i.s.) (1 mM).

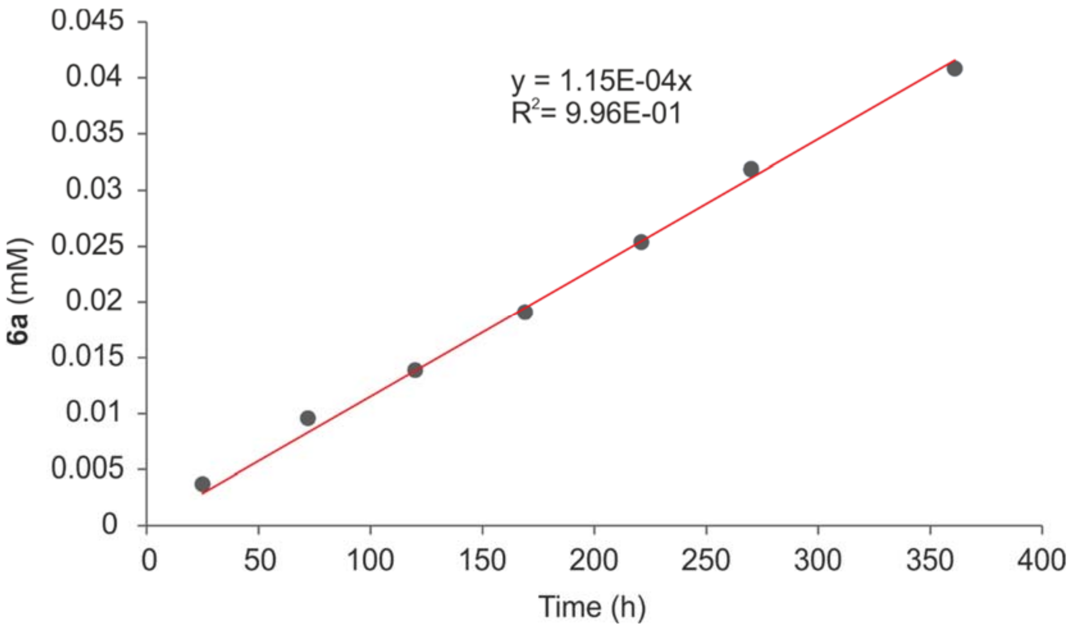

Figure S 61. Changes in the concentration of **6a** (black dots) in the with time (t < 400 s) starting from an equimolar 25 mM mixture of **4a** and **5**, in $CHCl_3:CH_3CN$ 9:1. Solid red line represents the linear fit of the experimental kinetic data to a second order irreversible reaction used to determine the initial reaction rate.

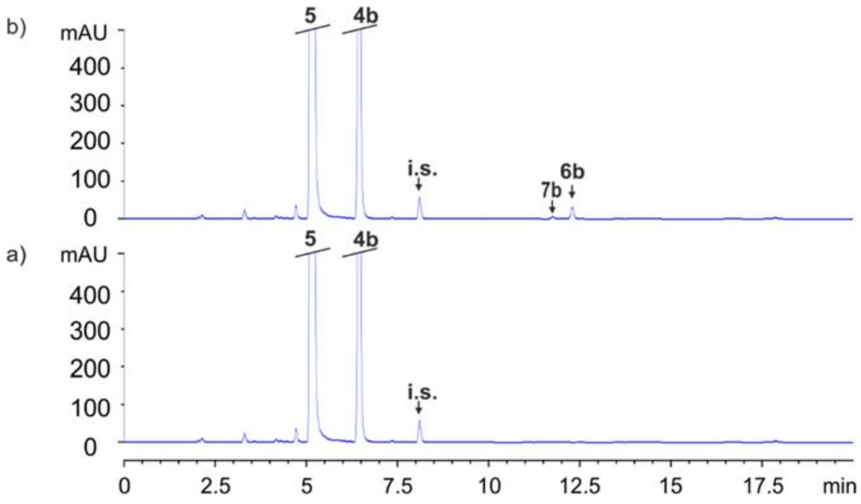

Figure S 62. HPLC trace of the reaction crude between **4b** and **5** ($CHCl_3:CH_3CN$ 9:1, 25 mM for each and r.t.) a) 0h and b) 361 h. Both isomers **6b** and **7b** were detected in the reaction mixture. 4-methyl pyridine *N*-oxide was used as internal standard (i.s.) (1 mM).

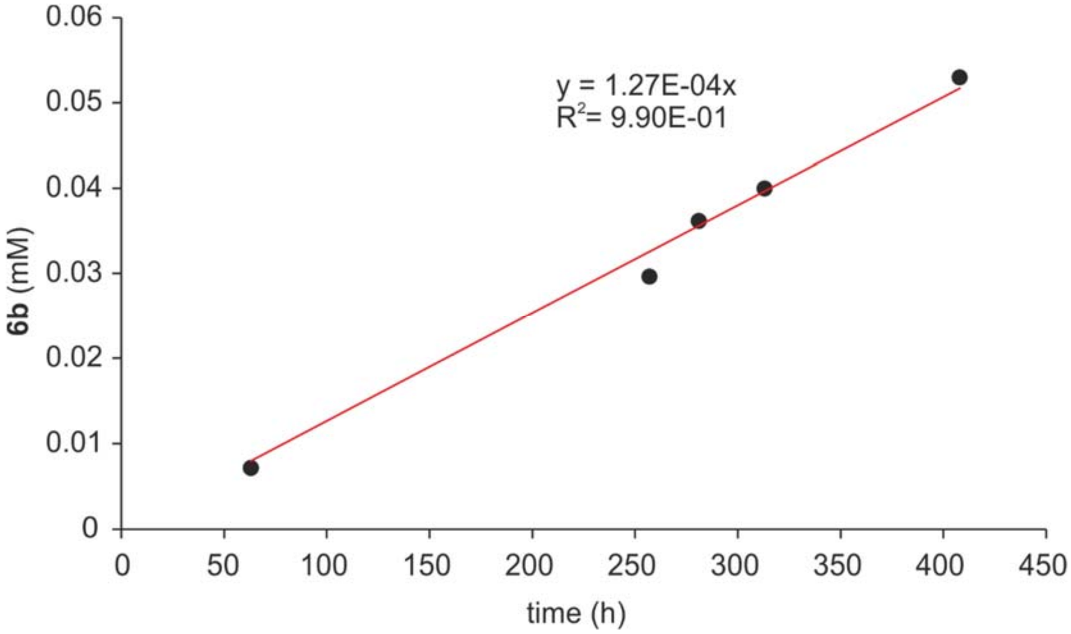

Figure S 63. Changes in the concentration of **6b** (black dots) with time (t < 400 s) starting from an equimolar 25 mM mixture of **4b** and **5**, in $CHCl_3$:CH_3CN 9:1. Solid red line represents the linear fit of the experimental kinetic data to a second order irreversible reaction used to determine the initial reaction rate.

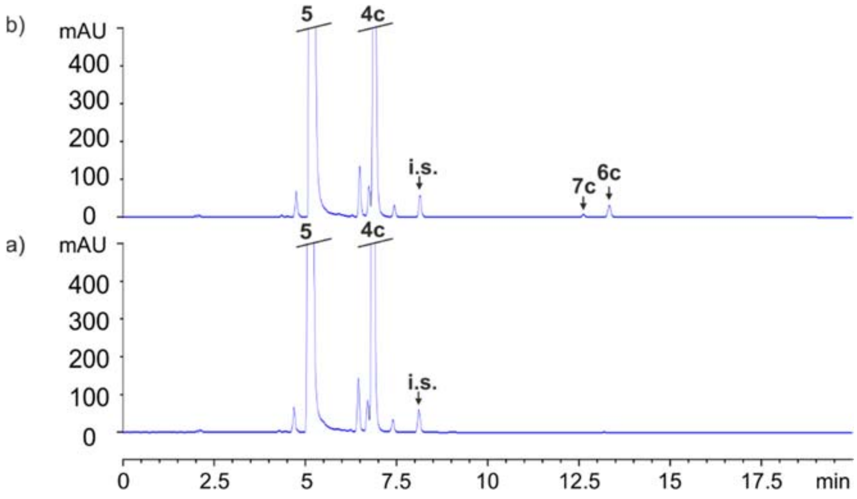

Figure S 64. HPLC trace of the reaction crude between **4c** and **5** ($CHCl_3$:CH_3CN 9:1, 25 mM for each and r.t.) a) 0h and b) 361 h. Both isomers **6c** and **7c** were detected in the reaction mixture. 4-methyl pyridine *N*-oxide was used as internal standard (i.s.) (1 mM).

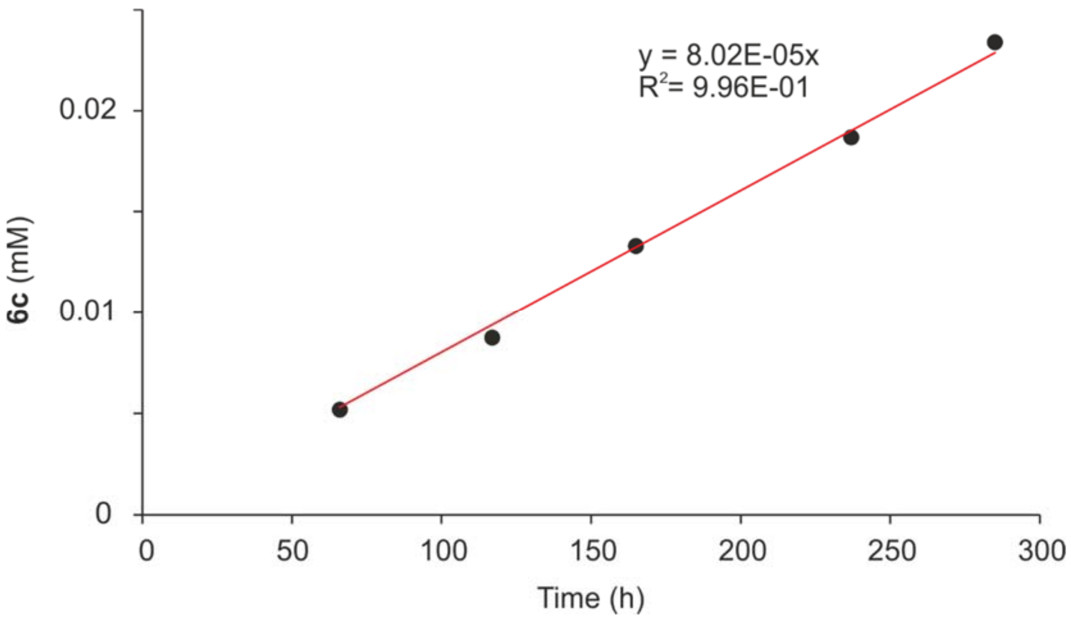

Figure S 65. Changes in the concentration of **6c** (black dots) with time (t < 400 s) starting from an equimolar 25 mM mixture of **4c** and **5**, in $CHCl_3$:CH_3CN 9:1. Solid red line represents the linear fit of the experimental kinetic data to a second order irreversible reaction used to determine the initial reaction rate.

Table S 1. Summary of initial reaction rates and rate constants of the formation of 1,4-isomers of the cycloaddition product from the corresponding azido and ethynyl precursors (25 mM each) in a $CHCl_3$:CH_3CN 9:1 solvent mixture.

	v_0 (M/s)	k_{bulk} ($M^{-1}s^{-1}$)
**6a**	3.19×10^{-11}	5.1×10^{-8}
**6b**	3.5×10^{-11}	5.6×10^{-8}
**6c**	2.2×10^{-11}	3.6×10^{-8}

5. Control experiments

We performed a series of experiments with different substrates (**Figure S67**) to support the role of the cavity on the acceleration observed for the 1,3-dipolar cycloaddition in the presence of octa-imine **1.** The results are summarized in **Table S 2**.

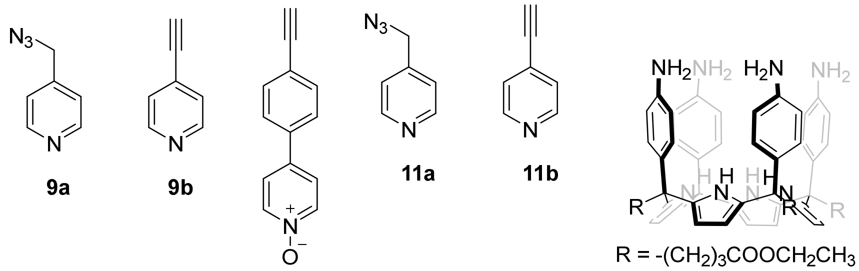

9a **9b** **10** **11a** **11b**

R = $-(CH_2)_3COOCH_2CH_3$

2

Figure S 66. Molecular structures of the substrates **9a**, **9b, 10**, **11a**, **11b**, and calix[4]pyrrole **2** used for the control experiments to support the importance of the cavity of octa-imine cage **1** in the acceleration of the cycloaddition reaction.

Table S 2. Summary of the kinetic experiments performed using octa-imine **1** or tetra-amine calix[4]pyrrole **2** and different substrates.

**Entry**	**Container**	**Substrates** (equimolar)	**Product**	$\mathbf{k_{(intra)} \times 10^5}$ ($\mathbf{s^{-1}}$)	**EM (M)**
1	**1**	**4b, 5**	**6b**	~ 5 (± 1)	~ 10^3
2	**1**	**4c, 5**	**6c**	~ 8 (± 1)	~ 10^3
3	**1**	**9a, 9b**[a]	n.d.[d]	-	
4	**2**[b]	**9a**[a]**, 5**	n.d.[d]	-	
5	**1**	**4b, 10**[c]	n.d.[d]	-	
6	**1**	**11a, 11b**[a]	n.d.[d]	-	-
7	**1**	**4b, 10**[c]**, 5**	**6b**	Not determined	
8	**1**	**4a, 5, 4b**	**6b**	Not determined	

[a]The pyridine and phenyl derivatives do not have intrinsic affinity for the C[4]P hemispheres, and therefore they do not significantly bind the cavity of **1** or **2**. [b]K[(**2** + **5** ⇌ 5⊂**2**] >10^4 M^{-1}. [c]The 4-(4'-ethynylphenyl) pyridine *N*-oxide guest **9** is too large to be co-included with the azido derivative **4b** or **5** in the cavity of **1**. [d]n.d. not detected after 7 days.

From the tabulated data we can also derive the following conclusions: 1) octa-imine cage **1** did not perform as a conventional organo-catalyst (entry 3, 4 and 6); 2) the formation of the ternary complex of the two reacting substrates was mandatory for the acceleration of the reaction (entry 1, 2, 5, 6 and 7); 3) octa-imine cage **1** showed selectivity for the mediation of the 1,3-dipolar cycloaddition of **5** with substrate **4b** over **4a** (entry 8).

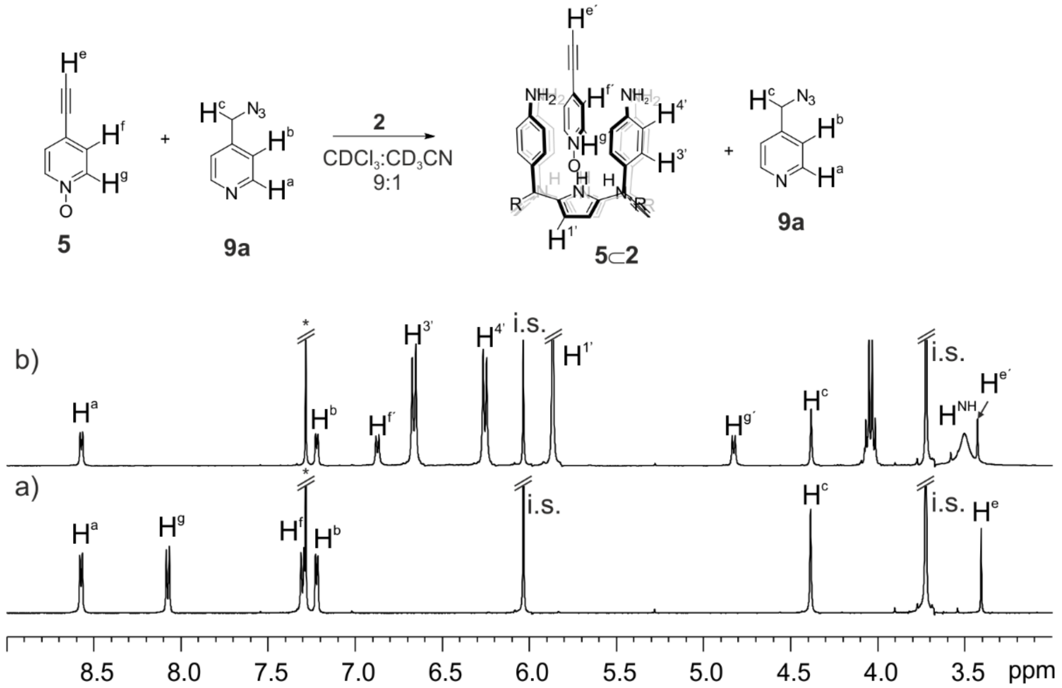

Figure S67. Selected region of the ^{1}H NMR spectra (400 MHz, at 298 K, CDCl$_3$:CD$_3$CN 9:1) of a) **5**:**9a** 1:1 2 mM mixture; and b) **5**:**9a**:**2** 1:1:1 2 mM mixture. No changes were detected in the ^{1}H NMR spectra of the mixtures after 1 month, suggesting that tetra-amino AE-C[4]P **2** does not act as a catalyst in the 1,3-dipolar cycloaddition reaction.

6. DFT Calculations

We performed DFT theoretical calculations, at the RI[6,7,8] BP86[6] D3BJ[9],[10]/def-SV(P)[11],[12] level of theory using Turbomole v7.0 of homo-capsular complexes (**4a**)$_2\subset$1, (**5**)$_2\subset$1, and (**4b**)$_2\subset$1, and ternary hetero-capsular complexes (**4a·5**)$\subset$**1**, (**4b·5**)$\subset$**1**, and (**4c·5**)$\subset$**1**. Moreover, we computed complexes **6a**$\subset$**1** (both isomers **6a**$^{C}\subset$**1** and **6a**$^{N}\subset$**1**), **6b**$\subset$**1**, **7b**$\subset$**1**, and **6c**$\subset$**1.** The results are summarized in **Table S 3**.

All dataset collection of computational results of this manuscript is available in the ioChem-BD repository [13] and can be accessed through this link http://dx.doi.org/10.19061/iochem-bd-1-358.

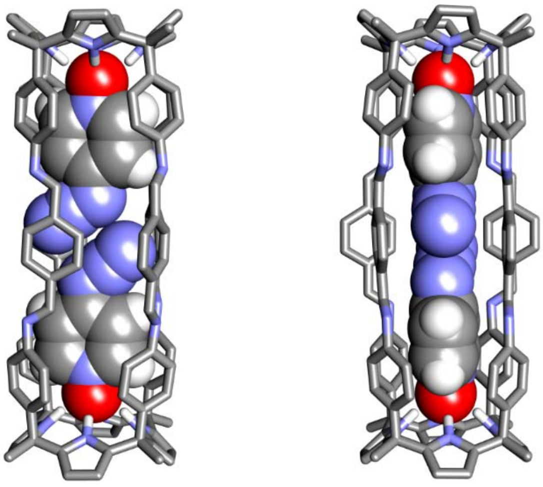

Figure S 68. Two views of the energy-minimized structure of the 2:1 homo-complex (**4a**)$_2\subset$**1.**

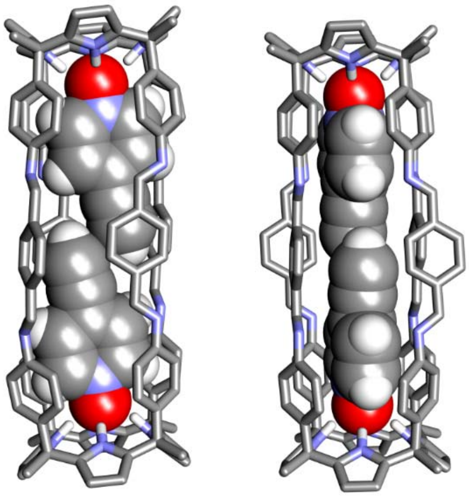

Figure S 69. Two views of the energy-minimized structure of the 2:1 homo-complex (**5**)$_2\subset$**1.**

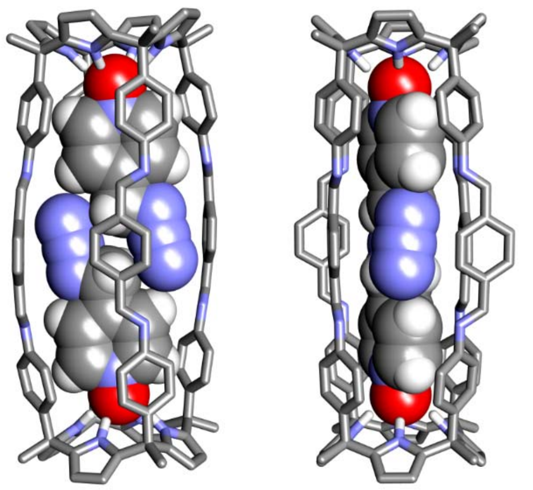

Figure S 70. Two views of the energy-minimized structure of the 2:1 homo-complex (**4b**)$_2$⊂**1.**

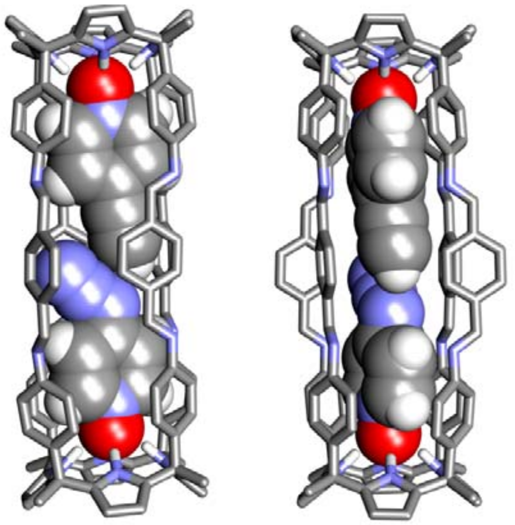

Figure S 71. Two views of the energy-minimized structures of the a) 2:1 hetero-complex (**4a•5**)⊂**1** .

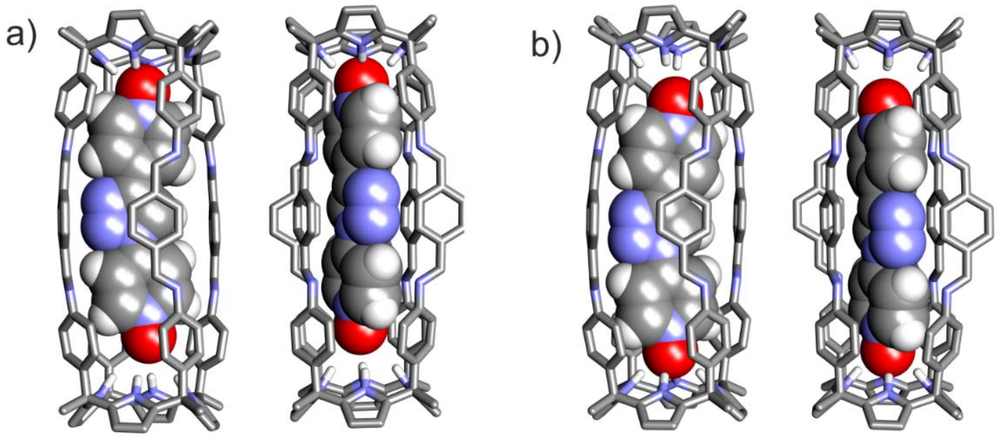

Figure S 72. Two views of the energy-minimized structures of the two isomers of complex **6a⊂1**: a) **6a^{C}⊂1** and b) **6a^{N}⊂1**.

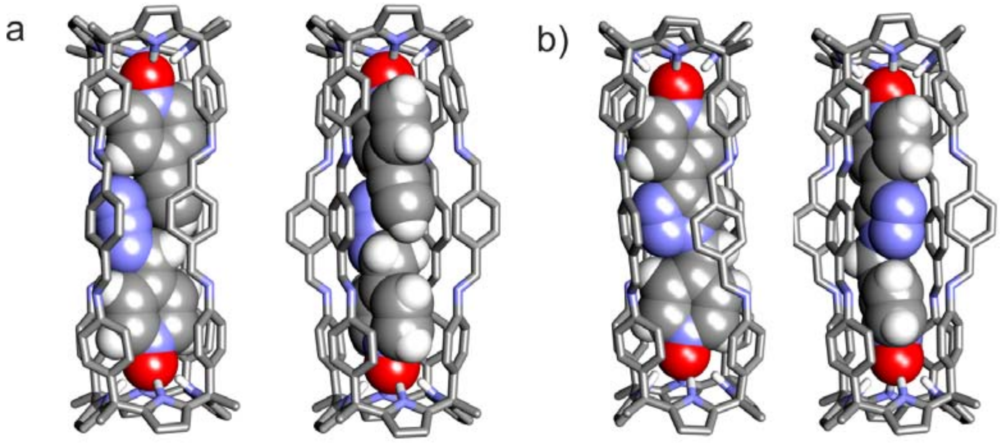

Figure S 73. Two views of the energy-minimized structures of the a) 2:1 hetero-complex (**4b•5**)⊂**1** and b) **6b⊂1** complex.

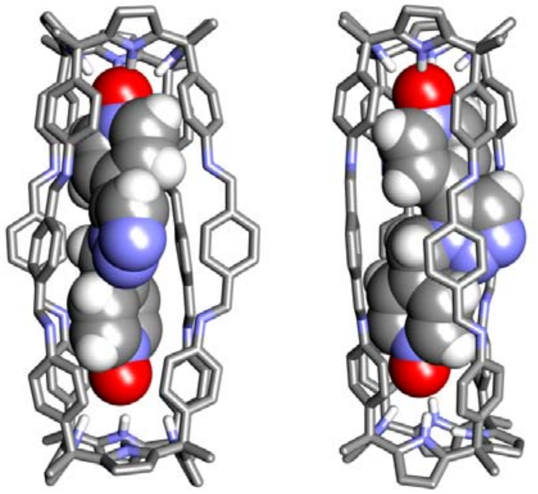

Figure S 74. Two views of the energy-minimized structures of the **7b**⊂**1** complex.

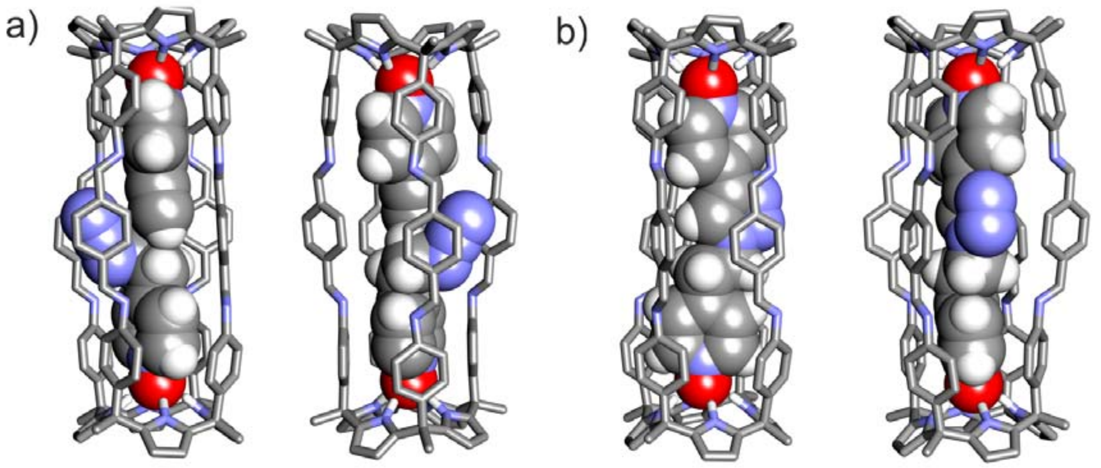

Figure S 75. Two views of the energy-minimized structures of the a) 2:1 hetero-complex (**4c•5**)⊂**1** and b) **6c**⊂**1** complex.

Table S 3. Calculated electronic energies of the homo- and hetero-capsular complexes in the gas phase.

Complex	E (Hartrees)	ΔE[a] (kcal·mol^{-1})
**(4a)$_2$⊂1**	-6786.59613	-64.8
**(5)$_2$⊂1**	-6611.76904	-59.4
**(4b)$_2$⊂1**	-6865.16661	-74.8
**(4a·5)$_2$⊂1**	-6699.18621	-64.3
**(4b·5)⊂1**	-6738.46808	-67.3
**(4c·5)⊂1**	-6777.748942	-62.2
**6a^C⊂1**	-6699.26292	-39.9
**6a^N⊂1**	-6699.26252	-39.7
**6b⊂1**	-6738.57473	-56.1
**7b⊂1**	-6738.51162	-16.8
**6c⊂1**	-6777.87920	-67.6
**(CH_3CN)$_2$⊂1**	-6078.29164	-
**4a**	-486.75732	-
**5**	-399.34809	-
**4b**	-526.03455	-
**4c**	-565.32340	-
**6a**	-886.22098	-
**6b**	-925.50711	-
**6c**	-964.79321	-
**7b**	-925.50656	-

[a]$\Delta E = (E_{complex} + 2 \times E_{CH3CN}) - (E_{(CH3CN)2\subset 1} + E_{guest1} + E_{guest2})$

7. References

[1] L. Escobar, F. A. Arroyave and P. Ballester, *Eur. J. Org. Chem.*, 2018, **2018**, 1097-1106.

[2] L. Adriaenssens, J. L. Acero Sánchez, X. Barril, C. K. O'Sullivan and P. Ballester, *Chem. Sci.*, 2014, **5**, 4210-4215.

3 M. Piccinno, G. Aragay, F. Y. Mihan, P. Ballester and A. Dalla Cort, *Eur. J. Inorg.* Chem., 2015, **2015**, 2664-2670.

[4] Microcal Origin Data Analysis v7.21, Malvern Instruments Limited.

[5] The normalized integrated data produced a single sigmoidal binding isotherm indicating that the binding cooperativity was not large enough to be detected by ITC. We fit the data to a theoretical binding model considering one set of sites which returned an average value for the binding constant of the two sites.

[6] J. P. Perdew, *Phys. Rev. B*, 1986, **33**, 8822-8824.

[7] K. Eichkorn, F. Weigend, O. Treutler and R. Ahlrichs, *Theor. Chem. Acc.*, 1997, **97**, 119-124.

[8] M. Sierka, A. Hogekamp and R. Ahlrichs, *J. Chem. Phys.*, 2003, **118**, 9136-9148.

[9] S. Grimme, S. Ehrlich and L. Goerigk, *J. Comput. Chem.*, 2011, **32**, 1456-1465.

[10] S. Grimme, J. Antony, S. Ehrlich and H. Krieg, *J. Chem. Phys.*, 2010, **132**.
[11] D. Rappoport and F. Furche, *J. Chem. Phys.*, 2010, **133**.
[12] A. Schäfer, H. Horn and R. Ahlrichs, *J. Chem. Phys.*, 1992, **97**, 2571-2577.
[13] M. Álvarez-Moreno, C. de Graaf, N. López, F. Maseras, J. M. Poblet and C. Bo, *J. Chem. Inf. Model.*, 2015, **55**, 95-103.
